# A Diels–Alder probe for discovery of natural products containing furan moieties

**DOI:** 10.3762/bjoc.20.88

**Published:** 2024-05-02

**Authors:** Alyssa S Eggly, Namuunzul Otgontseren, Carson B Roberts, Amir Y Alwali, Haylie E Hennigan, Elizabeth I Parkinson

**Affiliations:** 1 Department of Chemistry, Purdue University, West Lafayette, Indiana 47906, United States; 2 Department of Medicinal Chemistry and Molecular Pharmacology, Purdue University, West Lafayette, Indiana 47906, United States

**Keywords:** Diels–Alder reaction, furans, methylenomycin furan hormones, natural products, reactivity-based probes

## Abstract

Natural products (NPs) are fantastic sources of inspiration for novel pharmaceuticals, oftentimes showing unique bioactivity against interesting targets. Specifically, NPs containing furan moieties show activity against a variety of diseases including fungal infections, and cancers. However, it is challenging to discover and isolate these small molecules from cell supernatant. The work described herein showcases the development of a molecular probe that can covalently modify furan moieties via a [4 + 2] Diels–Alder cycloaddition, making them easily identifiable on liquid chromatography–mass spectrometry (LC–MS). The molecular probe, which undergoes this reaction with a variety of furans, was designed with both a UV-tag and a mass tag to enable easy identification. The probe has been tested with a variety of purified furans, including natural products, methylenomycin furan (MMF) hormones, and MMF derivatives. Moreover, the molecular probe has been tested in crude supernatants of various *Streptomyces* strains and enables identification of MMFs.

## Introduction

**Figure 1:**
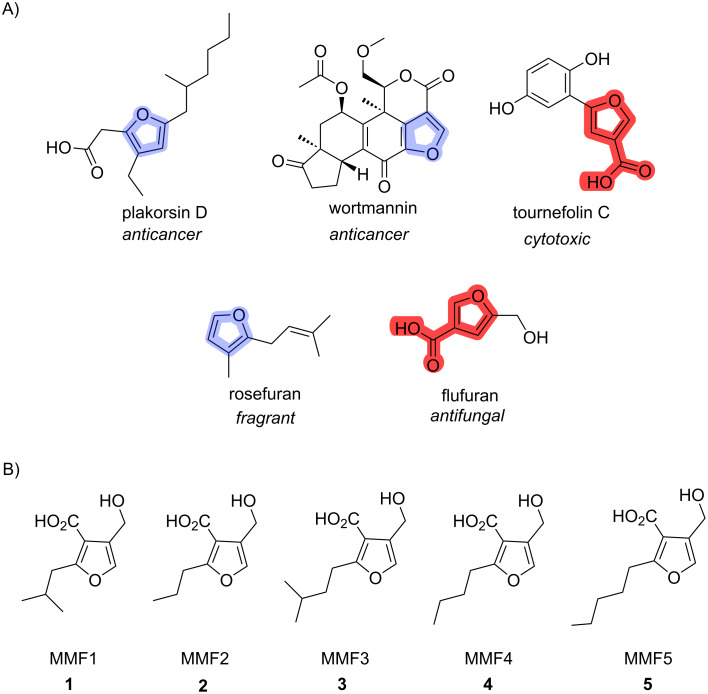
A) Bioactive natural products containing a furan ring (blue) or 3-furoic acid moiety (red): plakorsin D, wortmannin, tournefolin C, rosefuran, and flufuran and their respective biological activities. B) MMF hormones isolated to date.

To date, natural products and their derivatives have served as the foundation of many pharmaceuticals used today. These compounds have activity against various diseases such as cancer, bacterial infections, and fungal infections, as well as other indications [1]. Specifically, natural products containing furan moieties (Figure 1A) are of interest as they have shown promising biological activities.

One well-studied example is the antifungal flufuran, which was isolated from *Aspergillus flavus* [2]. Another example is plakorsin D, an anticancer polyketide natural product which was isolated from a sea sponge, *Plakortis simplex* [3–4]. Wortmannin, which also has a furan, was isolated from *Penicillium wortmannin* and shows anticancer activity via potent inhibition of phosphoinositide 3-kinase [5]. These are just a handful of the bioactive natural products containing furans that exist. Natural products with furan moieties can also be signaling hormones. Methylenomycin furans (MMFs) are naturally occurring secondary metabolites that are produced by *Streptomyces coelicolor*, a soil dwelling bacterium. These molecules are important as they induce the production of the antibiotic methylenomycin A. Specifically, an MMF binds to the TetR family transcriptional repressor (TFTR) resulting in the complex being released from the DNA ultimately allowing for gene transcription and production of enzymatic machinery necessary for the biosynthesis of methylenomycin A [6–7]. To date, there have been five natural MMFs isolated and characterized, with all of the MMFs being isolated from *Streptomyces coelicolor* W75 (Figure 1B). These compounds are 2,3,4-trisubstituted furans, and all five contain a carboxylic acid at the three position and a hydroxymethyl group at the four position. They differ solely based on which alkyl chain is present at the two position [7]. Based on genomic data, it is hypothesized that other secondary metabolites similar to the MMFs exist in other strains of actinobacteria. However, it is time-intensive and challenging to isolate and elucidate novel natural products. Hormones such as MMFs are particularly challenging to isolate because they are often very potent (often active at picomolar concentrations) and thus produced at low levels. To date, MMFs have only been isolated from *Streptomyces coelicolor* W75, with 1 mg of MMF-1 being isolated from approximately 1 L of agar plates (1 mg/L titer or 5 µM). However, this initial isolation was enabled by heterologous expression of their biosynthetic genes from *Streptomyces coelicolor* to produce higher titers of the MMF molecules compared to the native producer [7].

**Figure 2:**
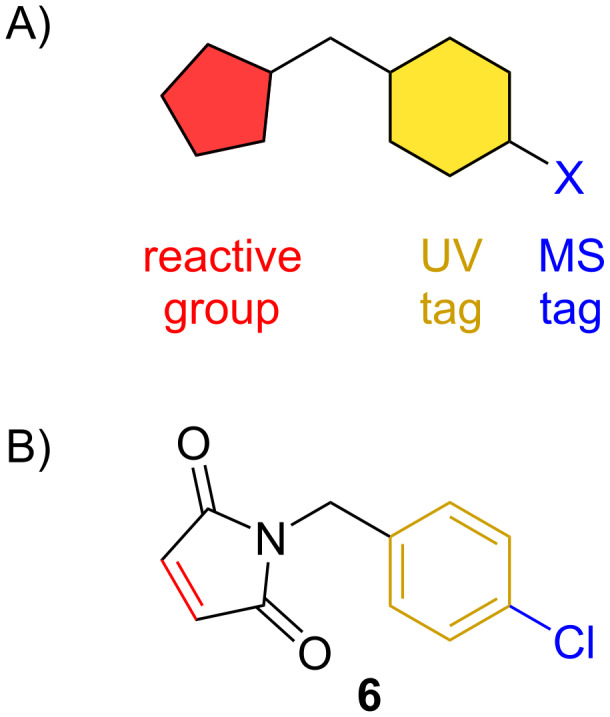
A) A diagram of an example of a molecular probe. B) The maleimide-based probe (**6**) for furans.

Given the challenges of isolating furans generally and the MMFs specifically, we have developed a chemical probe that is capable of covalently binding to natural products containing furan moieties. Molecular probes are molecules that covalently bind to a compound of interest in order to make them easier to identify from the complex milieu of the cell supernatant. There are two main types of molecular probes based on their detection methods: imaging and UV–vis (Figure 2A). An imaging probe contains a mass spectrometry (MS) tag, oftentimes a halogen with a distinct isotopic ratio such as chlorine or bromine. A UV–vis probe contains a UV-tag, such as an aromatic ring, to make the compound UV-active. Previously developed probes containing both MS and UV–vis tags have proven to be successful in cell supernatant in identifying a number of functional groups including epoxides, enones, citrulline, conjugated alkenes, and others [8–16]. One probe has been developed capable of undergoing a Diels–Alder reaction with a peptide containing a terminal furan [17]. However, to date, no one has developed probes that have been demonstrated to work well with natural products containing furans. Therefore, we have developed a probe that can undergo a [4 + 2] Diels–Alder cycloaddition to identify furan moieties within complex cell supernatants.

## Results and Discussion

When designing the reactive group of the furan probe **6**, we chose to focus on a well-known reaction with furans, the Diels–Alder cyclization. While the Diels–Alder cyclization strategy has been previously used in probe development [9,16–17], no one has reported it previously with furans. This is likely because utilizing a furan ring as the diene source can be quite challenging. Furans are aromatic molecules, resulting in these five-membered ring systems being relatively unreactive. Specifically, breaking their aromaticity, which is required for the Diels–Alder reaction to occur, is unfavorable and results in Diels–Alder reactions with furans often being easily reversible [18]. Fortunately, although difficult, it is not impossible to perform a Diels–Alder reaction with furans. While the frontier molecular orbital theory can be applied to this cycloaddition reaction, the thermodynamics must also be taken into consideration. Most Diels–Alder reactions with furans as the diene follow normal-electron-demand with the diene HOMO reacting with the dienophile LUMO due to the oxygen atom in the ring destabilizing the diene HOMO. Thus, when it comes to substituents on a furan ring, it is better to place electron-withdrawing groups in the three and/or five positions of the furan to aid in the natural flow of the electrons [19]. Due to this reactivity, we expected that a Diels–Alder probe would work well for the MMFs as well as other natural products containing electron-withdrawing groups at the three position such as flufuran (**23**). With this in mind, we designed the maleimide-based molecular probe (Figure 2B). Compound **6** was easily synthesized over three steps by adapting an existing route [20] and is inexpensive ($1.80/g of starting material) to make in large quantities. It also contains a chlorine group as its MS tag, which can be easily identified via its 3:1 isotopic ratio when run on a UPLC-MS, and a phenyl ring, which allows it to be UV visible at 214 nm and 254 nm.

**Figure 3:**
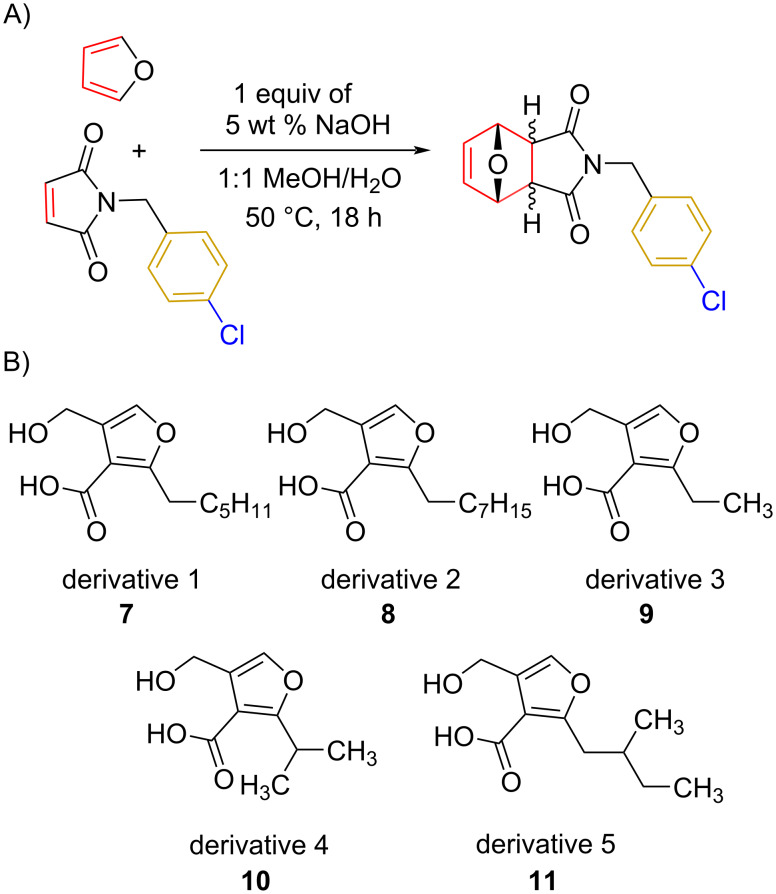
A) General Diels–Alder reaction scheme. B) MMF derivatives explored in this reaction.

Once the probe was synthesized in good yields, the optimization of the Diels–Alder reaction was undertaken. The Diels–Alder reaction (Figure 3A) was optimized utilizing 3-furoic acid (**14**) as it fits the requirement of having an electron-withdrawing group in the three position and was an inexpensive, commercially available option. Initial conditions were based on previously reported work with similar structures [21–22]. We planned to perform the reaction using water as the solvent, but due to solubility challenges with the probe, this was not a viable option. After completing a solvent screen, it was determined that a ratio of 1:1 MeOH/H_2_O should be used as this solubilized the chemical probe most efficiently. Additionally, others have previously noted that polar solvents tend to benefit Diels–Alder reactions between furoic acids and maleimides [19,23]. Next, we looked at how different additives would affect the conversion. Others have observed that sodium hydroxide or salts can have beneficial effects on a Diels–Alder reaction with furans [18–19]. For this reason, we explored both sodium hydroxide and sodium chloride as additives. When 1 equivalent of a 5 percent solution of sodium hydroxide was used, the reaction proceeded well with 78% conversion when monitored by both MS and NMR spectroscopy. However, when sodium chloride was used instead of sodium hydroxide, no conversion was observed. Temperature was also taken into consideration; temperatures above 55 °C were not considered because we aim to use these same conditions in cell extracts to probe for small molecules that may be unstable or volatile at higher temperatures.

To test the chemical probe and determine the substrate scope of the reaction, we tested a small library of furans (Table 1). After each reaction was run, the samples were diluted and run on a UPLC-MS. Cycloadduct formation was verified by the presence of the 3:1 isotopic ratio of the chlorine on the probe and the correct *m*/*z* ratio. The conversion was calculated based on the remaining starting material, which was quantified using a standard curve. In general, very minimal to no side products were observed via MS or NMR, thus validating the use of starting material for quantification.

**Table 1:** Conversion of various synthetic substrates when reacted with the maleimide probe.^a^

Substrate	Structure	Average % conversion^b^ ± standard deviation

furan^c^ (**12**)	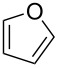	N/A^d^
2-furoic acid (**13**)	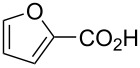	21 ± 1
3-furoic acid (**14**)	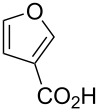	79 ± 8
3-methyl-2-furoic acid (**15**)	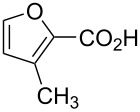	81 ± 6
2-methyl-3-furoic acid (**16**)	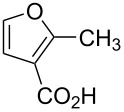	87 ± 8
2-methyl-5-phenyl-3-furoic acid (**17**)	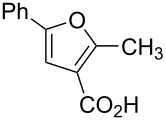	no conversion
furfural (**18**)	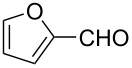	inconclusive
(4-methylfuran-3-yl)methanol (**19**)	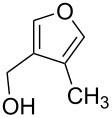	7 ± 7
methyl 3-furan carboxylate (**20**)	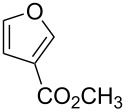	57 ± 8
1*H*-pyrrole (**21**)	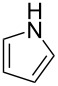	inconclusive
thiophene (**22**)	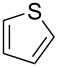	no conversion

^a^Representative LC–MS traces can be found in Figures S1–S12 in Supporting Information File 1. ^b^The average conversion is calculated based on the amount of starting material remaining in the crude reaction. Reactions run in triplicate. ^c^Furan is too volatile to react at 50 °C. ^d^Not available.

From this data, a few generalized conclusions can be drawn. Overall, the substrates with higher conversions were **14** and **16**, which is in line with our hypothesis that having an electron-withdrawing group in the three position will push the reaction forward. With a less electron-withdrawing group, such as **20**, the reaction still proceeds but we see less conversion. When we place an electron-withdrawing group in the two-position, as in **13** or **15**, conversion decreases compared to **14** or **16**, respectively.

In addition to the substrates that did undergo the Diels–Alder [4 + 2] cycloaddition, some substrates were unable to react with our chemical probe. For example, **18** did covalently attach to the probe, but there is no **18** leftover to quantify the amount of conversion. Additionally, the mass spectrum for **18** was more complicated, showing many more side products compared to other tested substrates (see Figure S7 in Supporting Information File 1). We hypothesize this is due to **18** undergoing a reaction in aqueous solutions to generate a geminal diol in place of an aldehyde, as has been previously described [24]. Thiophene (**22**) also did not produce any product, which may be due to the differences in the electronics of the sulfur or the lack of electron-withdrawing groups on the molecule. The phenyl-substituted compound **17** was tested but ultimately showed no conversion as well. Although it does have the carboxylic acid in the three position, the phenyl group is most likely too electron-donating, as well as potentially being too sterically bulky. Finally, pyrrole **21** was tested and ultimately resulted in an inconclusive amount of conversion. Compound **21** does not ionize well on our LC–MS and thus we are unable to quantify the amount of product formed. However, we can see the mass of the product in the total ion chromatogram as well as in the UV trace (Figure S10 in Supporting Information File 1).

Once the initial substrate scope was completed, we moved forward with testing the chemical probe with known natural products. To do this, the five naturally occurring MMFs (**1–5**) were synthesized by following a previous route [22]. In addition to the naturally occurring MMFs, a library of derivatives (Figure 3B) was also tested to determine if changes in the alkyl chain would affect conversion. These derivatives contained shorter side chains, longer side chains, and different branching patterns than the naturally occurring MMFs. Given that other well-studied actinobacteria hormones such as the γ-butyrolactones have varying alkyl chains [25–28], we expect that derivatives of the MMFs with alternative alkyl chains likely exist in other bacterial strains. Lastly, the probe was tested with flufuran (**23**) to show that it can also identify a natural product that is not an MMF.

**Figure 4:**
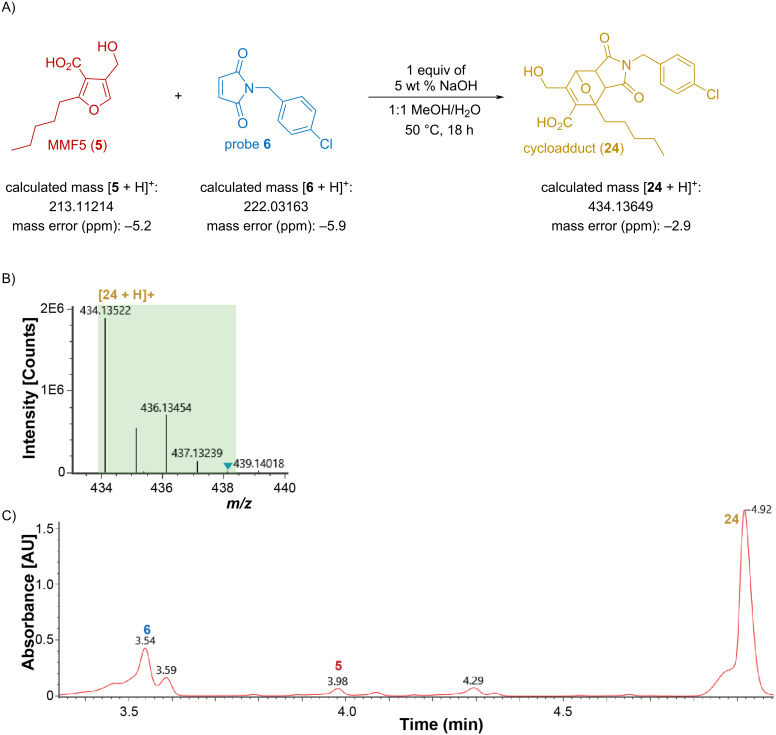
A. Reaction scheme. B. Representative mass spectra of MMF5 cycloadduct **24** with 3:1 isotopic ratio present. C. Representative UV trace of the reaction products; probe **6**, MMF5 (**5**), and MMF5 cycloadduct **24**. All reactions were performed a minimum of three times with similar results being observed in all three replicates.

The maleimide probe was tested with the natural MMF molecules and the library of MMF derivatives in the same fashion as previously described for the synthetic molecules (Table 2). The results from these experiments are very promising (Figure 4), as almost all of the naturally occurring MMF molecules had essentially quantitative conversion at the 18 h timepoint. Compound **1** was the lowest converting of all of the natural MMFS, but it still had an impressive 94% conversion. The five MMF derivatives that were tested were also able to undergo the desired Diels–Alder reaction. Compound **9**, which contains the ethyl group as its side chain showed quantitative conversion. However, when the side chain was increased to an eight-carbon chain shown with **8**, conversion dropped to an average of 86%. This is likely because the bulkier side chain sterically hinders the molecules from aligning in the optimal orientation for the HOMO and LUMO to interact. Similar high yields were observed with **10** and **11**. The maleimide probe also worked well with flufuran (**23**), having a 92% conversion. The slightly lower conversion is most likely due to the loss of the hydroxy group at the four position and is not surprising as it aligns with the results of 3-furoic acid (**14**).

**Table 2:** Conversion of various natural product substrates when reacted with the maleimide probe.^a^

Substrate	Average % conversion^b^ ± standard deviation	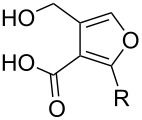 R =

MMF1 (**1**)	94 ± 1	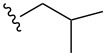
MMF2 (**2**)	98 ± 1	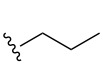
MMF3 (**3**)	98 ± 2	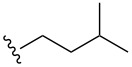
MMF4 (**4**)	98 ± 2	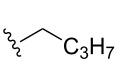
MMF5 (**5**)	99 ± 1	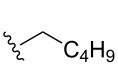
MMF derivative 1 (**7**)	99 ± 1	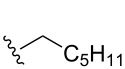
MMF derivative 2 (**8**)	86 ± 4	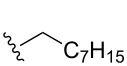
MMF derivative 3 (**9**)	98 ± 1	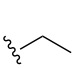
MMF derivative 4 (**10**)	99 ± 7	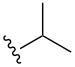
MMF derivative 5 (**11**)	96 ± 1	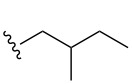
flufuran (**23**)	93 ± 2^c^	N/A^d^

^a^Representative traces can be found in Figures S13–S23 in Supporting Information File 1. ^b^The average conversion is calculated based on the amount of starting material remaining in the crude reaction. The average is based on three replicates. ^c^The average is based on five replicates. ^d^Not applicable.

**Figure 5:**
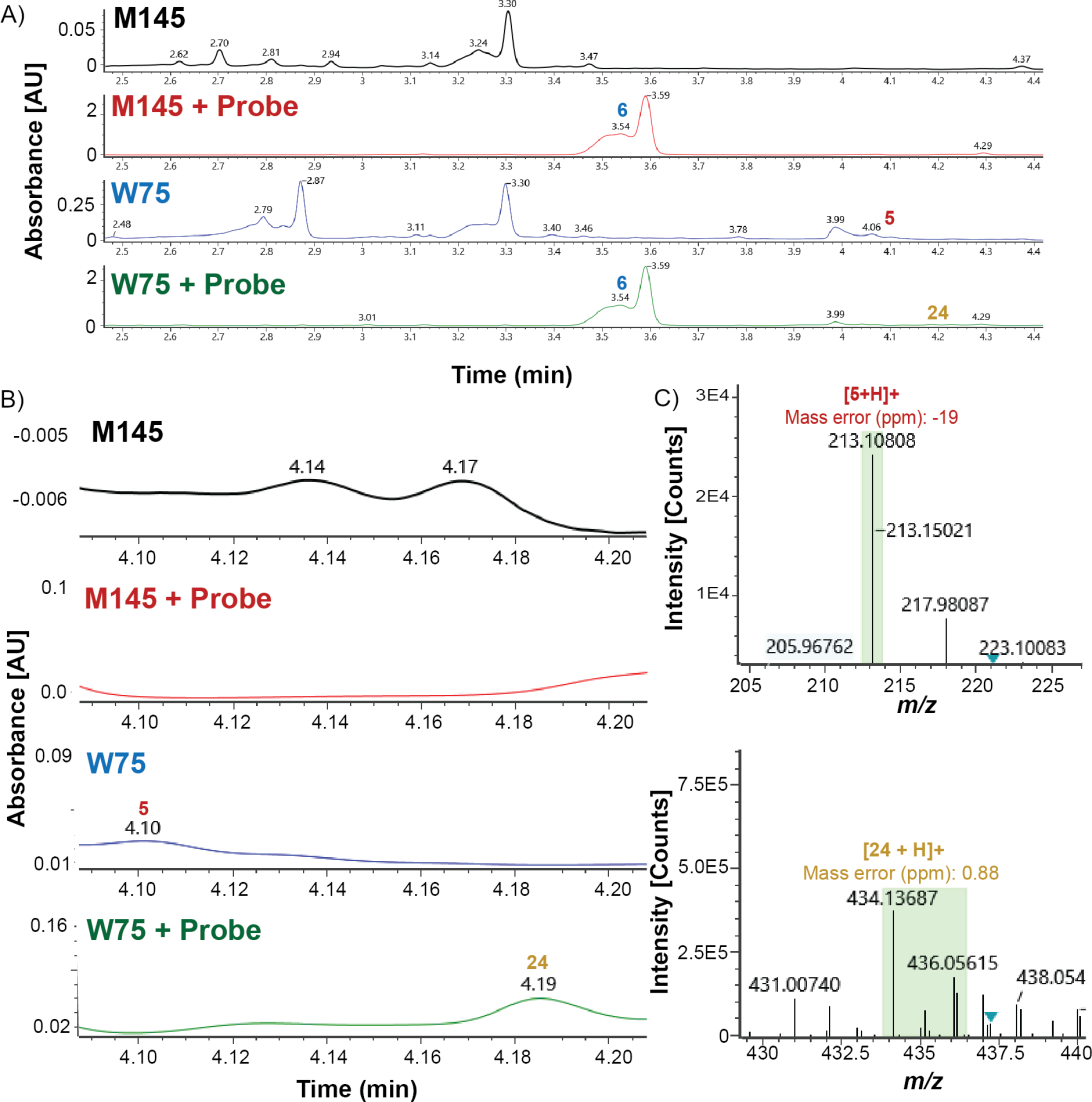
A. The UV trace (254 nm) for M145 control, M145 + probe **6**, W75 control, and W75 + probe **6**. M145 control shows no MMFs and M145 + probe shows only the maleimide probe **6**. W75 control shows MMF5 (**5**) present. W75 + probe shows both MMF5 cycloadduct (**24**) and excess maleimide probe **6**. B. Zoom in of the UV trace (254 nm) for M145 control, M145 + probe, W75 control, and W75 + probe. M145 control shows no MMFs and M145 + probe shows nothing significant in the zoomed in region. W75 control shows MMF5 (**5**) and W75 shows the MMF5 cycloadduct **24**. C. Mass spectra of W75 control and W75 + probe showing mass of MMF5 (**5**) and mass of the MMF5 cycloadduct **24**, respectfully. All samples shown in this figure were run at 25-times the extracted concentration. Representative data from three experiments is shown.

After demonstrating the probe could undergo the Diels–Alder reaction with natural products containing furan moieties, it was tested with crude cell supernatants. Experiments were first performed to determine the limit of detection of the cycloadduct in the cell extracts when run on a UPLC-MS (Figure S23 in Supporting Information File 1). Specifically, a cell supernatant from a strain that does not produce MMFs (*Streptomyces coelicolor* M145) was spiked with varying amounts of compound **14**. The sample was then split, with half being reacted with the maleimide probe and the other half being exposed to the same conditions just lacking the probe. The samples were then used to determine the limit of detection for both the starting material **14**, as well as the cycloadduct product in the complete cell supernatant. The reactions were run at various concentrations of compound **14** ranging from 71 mM to 17 µM. Gratifyingly, the cycloadduct has 260-fold increase in sensitivity compared to **14** (69 µM and 18 mM, respectively), suggesting that the probe will be highly useful for detecting lower concentration furans. The maleimide probe was then tested for its ability to detect the naturally produced MMF compounds (Figure 5). Two strains of *Streptomyces coelicolor* were tested: *S. coelicolor* W75, a strain known to produce the MMF molecules was examined, and *S. coelicolor* M145, a control that does not contain the biosynthetic machinery necessary to produce MMFs but is otherwise genetically identical to *S. coelicolor* W75. Controls containing just the cell supernatant, as well as reactions of the cell supernatant with the maleimide probe, were run on the UPLC-MS after reacting for 18 h. As expected, samples from *S. coelicolor* M145 did not show any MMFs present in either the control sample or the probe-treated sample, even after concentrating the sample 25-fold. Compound **5** and its cycloadduct were both observed in *Streptomyces* W75. Gratifyingly, the cycloadduct was much more easily visualized, with an ion intensity that was approximately 15-fold higher than that of **5** alone. This demonstrates that our chemical probe is able to label naturally occurring furan natural products within cell supernatants and that it can be utilized as a tool to more efficiently uncover furan-containing natural products. However, the ability of the probe to detect furans in cell extracts will depend both on their structures, as described in Table 1, and their titers. The cycloadduct is observed in *S. coelicolor* W75 treated with the probe at both 5-time (≈25 µM) and 25-time (≈125 µM) concentrated samples. This is consistent with our limit of detection of **14**. However, *S. coelicolor* W75 is a heterologous expression strain that produces higher levels of MMFs than expected in native strains. We unfortunately do not know the titers of MMFs in native producing strains. However, if titers are similar to that of γ-butyrolactones and butenolides quorum sensing molecules, we would expect them to be between 80 and 2.5 nM [29–31]. Given these very low concentrations, cultures would need to be greatly concentrated (≈1000 times) to observe the molecules in their native producers. While this is not ideal, it is still significantly better than the concentration needed without probe (≈260,000 times).

## Conclusion

MMFs are important signaling hormones that induce the production of methylenomycin A. Bioinformatics data suggests that there are more natural products containing furan moieties that are waiting to be discovered. However, the discovery of natural products can be tedious because they are often produced at very low levels. We have developed a chemical probe that can alleviate some of these challenges. Our chemical probe contains a UV-tag and MS-tag for easy identification utilizing LC–MS. Additionally, it is capable of covalently attaching to a variety of furan rings via a [4 + 2] Diels–Alder cycloaddition in relatively mild reaction conditions. It reacts with naturally occurring MMFs as well as their derivatives and other natural products such as flufuran. Finally, the maleimide probe has proven that it can identify furan-containing natural products in complex cell supernatants, further demonstrating its potential utility in the discovery of novel furan-containing natural products. Future work will focus on utilizing the probe in additional strains to probe for novel natural products similar to the MMFs.

## Supporting Information

File 1 Experimental materials and methods, supplemental figures, and supplemental spectra.

## Data Availability

The data that supports the findings of this study is available from the corresponding author upon reasonable request.
