# Supplementary material for: A Diels–Alder probe for discovery of natural products containing furan moieties
Source: Beilstein J Org Chem. 2024 May 2;20:1001–10. doi: 10.3762/bjoc.20.88 (PMC11070956; doi:10.3762/bjoc.20.88)
Supplement: File 1 — Experimental materials and methods, supplemental figures, and supplemental spectra. [file Beilstein_J_Org_Chem-20-1001-s001.pdf]

BEILSTEIN JOURNAL OF ORGANIC CHEMISTRY

Supporting Information

for

A Diels–Alder probe for discovery of natural products containing furan moieties

Alyssa S. Eggly, Namuunzul Otgontseren, Carson B. Roberts, Amir Y. Alwali, Haylie E. Hennigan and Elizabeth I. Parkinson

Beilstein J. Org. Chem. **2024,** *20,* 1001–1010. doi:10.3762/bjoc.20.88

Experimental materials and methods, supplemental figures, and supplemental spectra

License and Terms: This is a supporting information file under the terms of the Creative Commons Attribution License (https://creativecommons.org/licenses/by/4.0). Please note that the reuse, redistribution and reproduction in particular requires that the author(s) and source are credited and that individual graphics may be subject to special legal provisions.
The license is subject to the *Beilstein Journal of Organic Chemistry* terms and conditions: (https://www.beilstein-journals.org/bjoc/terms)

Table of contents

Methods........ S2

General synthetic methods for MMFs........ S2

Naturally occurring MMF molecules S5

MMF derivatives S7

Maleimide probe synthesis........ S13

Maleimide probe Diels–Alder reaction S14

Maleimide probe Diels–Alder reaction for small scale substrate scope S15

Cell supernatant preparation........ S16

Maleimide probe Diels–Alder reaction in cell extract........ S17

Supporting figures S18

References S30

NMR spectra S31

Methods

General synthetic methods for MMFs

Acylated meldrum's acid & methanolysis

Method A

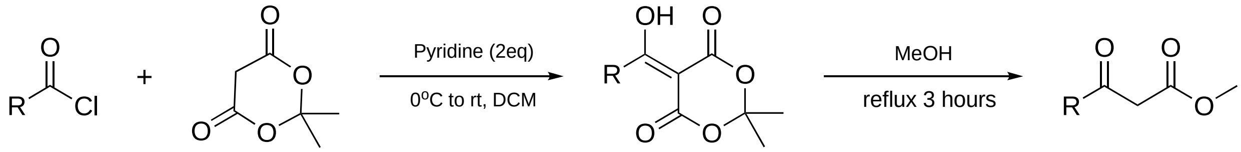

In a flame-dried round-bottomed flask equipped with a stir bar, meldrum's acid (1 equiv) was dissolved in dry DCM (0.08 M). The solution was cooled to 0 °C in an ice bath and pyridine (2 equiv) was added. The solution was allowed to stir for 10 minutes. Acyl chloride (1 equiv) was added dropwise. The solution was allowed to stir for 1 hour at 0 °C and then overnight at room temperature. The reaction was quenched with 1 M HCl and extracted with CH_2Cl_2 (50 mL) three times. It was then washed with 1 M HCl and brine, dried over Na_2SO_4, and concentrated.
The crude oil was then dissolved in CH_3OH (0.5 M) and brought to reflux for 4 hours- overnight. The reaction was cooled to room temperature and concentrated. The crude oil was purified via column chromatography (7:1 hexanes/ethyl acetate) to afford product. Product is a clear, colorless oil.

Method B

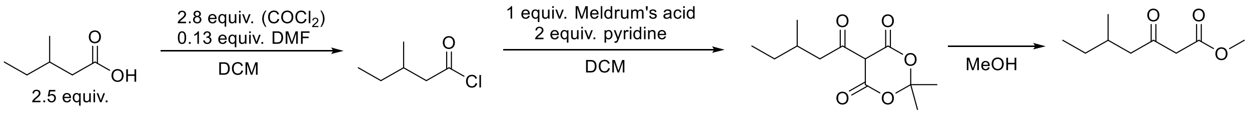

In a flame-dried round-bottomed flask equipped with a stir bar, the carboxylic acid (2.5 equiv) was dissolved in dry DCM. The reaction was cooled in an ice bath to 0 °C and DMF (0.13 equiv) was

added. The oxalyl chloride (2.8 equiv) was added dropwise and the reaction was left to stir for two hours. It was concentrated under N_2.

In a separate flame-dried round-bottomed flask equipped with a stir bar, meldrum's acid (1 equiv) was dissolved in dry CH_2Cl_2 (0.08 M). The solution was cooled to 0 °C in an ice bath and pyridine (2 equiv) was added. The solution was allowed to stir for 10 minutes. The prepared acyl chloride was added dropwise. The solution was allowed to stir for 1 hour at 0 °C and then overnight at room temperature. The reaction was quenched with 1 M HCl and extracted with CH_2Cl_2 (50 mL) three times. It was then washed with 1 M HCl and brine, dried over Na_2SO_4, and concentrated.

The crude oil was then dissolved in CH_3OH (0.5 M) and brought to reflux for 4 hours- overnight. The reaction was cooled to room temperature and concentrated. Crude oil was purified via column chromatography (7:1 hexanes/ethyl acetate) to afford product. Product is a clear, colorless oil.

Furan ring cyclization

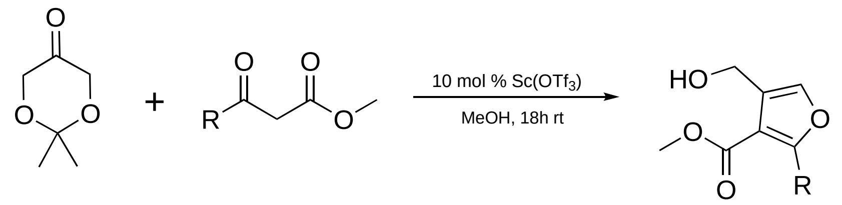

Method A

In a vial equipped with a stir bar, the β-ketomethylester intermediate was dissolved in CH_3OH (0.5 M) and 2,2 dimethyl-1,3-dioxane-5-one (1.2 equiv) was added. Then, $Sc(OTf_3)$ (10 mol %) was added. The solution stirred for 18 hours at room temperature. It was then concentrated and purified via column chromatography (7:2 hexanes/ethyl acetate) to afford the product. The product is a clear, colorless oil.

Method B

In a vial equipped with a stir bar, the β-ketomethylester intermediate was dissolved in CH_3OH (0.5 M) and 2,2 dimethyl-1,3-dioxane-5-one (1.2 equiv) was added. Then, $Sc(OTf_3)$ (10 mol%) was added. The solution stirred for 6 hours at room temperature. Another equivalence of $Sc(OTf_3)$ (10 mol %) was added and the reaction was allowed to stir for 12 additional hours at room temperature. It was then concentrated and purified via column chromatography (7:2 hexanes/ethyl acetate) to afford the product. The product is a clear, colorless oil.

Hydrolysis to afford MMF

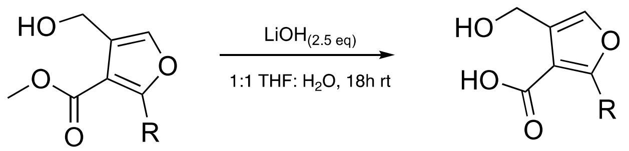

Method A

In a vial, the carboxylate intermediate was dissolved in 1:1 THF/H_2O (0.5 M). LiOH (2.5 equiv) was added and the reaction was stirred for 18 hours at room temperature. The reaction was then concentrated to remove the THF. The aqueous layer was acidified with 10% HCl to a pH of 2–3. The reaction mixture was then extracted with ethyl acetate three times. The organic layers were collected, dried over Na_2SO_4, and concentrated to afford the product. The product is a white, waxy solid.

Method B

In a vial, the methylester furan intermediate was dissolved in 1:1 THF/H_2O (0.5 M). LiOH (2.5 equiv) was added and the reaction was stirred for 6 hours at room temperature. Then, an additional equivalence of LiOH (1 equiv) was added and the reaction was allowed to stir for an additional 12 hours at room temperature. The reaction was then concentrated to remove the THF. The aqueous layer was acidified with 10% HCl to a pH of 2–3. The reaction mixture was then extracted with ethyl acetate three times. The organic layers were collected, dried over Na_2SO_4, and concentrated to afford the product. The product is a white, waxy solid.

Naturally occurring MMF molecules

*All protocols followed from [1], all NMRs coincide with previously reported spectra [2, 3].

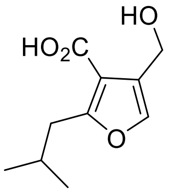

MMF1 (**1**)

4-(Hydroxymethyl)-2-isobutylfuran-3-carboxylic acid

^{1}HNMR (400MHz, $CDCl_3$): δ 7.28 (s, 1H), 4.61 (s, 2H), 2.90 (d, *J* = 7.1 Hz, 2H), 2.10 (hept, *J* = 6.4 Hz, 1H), 0.95 (d, *J* = 6.6 Hz, 6H).

^{13}CNMR (100MHz, $CDCl_3$): ^{13}C NMR (101 MHz, $CDCl_3$) δ 170.09, 166.18, 138.74, 125.32, 112.04, 55.55, 36.75, 28.19, 22.29.

HRMS: m/z calculated for $[C_{10}H_{14}O_4\text{-}H_2O+H]^+$: 181.08592, observed 181.0861 (error: 0.99 ppm)

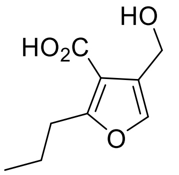

MMF2 (**2**)

4-(Hydroxymethyl)-2-propylfuran-3-carboxylic acid

^{1}HNMR (400MHz, $CDCl_3$): δ 7.27 (s, 1H), 4.60 (s, 2H), 2.97 (t, *J* = 7.6 Hz, 2H), 1.70 (h, *J* = 7.0 Hz, 2H), 0.95 (t, *J* = 7.0 Hz, 3H).

^{13}CNMR (100MHz, $CDCl_3$): δ 169.92, 166.68, 138.64, 125.30, 111.53, 55.52, 29.97, 21.19, 13.63.

HRMS: m/z calculated for $[C_9H_{12}O_4\text{-}H_2O+H]^+$: 167.07027, observed 167.0703 (error: 0.18 ppm)

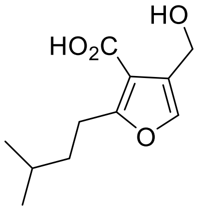

MMF3 (**3**)

4-(Hydroxymethyl)-2-isopentylfuran-3-carboxylic acid

^{1}HNMR (400MHz, $CDCl_3$): δ 7.28 (s, 1H), 4.60 (s, 2H), 3.02 (t, *J* = 7.6 Hz, 2H), 1.65 – 1.50 (m, 3H), 0.94 (d, *J* = 6.1 Hz, 6H).

^{13}CNMR (100MHz, $CDCl_3$): δ 169.87, 167.17, 138.58, 125.39, 111.18, 55.54, 36.63, 27.66, 26.20, 22.19.

HRMS: m/z calculated for $[C_{11}H_{16}O_4\text{-}H_2O+H]^+$: 195.10157, observed 195.1020 (error: 2.20 ppm)

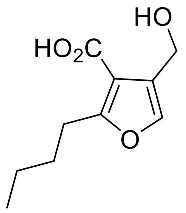

MMF4 (**4**)

2-Butyl-4-(hydroxymethyl)furan-3-carboxylic acid

^{1}HNMR (400MHz, $CDCl_3$): δ 7.27 (s, 1H), 4.60 (s, 2H), 3.01 (d, *J* = 7.4 Hz, 2H), 1.72 – 1.62 (m, 2H), 1.42 – 1.32 (m, 2H), 0.93 (d, *J* = 7.3 Hz, 3H).

^{13}CNMR (100MHz, $CDCl_3$): ^{13}C NMR (101 MHz, $CDCl_3$) δ 170.00, 167.00, 138.61, 125.37, 111.32, 55.54, 29.86, 27.86, 22.21, 13.64.

HRMS: m/z calculated for $[C_{10}H_{14}O_4\text{-}H_2O+H]^+$: 181.08592, observed 181.0862 (error: 1.54 ppm)

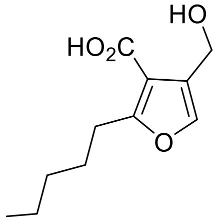

MMF5 (**5**)

4-(Hydroxymethyl)-2-pentylfuran-3-carboxylic acid

^{1}HNMR (400MHz, $CDCl_3$): δ 7.27 (s, 1H), 4.60 (s, 2H), 3.00 (t, *J* = 7.8 Hz, 2H), 1.75 – 1.63 (m, 2H), 1.37 – 1.29 (m, 4H), 0.89 (t, *J* = 6.5 Hz, 3H).

^{13}CNMR (100MHz, $CDCl_3$): δ 169.84, 138.62, 125.41, 111.23, 55.56, 31.26, 28.11, 27.46, 22.24, 13.82.

HRMS: m/z calculated for $[C_{11}H_{16}O_4\text{-}H_2O+H]^+$: 195.10157, observed 195.1020 (error: 2.20 ppm)

MMF Derivatives

***All protocols adapted or followed from [1].

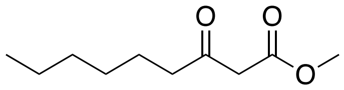

Methyl 3-oxononanoate

Prepared by Method A

Yield= 43%, 108mg

Rf: 0.53

^{1}HNMR (400MHz, CDCl$_3$): δ 3.67 (s, 3H), 3.39 (s, 2H), 2.47 (dd, *J* = 356.4, 7.4 Hz, 2H), 1.52 (p, *J* = 7.2 Hz, 2H), 1.30 – 1.15 (m, 7H), 0.81 (t, *J* = 6.6 Hz, 4H)

^{13}CNMR (100MHz, CDCl$_3$): δ 202.67, 167.55, 52.07, 48.80, 42.86, 31.36, 28.54, 28.49, 23.24, 22.28, 13.80.

HRMS: m/z calculated for $[C_{10}H_{18}O_3+H]^+$: 187.13287, observed 187.13281 (error: -0.32 ppm)

Spectra are consistent with previously published data [1].

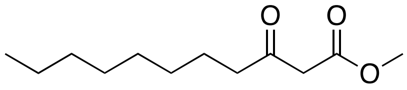

Methyl 3-oxoundecanoate

Prepared by Method A

Yield=39%, 118mg

Rf: 0.44

^{1}HNMR (400MHz, CDCl$_3$): δ 3.71 (s, 3H), 3.42 (s, 2H), 2.50 (t, *J* = 7.3 Hz, 2H), 1.57 (p, *J* = 6.7 Hz, 2H), 1.33 – 1.18 (m, 10H), 0.85 (t, *J* = 6.5 Hz, 3H).

^{13}CNMR (100MHz, CDCl$_3$): δ 202.70, 167.57, 52.16, 48.88, 42.95, 31.67, 29.17, 29.01, 28.96, 28.88, 23.35, 22.50, 13.94.

HRMS: m/z calculated for $[C_{12}H_{22}O_3+H]^+$: 215.16417, observed 215.1644 (error: 1.06 ppm)

Spectra are consistent with previously published data [4].

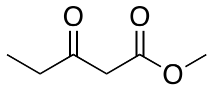

Methyl 3-oxopentanoate

Prepared by Method A

Yield= 39%, 137mg

Rf: 0.31

^{1}HNMR (400MHz, $CDCl_3$): δ 3.69 (s, 3H), 3.42 (s, 2H), 2.51 (q, *J* = 7.2 Hz, 2H), 1.05 (t, *J* = 7.5 Hz, 3H).

^{13}CNMR (100MHz, $CDCl_3$): δ 203.06, 167.59, 52.14, 48.55, 36.17, 7.36.

HRMS: m/z calculated for $[C_6H_{10}O_3+H]^+$: 131.07027, observed 131.0701 (error: -1.30 ppm)

Spectra are consistent with previously published data [5].

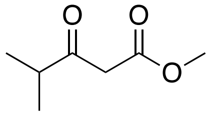

Methyl 4-methyl-3-oxopentanoate

Prepared by Method A

Yield= 31%, 104mg

Rf: 0.37

^{1}HNMR (400MHz, $CDCl_3$): ^{1}H NMR (400 MHz, $CDCl_3$) δ 3.68 (s, 3H), 3.47 (s, 2H), 2.70 (p, *J* = 7.1 Hz, 1H), 1.16 (d, *J* = 7.1 Hz, 2H), 1.09 (d, *J* = 7.0 Hz, 7H).

^{13}CNMR (100MHz, $CDCl_3$): δ 206.32, 167.73, 52.12, 46.66, 41.08, 17.73.

HRMS: m/z calculated for $[C_7H_{12}O_3+H]^+$: 145.08592, observed 145.0859 (error: -0.14 ppm)

Spectra are consistent with previously published data [6].

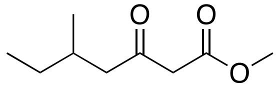

Methyl 5-methyl-3-oxoheptanoate

Prepared by Method B

Yield= 17%, 58mg

Rf: 0.57

^{1}HNMR (400MHz, $CDCl_3$): δ 3.74 (s, 4H), 3.46 (s, 2H), 2.54 (d, *J* = 5.7 Hz, 1H), 2.34 (d, *J* = 7.9 Hz, 1H), 1.97 (p, *J* = 8.0 Hz, 1H), 1.45 – 1.28 (m, 1H), 1.24 – 1.14 (m, 1H), 0.88 (q, *J* = 6.4 Hz, 8H).

^{13}CNMR (100MHz, $CDCl_3$): δ 202.46, 178.22, 167.52, 89.65, 52.14, 50.88, 49.89, 49.33, 42.20, 32.33, 30.42, 29.20, 29.14, 19.11, 18.82, 11.13.

HRMS: m/z calculated for $[C_9H_{16}O_3+H]^+$: 173.11722, observed 173.1168 (error: -2.43 ppm)

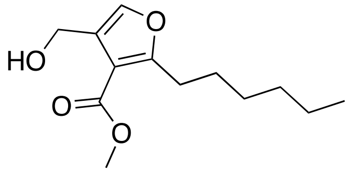

Methyl 2-hexyl-4-(hydroxymethyl)furan-3-carboxylate

Prepared with Method B

Yield= 52%, 66mg

Rf: 0.19

^{1}HNMR (400MHz, $CDCl_3$): δ 7.23 (s, 1H), 4.54 (d, J = 1.0 Hz, 2H), 3.87 (s, 3H), 2.94 (t, J = 7.6 Hz, 2H), 1.64 (p, J = 6.1 Hz, 2H), 1.36 – 1.22 (m, 7H), 0.88 (t, J = 6.6 Hz, 3H).

^{13}CNMR (100MHz, $CDCl_3$): δ 165.64, 164.86, 138.21, 125.69, 111.92, 55.64, 51.60, 31.33, 28.74, 28.11, 27.81, 22.39, 13.93.

HRMS: m/z calculated for $[C_{13}H_{20}O_4-H_2O+H]^+$: 223.13287, observed 223.1328 (error: -0.31 ppm)

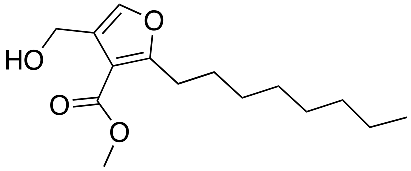

Methyl 4-(hydroxymethyl)-2-octylfuran-3-carboxylate

Prepared with Method B

Yield= 51%, 66mg

Rf: 0.10

^{1}HNMR (400MHz, $CDCl_3$): δ 7.26 (s, 1H), 4.58 (d, J = 6.8 Hz, 2H), 3.89 (s, 3H), 2.94 (t, J = 7.1 Hz, 2H), 1.66 (p, J = 6.8 Hz, 2H), 1.37 – 1.23 (m, 11H), 0.90 (t, J = 6.8 Hz, 3H).

^{13}CNMR (100MHz, $CDCl_3$): δ 165.73, 164.97, 138.31, 125.81, 112.03, 55.76, 51.69, 31.83, 29.21, 29.19, 29.15, 27.93, 22.64, 14.08.

HRMS: m/z calculated for $[C_{15}H_{24}O_4\text{-}H_2O+H]^+$: 251.16417, observed 251.1644 (error: 0.92 ppm)

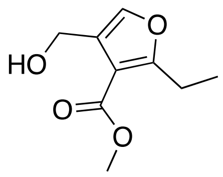

Methyl 2-ethyl-4-(hydroxymethyl)furan-3-carboxylate

Prepared with Method A

Yield= 40%, 76mg

Rf: 0.11

^{1}HNMR (400MHz, CDCl$_3$): δ 7.21 (t, *J* = 0.7 Hz, 1H), 4.52 (s, 2H), 3.84 (s, 3H), 2.92 (q, *J* = 7.7 Hz, 2H), 1.20 (t, *J* = 7.5 Hz, 3H).

^{13}CNMR (100MHz, CDCl$_3$): δ 165.52, 138.17, 125.78, 111.41, 55.63, 51.58, 21.68, 11.99.

HRMS: m/z calculated for $[C_9H_{12}O_4\text{-}H_2O+H]^+$: 167.07027, observed 167.0703 (error: 0.18 ppm)

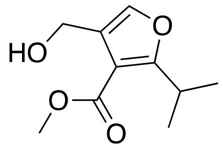

Methyl 4-(hydroxymethyl)-2-isopropylfuran-3-carboxylate

Prepared with Method A

Yield= 27%, 36mg

Rf: 0.22 (7:2, Hex: EtOAc)

^{1}HNMR (400MHz, CDCl$_3$): δ 7.23 (t, *J* = 0.7 Hz, 1H), 4.54 (d, *J* = 0.9 Hz, 2H), 3.86 (s, 3H), 3.67 (h, *J* = 7.0 Hz, 1H), 1.25 (d, *J* = 6.7 Hz, 7H).

^{13}CNMR (100MHz, CDCl$_3$): δ 168.59, 165.62, 138.12, 125.54, 110.48, 55.65, 51.63, 27.58, 20.59.

HRMS: m/z calculated for $[C_{10}H_{14}O_4\text{-}H_2O+H]^+$: 181.08592, observed 181.0861 (error: 0.99 ppm)

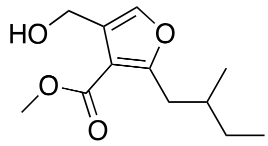

Methyl 4-(hydroxymethyl)-2-(2-methylbutyl)furan-3-carboxylate

Yield= 42%, 30mg

Rf: 0.34 (6:2, Hex: EtOAc)

^{1}HNMR (400MHz, $CDCl_3$): δ 7.24 (s, 1H), 4.55 (s, 2H), 3.86 (s, 3H), 2.91 (dd, *J* = 7.8, 6.4 Hz, 1H), 2.72 (dd, *J* = 8.2, 7.4 Hz, 1H), 1.80 (h, *J* = 6.8 Hz, 1H), 1.44 – 1.15 (m, 2H), 0.87 (q, *J* = 8.2 Hz, 6H).

^{13}CNMR (100MHz, $CDCl_3$): δ 165.69, 164.26, 138.32, 125.65, 112.66, 55.66, 51.57, 34.93, 34.62, 29.26, 19.08, 11.27.

HRMS: m/z calculated for $[C_{12}H_{18}O_4+H]^+$: 227.12779, observed 227.1270 (error: -3.48 ppm)

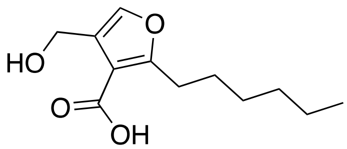

2-Hexyl-4-(hydroxymethyl)furan-3-carboxylic acid (**7**)

Prepared with Method A

Yield= 92%, 50mg

Rf: 0.31

^{1}HNMR (400MHz, $CDCl_3$): δ 7.26 (s, 1H), 4.60 (s, 2H), 2.99 (t, *J* = 7.5 Hz, 2H), 1.67 (p, *J* = 7.8 Hz, 2H), 1.40 – 1.22 (m, 6H), 0.88 (t, *J* = 6.7 Hz, 3H).

^{13}CNMR (100MHz, $CDCl_3$): δ 170.01, 166.94, 138.59, 125.34, 111.37, 55.53, 31.36, 28.77, 28.12, 27.71, 22.39, 13.92.

HRMS: m/z calculated for $[C_{10}H_{14}O_4\text{-}H_2O+H]^+$: 209.11722, observed 209.1174 (error: 0.86 ppm)

FTIR: 1662 cm^{-1}, 2889 cm^{-1}, 2954 cm^{-1}, 3334 cm^{-1}

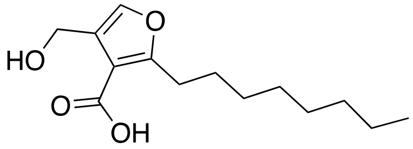

4-(Hydroxymethyl)-2-octylfuran-3-carboxylic acid (**8**)

Prepared with Method B

Yield=80, 49mg

Rf: 0.34

^{1}HNMR (400MHz, CDCl$_3$): δ 7.27 (s, 1H), 4.60 (s, 2H), 3.01 (t, *J* = 7.8 Hz, 2H), 1.68 (p, *J* = 6.6 Hz, 2H), 1.38 – 1.15 (m, 10H), 0.88 (t, *J* = 6.3 Hz, 3H).
^{13}CNMR (100MHz, CDCl$_3$): δ 169.91, 167.02, 138.60, 125.38, 111.29, 55.55, 31.72, 29.13, 29.04, 28.14, 27.76, 22.53, 13.98.
HRMS: m/z calculated for $[C_{14}H_{22}O_4\text{-}H_2O+H]^+$: 237.14852, observed 237.1488 (error: 1.18 ppm)
FTIR: 1676 cm^{-1}, 2862 cm^{-1}, 2917 cm^{-1}, 3390 cm^{-1}

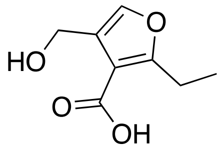

2-Ethyl-4-(hydroxymethyl)furan-3-carboxylic acid (**9**)
Prepared with Method A
Yield= 81%, 55mg
Rf: 0.14
^{1}HNMR (400MHz, CDCl$_3$): δ 7.27 (s, 1H), 4.61 (s, 2H), 3.02 (q, *J* = 7.4 Hz, 2H), 1.25 (t, *J* = 7.5 Hz, 3H).
^{13}CNMR (100MHz, CDCl$_3$): δ 169.90, 167.64, 138.59, 125.34, 110.95, 55.50, 21.71, 11.92.
HRMS: m/z calculated for $[C_8H_{10}O_4\text{-}H_2O+H]^+$: 153.05462, observed 153.0546 (error: -0.13 ppm)
FTIR: 1696cm^{-1}, 2917cm^{-1}, 2977cm^{-1}, 3376cm^{-1}

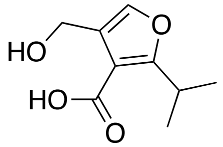

4-(Hydroxymethyl)-2-isopropylfuran-3-carboxylic acid (**10**)
Yield= 64%, 18mg
Rf: 0.26
^{1}HNMR (400MHz, CDCl$_3$): δ 7.28 (s, 1H), 4.60 (s, 2H), 3.79 (p, *J* = 7.4 Hz, 1H), 1.29 (d, *J* = 7.5 Hz, 7H).
^{13}CNMR (100MHz, CDCl$_3$): δ 170.82, 170.06, 138.53, 125.23, 109.89, 55.55, 27.58, 20.52.
HRMS: m/z calculated for $[C_9H_{12}O_4\text{-}H_2O+H]^+$: 167.07027, observed 167.0704 (error: 0.77 ppm)

FTIR: 2967.9 cm^{-1}, 2848.4 cm^{-1}, 1662.3 cm^{-1}, 1517.7 cm^{-1}, 1083.8 cm^{-1}

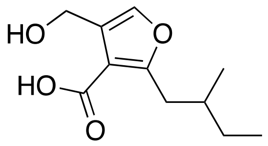

4-(Hydroxymethyl)-2-(2-methylbutyl)furan-3-carboxylic acid (**11**)

Yield= 88%, 25mg

Rf: 0.30

^{1}HNMR (400MHz, CDCl$_3$): δ 7.28 (s, 2H), 4.60 (s, 2H), 2.98 (dd, *J* = 6.5, 6.5 Hz, 1H), 2.86 (dd, *J* = 9.3, 8.4 Hz, 1H), 1.88 (h, *J* = 7.0 Hz, 1H), 1.44 (hept, *J* = 6.9 Hz, 1H), 1.23 (h, *J* = 7.5 Hz, 2H), 0.90 (t, *J* = 7.4 Hz, 6H).

^{13}CNMR (100MHz, CDCl$_3$): δ 170.07, 166.37, 138.72, 125.33, 112.07, 55.56, 34.93, 34.49, 29.24, 19.00, 11.25.

HRMS: m/z calculated for $[C_{11}H_{16}O_4+H]^+$: 213.11214, observed 213.1116 (error: -2.53 ppm)

FTIR: 2912.0 cm^{-1}, 1672.0 cm^{-1}, 1427.1 cm^{-1}, 1099.2 cm^{-1}

Maleimide probe synthesis

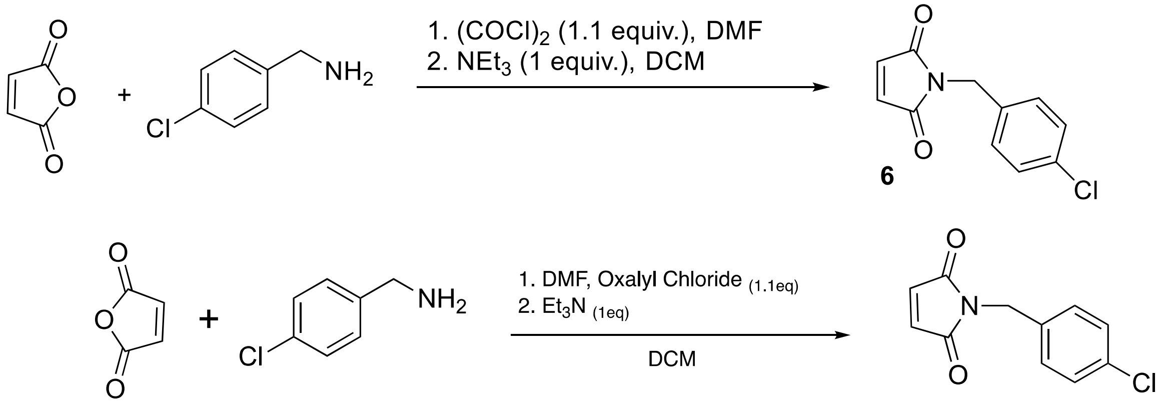

*Protocol followed from [7] with a few adaptations. The adaptations are as follows:

After addition of the oxalyl chloride (1.1 equiv), the reaction was stirred for two hours at 0 °C. It was then concentrated and further dried under high vacuum for 30 minutes. The crude mixture was

then resuspended in dry DCM and cooled to 0 °C. Et_3N (1.5 equiv) was added dropwise and the reaction was allowed to stir overnight at room temperature.

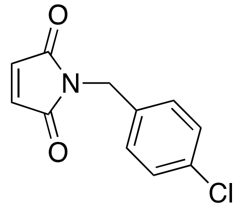

1-(4-Chlorobenzyl)-1H-pyrrole-2,5-dione

Yield= 67%, 1.48g

Rf: 0.51 (2:1 Hex : EtOAc)

^{1}HNMR (400MHz, $CDCl_3$): δ 7.27 (s, 4H), 6.72 (s, 2H), 4.62 (s, 2H).

^{13}CNMR (100MHz, $CDCl_3$): δ 170.15, 134.51, 134.15, 133.74, 129.80, 128.77, 40.65.

HRMS: m/z calculated for $[C_{11}H_8ClNO_2+H]^+$: 222.03163, observed 222.0318 (error: 0.77 ppm)

Maleimide probe Diels–Alder reaction

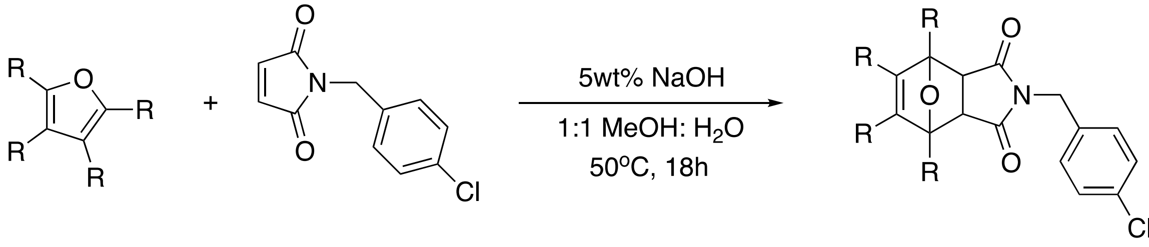

In a round-bottomed flask equipped with a stir bar, furan (0.45 mmol) and maleimide probe (1.5 equiv, 0.67 mmol) were dissolved in 1:1 methanol: RO water (0.1 M). Then, NaOH (0.36 mL of 5 wt% NaOH, 1.0 equiv) was added. The reaction vessel was sonicated for 5 minutes until the solution was cloudy. The reaction was heated to 50 °C and left to stir overnight. The reaction mixture was extracted with dichloromethane (3 × 15mL) to remove excess maleimide probe. The aqueous layer was then acidified with 1 M HCl to afford a pH of 3. This was extracted with ethyl acetate (3×) and the organic layer was dried over Na_2SO_4, filtered, and concentrated. Crude solid was purified via column chromatography (4:1 ethyl acetate/hexane) to afford product. Product is a white solid.

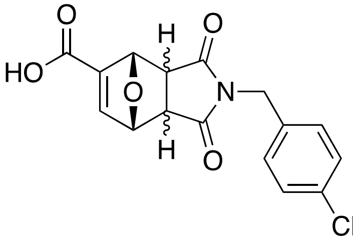

(4S,7S)-2-(4-Chlorobenzyl)-1,3-dioxo-2,3,3a,4,7,7a-hexahydro-1H-4,7-epoxyisoindole-5-carboxylic acid

Yield= 35%, 45mg

Rf: 0.16, (3:1 Ethyl Acetate: Hexanes)

^{1}HNMR (400MHz, $CDCl_3$): δ 7.32 (d, *J* = 1.9 Hz, 1H), 7.29 – 7.22 (m, 5H), 5.50 (s, 1H), 5.41 (d, *J* = 1.2 Hz, 1H), 4.61 (s, 2H), 3.01 (d, *J* = 6.7 Hz, 1H), 2.98 (d, *J* = 6.5 Hz, 1H).

^{13}CNMR (100MHz, $CDCl_3$): δ 174.71, 165.57, 146.88, 142.35, 133.82, 133.52, 129.64, 128.78, 82.23, 80.37, 47.24, 46.98, 41.98.

HRMS: m/z calculated for $[C_{16}H_{12}ClNO_5+H]^+$: 334.04768, observed 334.0468 (error: -2.63 ppm)

Maleimide probe Diels–Alder reaction for small scale substrate scope

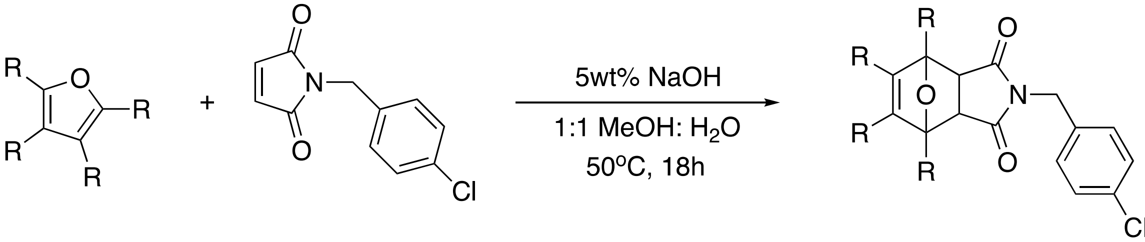

In a vial equipped with a stir bar, furan (1 equiv) and maleimide probe (1.5 equiv) were dissolved in 1:1 methanol:Ro water (0.1 M). Then, 5 wt % NaOH (1.0 equiv) was added. The reaction vessel was sonicated for 5 minutes until the solution was cloudy. The reaction was heated to 50 °C and left to stir for 18 hours. The aqueous reaction mixture was acidified with 1 M HCl until cloudy. This was extracted with ethyl acetate (3 × 2mL) and the organic layer was concentrated via rotary evaporation. The crude oil was then diluted in 5.4 mL of MeOH and run on the UPLC-MS.

UPLC was utilized to determine the amount of conversion based on left over starting material by monitoring the absorbance at 254 nm. The UPLC analysis was carried out on an ACQUITY UPLC

BEH C18 Column, 130Å, 1.7 µm, 2.1 mm X 50mm (Waters) column. The trace was obtained by using mobile phases of H_2O + 0.1% formic acid (A) and acetonitrile + 0.1% formic acid (B). Samples were eluted using a gradient with mobile phase B ranging from 5% to 60% over 8.5 minutes at a flow rate of 0.5 mL/min. The column was equilibrated with 5% mobile phase B for 1 minute before and after the gradient.

Cell supernatant preparation

A single colony of either *Streptomyces coelicolor* M145 or *Streptomyces coelicolor* W75 was used to inoculate 5 mL of ATCC 175 liquid (per L, 10 g dextrose, 20 g soluble starch, 5 g yeast extract, 5 g N-Z Amine Type A, 1 g $CaCO_3$, adjusted to pH 7.3 before autoclaving) in a 20 × 150 mm^2 test tube and grown on an angled roller drum at 30 °C for 4 days. As per methods in [3], 0.5 mL of the seed culture was then used to inoculate each plate of Alanine Minimal Media (AlaMM, per L, 30 mM L-alanine (2.67 g), 5 mM potassium phosphate (0.871 g), 5 mM magnesium sulfate hydrate (1.23 g), 10 g/L glycerol (7.93 mL) and 15 g/L agar). These cultures were spread with sterile glass beads and incubated at 30 °C for 5 days. Each strain was plated on 10 plates. After 5 days, the plates were cut with a razor blade and soaked in 500 mL of ethyl acetate (EtOAc) for around 24 hours. The next day, the EtOAc extract was concentrated via rotary evaporation. The final yields of EtOAc extractions were 37 mg for *S. coelicolor* M145 and 81 mg for *S. coelicolor* W75. These extracts were stored at −80 °C until used.

Limit of detection in cell extracts

The initial volume of the *S. coelicolor* M145 growth prior to extraction was the volume for a 1× concentration. Volumes of solvent added were then reduced appropriately to obtain other concentrations (*e.g.* 2×, 5×, or 25×). Serial dilutions of 3-furoic acid were made in methanol to achieve reactions of 27 µmol, 6.8 µmol, 1.7 µmol, 420 nmol, 105 nmol, 26 nmol, 6.6 nmol of 3-furoic acid in 125 µL of methanol.

In a 4 mL vial equipped with a stir bar, 3-furoic acid dissolved in methanol (125 µL) and maleimide probe (40 µmol) were added. A solution of cell extract dissolved in methanol to achieve 2× concentration was added (125 µL). 130 µL of RO water was added followed by 5wt % NaOH (21.6 µL of 5 wt % NaOH). The reaction was heated to 50 °C and left to stir overnight (18 hours). The reaction mixture was brought to room temperature and quenched with 1M HCl. It was then

extracted with ethyl acetate (3 × 2mL) and concentrated. The crude mixture was taken up in 5.4 mL of methanol to be analyzed on the UPLC. Control reactions were run following this protocol in absence of the probe. These reactions were run in triplicate.

UPLC-MS was utilized to determine the amount of conversion based on leftover starting material by monitoring the absorbance at 254 nm. The UPLC analysis was carried out on a ACQUITY UPLC BEH C18 Column, 130Å, 1.7 µm, 2.1 mm × 50 mm (Waters) column. The trace was obtained by using mobile phases of H_2O + 0.1% formic acid (A) and acetonitrile + 0.1% formic acid (B). Samples were eluted using a gradient with mobile phase B ranging from 40% to 60% over 8.5 minutes at a flow rate of 0.5 mL/min. The column was equilibrated with 0% mobile phase B for 1 minute before and after the gradient. The samples were analyzed in increasing concentration of 3-furoic acid.

Maleimide probe Diels–Alder reaction in cell extract

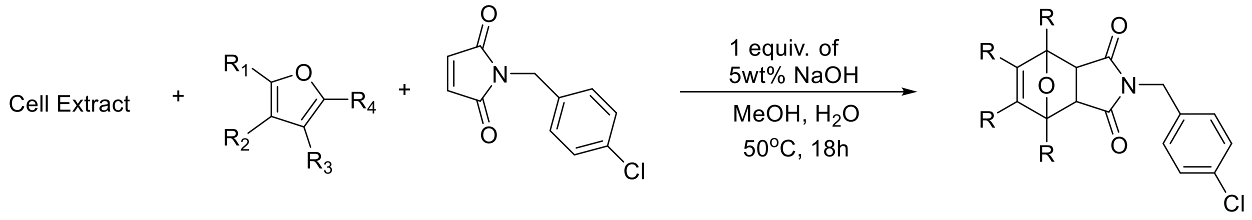

The initial volume of the *Streptomyces* growth prior to extraction was the volume for a 1× concentration. Volumes of solvent added were then reduced appropriately to obtain other concentrations (*e.g.* 2×, 5×, or 25×). In a 4 mL vial equipped with a stir bar, the maleimide probe (40 µmol) was added followed by the addition a solution of cell extract dissolved in methanol (250 µL). 130µL of RO water was added followed by 5 wt % NaOH (21.6 µL of 5 wt % NaOH). The reaction was heated to 50 °C and left to stir overnight (18 h). The reaction mixture was brought to room temperature and quenched with 1 M HCl. It was then extracted with ethyl acetate (3×) and concentrated. The crude mixture was taken up in 5.4 mL of methanol to be analyzed on the UPLC. Control reactions were run following this protocol but in absence of the probe. M145 and W75 were run in one replicate with 5× concentration, with a control, and with 25×. After seeing MMF cycloadduct via UPLC analysis with 25× of the W75 cell extracts, two more replicates were run with 25× concentrations of the M145 and W75 with controls at 25× as well.

Supporting figures

Furan S1

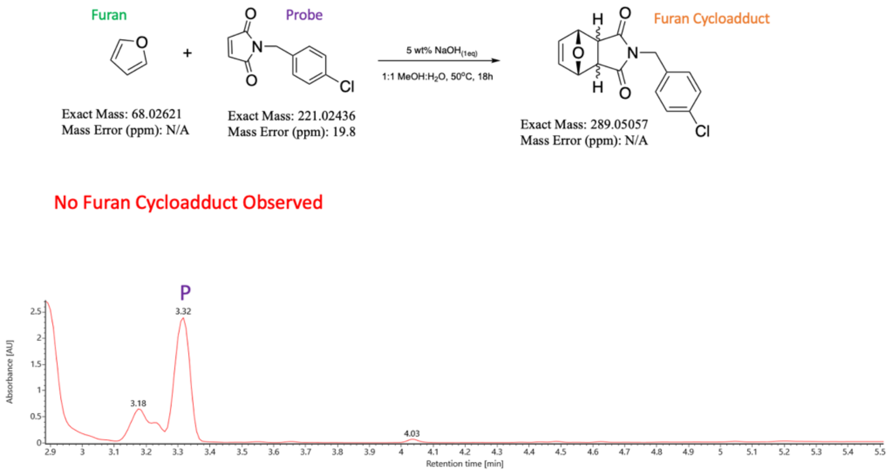

Figure S1. Furan. Representative mass spectra and UV traces for furan reacted with the maleimide probe (P). This may be because of the low boiling point of furan.

2-Furoic Acid S2

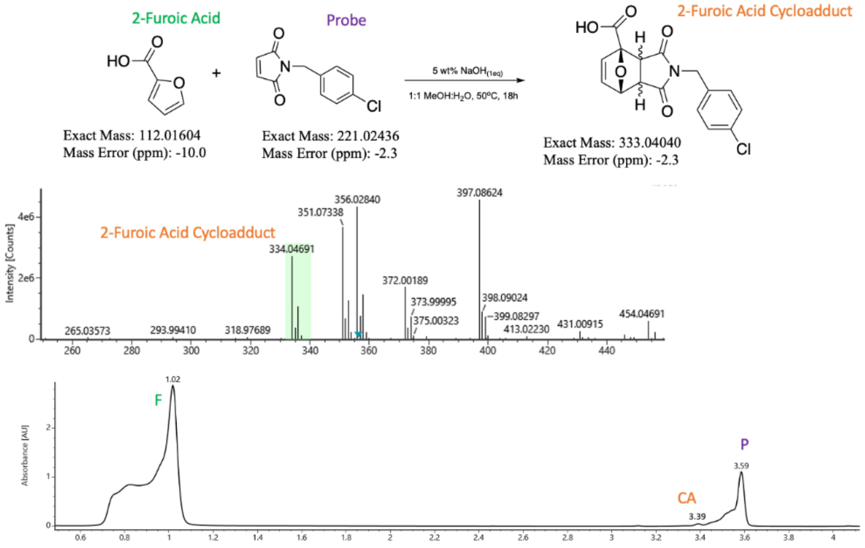

Figure S2. 2-Furoic acid. Representative mass spectra and UV traces for 2-furoic acid (F) reacted with the maleimide probe (P) to produce 2-furoic acid cycloadduct(CA).

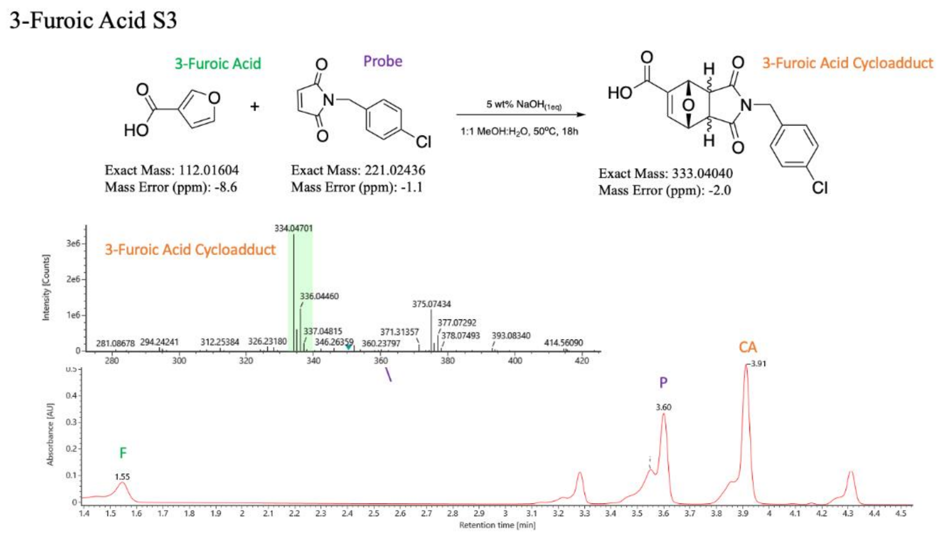

Figure S3. 3-Furoic Acid. Representative mass spectra and UV traces for 3-furoic acid (F) reacted with the maleimide probe (P) to produce 3-furoic acid cycloadduct (CA).

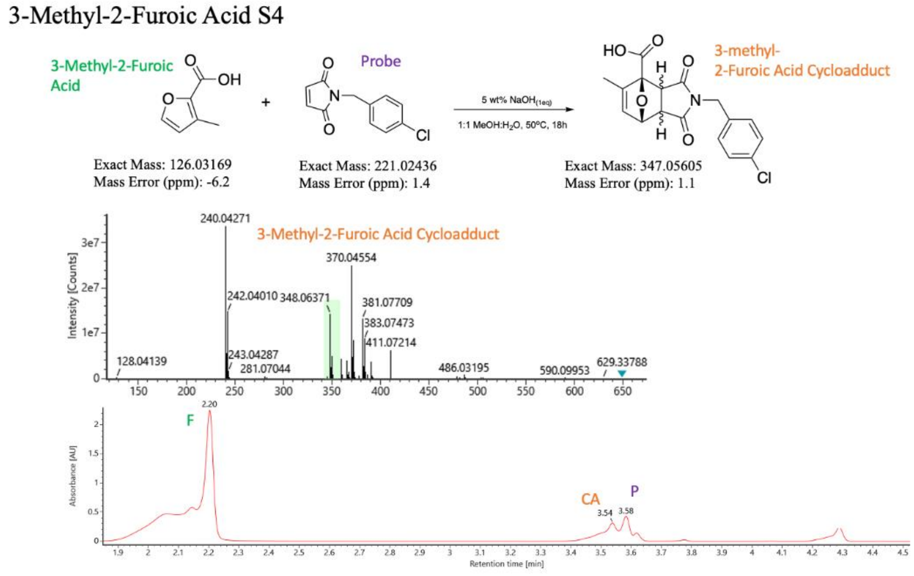

Figure S4. 3-Methyl-2-Furoic Acid. Representative mass spectra and UV traces for 3-methyl-2-furoic acid (F) reacted with the maleimide probe (P) to produce 3-methyl-2-furoic acid cycloadduct (CA).

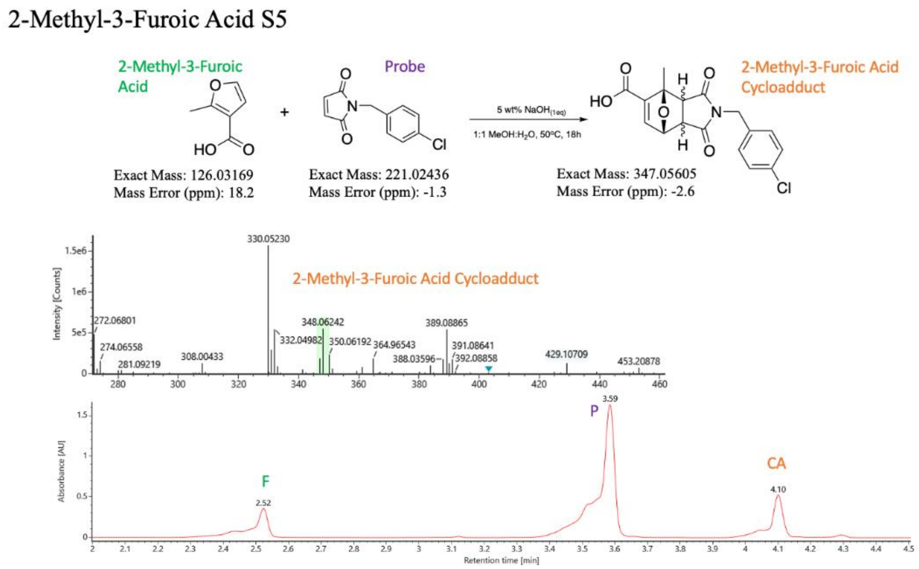

Figure S5. 2-Methyl-3-Furoic Acid. Representative mass spectra and UV traces for 2-methyl-3-furoic acid (F) reacted with the maleimide probe (P) to produce 2-methyl-3-furoic acid cycloadduct (CA).

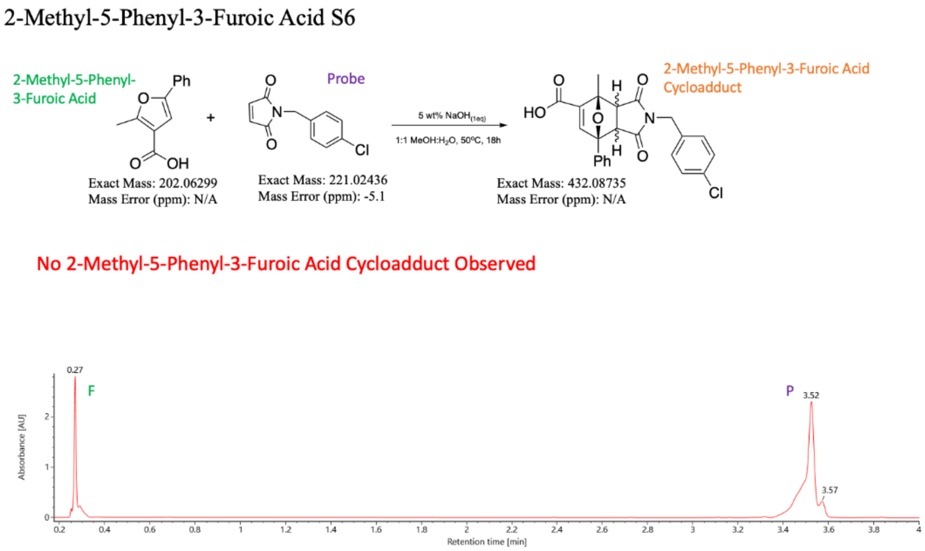

Figure S6. 2-Methyl-5-Phenyl-3-Furoic Acid. Representative mass spectra and UV traces for 2-methyl-5-phenyl-3-furoic acid (F) reacted with the maleimide probe (P).

Furfural S7

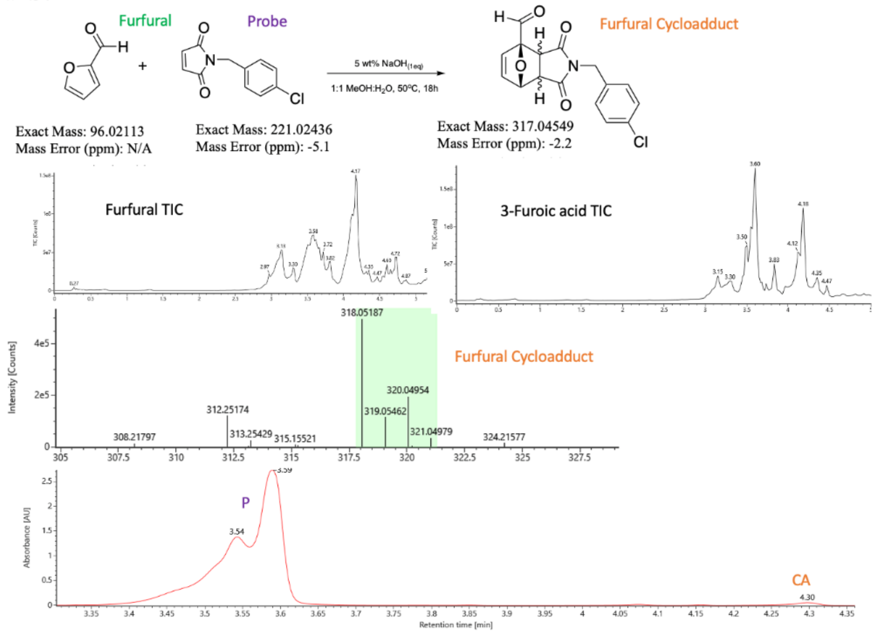

Figure S7. Furfural. Representative mass spectra compared to mass spectra of 3-furoic acid and UV traces furfural reacted with the maleimide probe (P) to produce furfural cycloadduct (CA).

(4-methylfuran-3-yl) Methanol S8

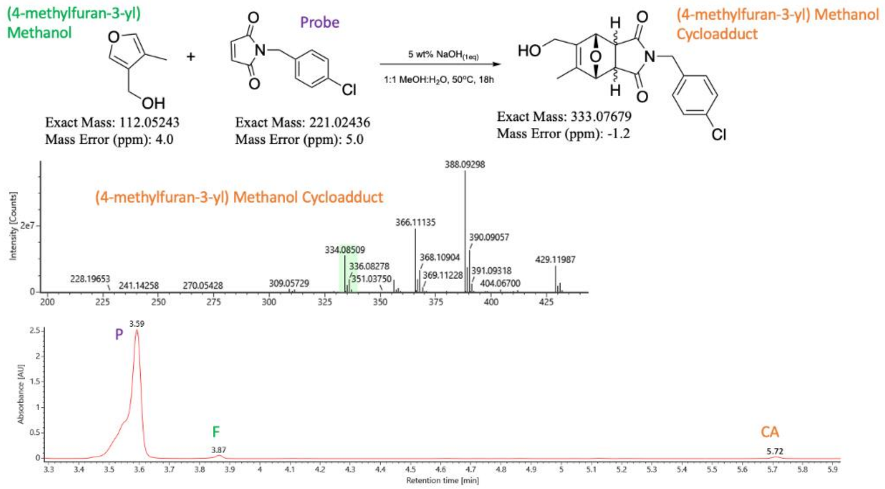

Figure S8. (4-Methylfuran-3-yl) Methanol. Representative mass spectra and UV traces (4-methylfuran-3-yl) methanol (F) reacted with the maleimide probe (P) to produce (4-methylfuran-3-yl) methanol cycloadduct (CA).

Methyl-3-Furan Carboxylate S9

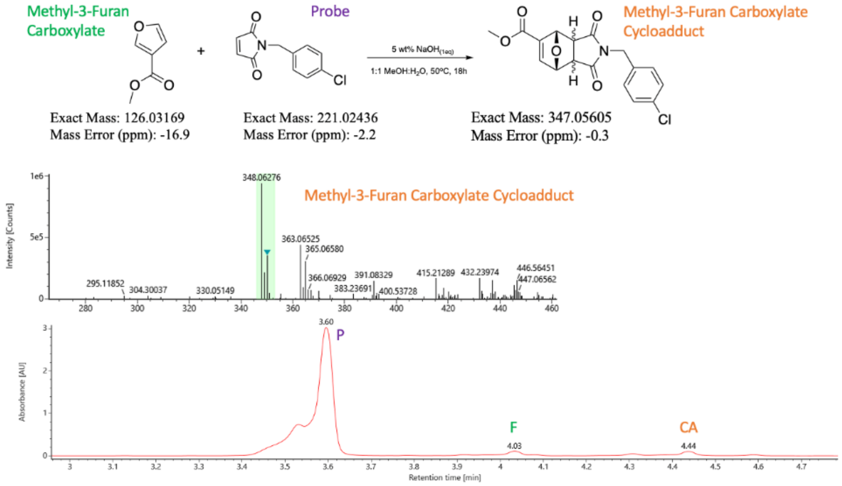

Figure S9. Methyl-3-Furan Carboxylate. Representative mass spectra and UV traces for methyl-3-furan carboxylate (F) reacted with the maleimide probe (P) to produce methyl-3-furan carboxylate cycloadduct (CA).

1H-Pyrrole S10

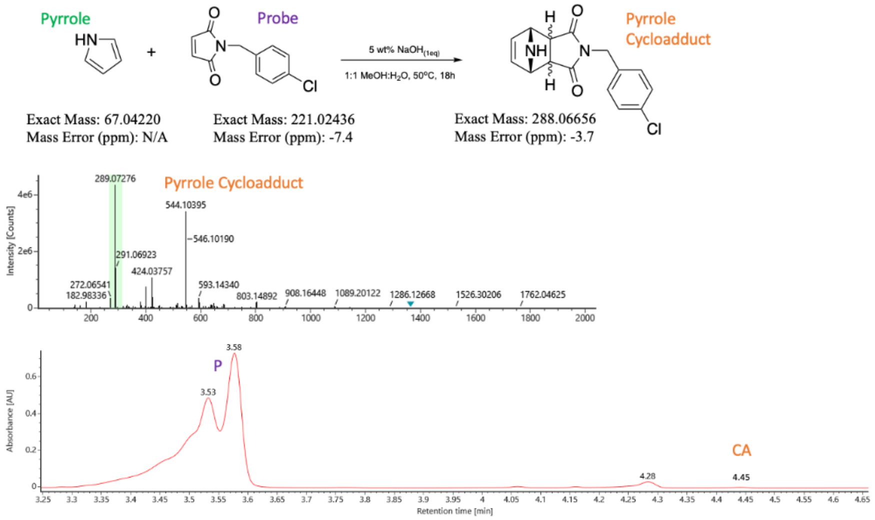

Figure S10. 1*H*-Pyrrole. Representative mass spectra and UV traces for 1H-pyrrole reacted with the maleimide probe (P) to produce 1H-pyrrole cycloadduct (CA).

Thiophene S11

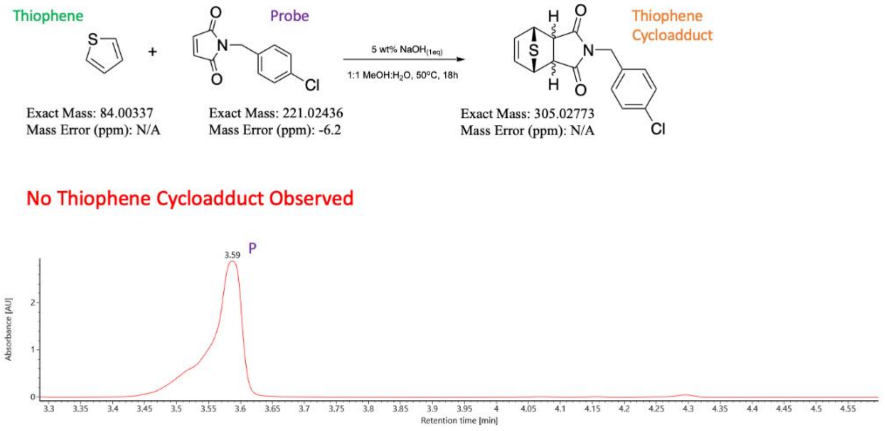

Figure S11. Thiophene. Representative mass spectra and UV traces for thiophene reacted with the maleimide probe (P).

MMF 1 S12

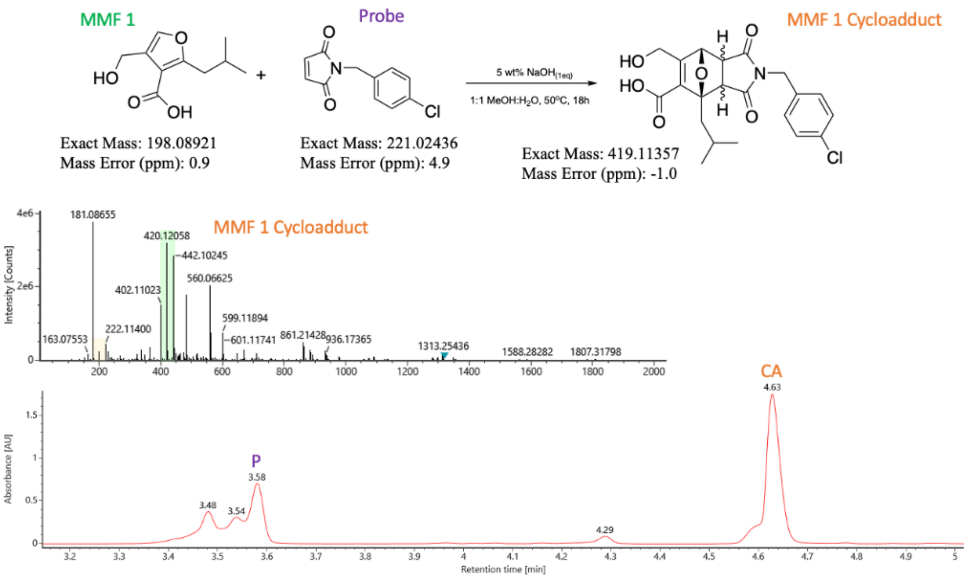

Figure S12. MMF1. Representative mass spectra and UV traces for MMF 1 reacted with the maleimide probe (P) to produce MMF 1 cycloadduct (CA).

MMF 2 S13

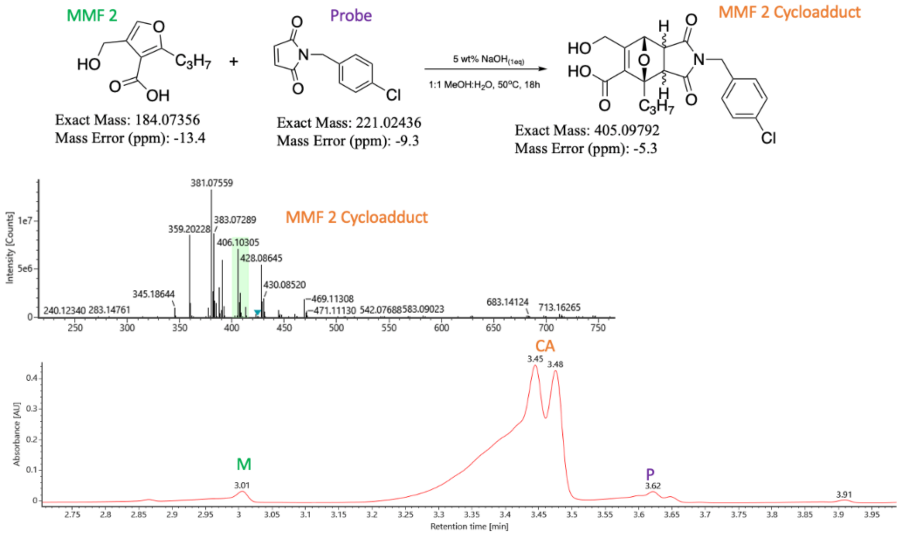

Figure S13. MMF2. Representative mass spectra and UV traces for MMF 2 (M) reacted with the maleimide probe (P) to produce MMF 2 cycloadduct (CA).

MMF 3 S14

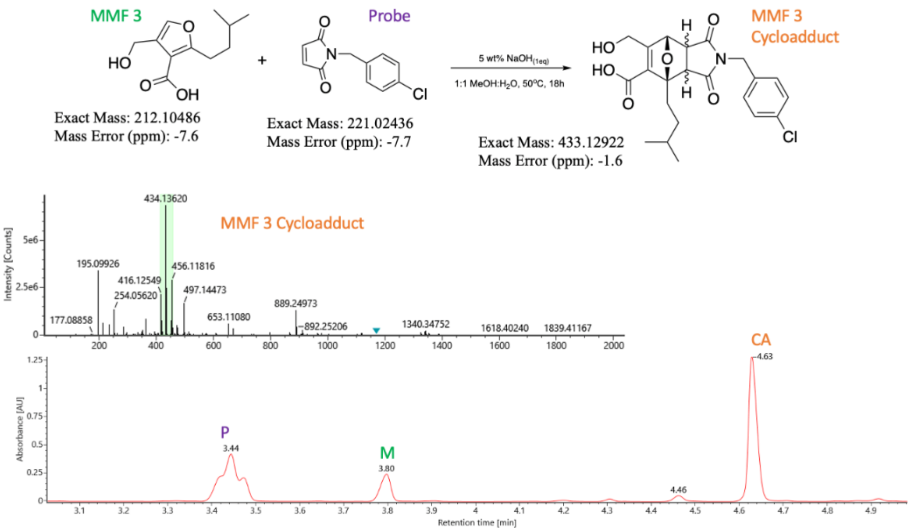

Figure S14. MMF3. Representative mass spectra and UV traces for MMF 3 (M) reacted with the maleimide probe (P) to produce MMF 3 cycloadduct (CA).

MMF 4 S15

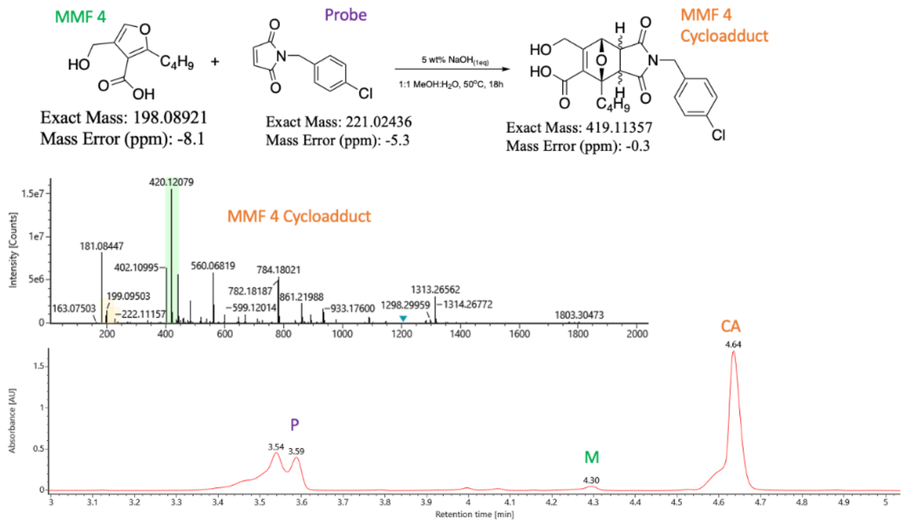

Figure S15. MMF4. Representative mass spectra and UV traces for MMF 4 (M) reacted with the maleimide probe (P) to produce MMF 4 cycloadduct (CA).

MMF 5 S16

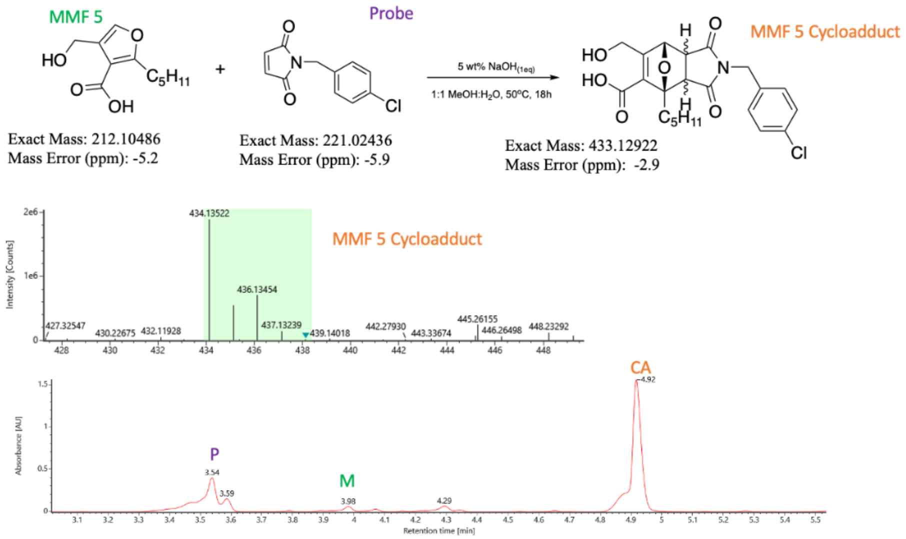

Figure S16. MMF5. Representative mass spectra and UV traces for MMF 5 (M) reacted with the maleimide probe (P) to produce MMF 5 cycloadduct (CA).

Der 1 S17

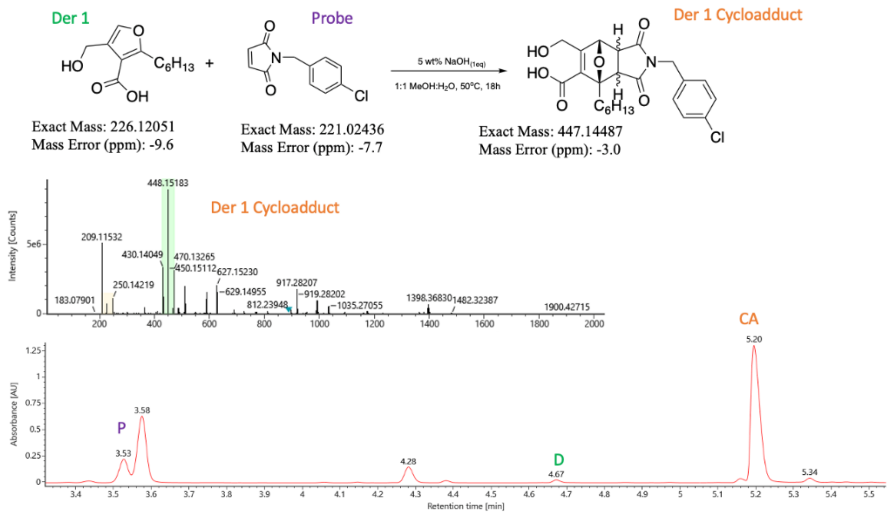

Figure S17. MMF Derivative 1. Representative mass spectra and UV traces for MMF derivative 1 (D) reacted with the maleimide probe (P) to produce Der 1 cycloadduct (CA).

Der 2 S18

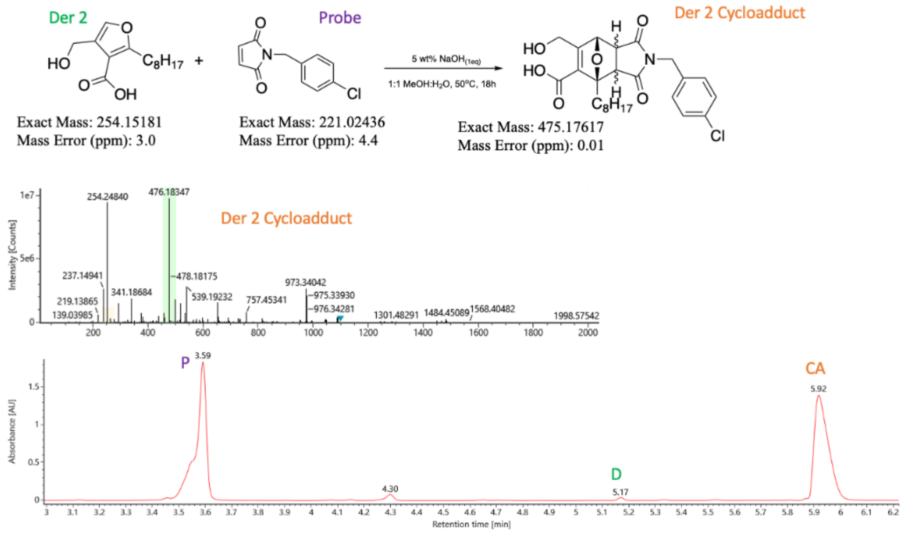

Figure S18. MMF Derivative 2. Representative mass spectra and UV traces for MMF derivative 2 (D) reacted with the maleimide probe (P) to produce Der 2 cycloadduct (CA).

Der 3 S19

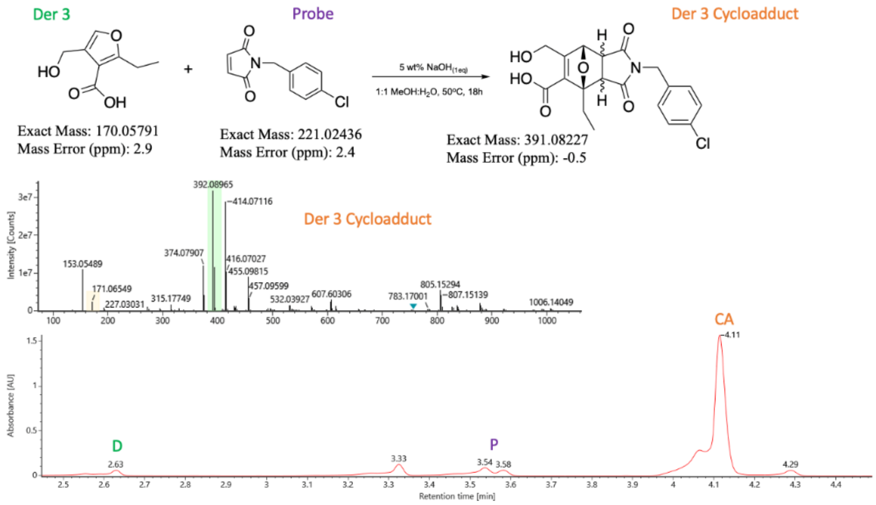

Figure S19. MMF Derivative 3. Representative mass spectra and UV traces for MMF derivative 3 (D) reacted with the maleimide probe (P) to produce Der 3 cycloadduct (CA).

Der 4 S20

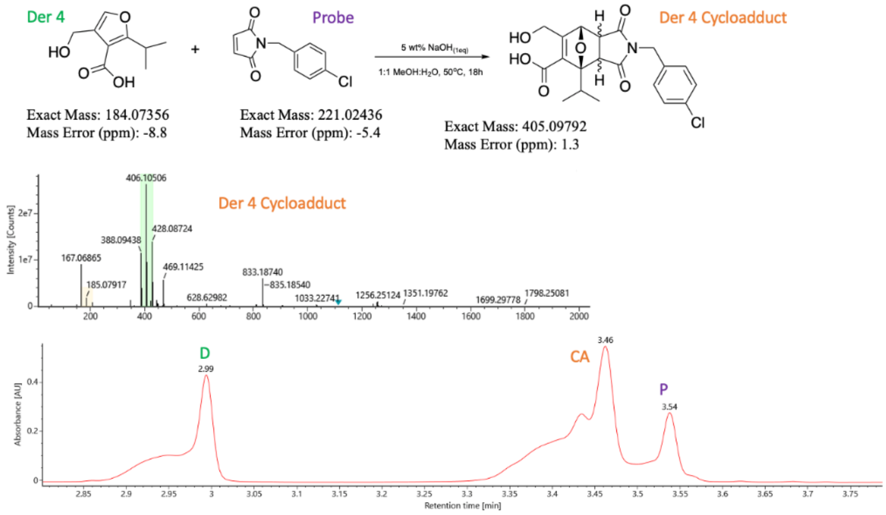

Figure S20. MMF Derivative 4. Representative mass spectra and UV traces for MMF derivative 4 (D) reacted with the maleimide probe (P) to produce Der 4 cycloadduct (CA).

Der 5 S21

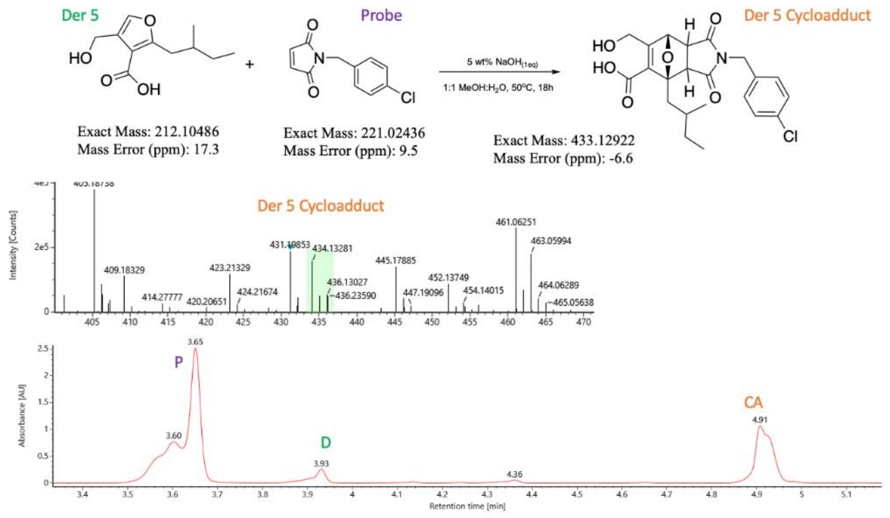

Figure S21. MMF Derivative 5. Representative mass spectra and UV traces for MMF derivative 5 (D) reacted with the maleimide probe (P) to produce Der 5 cycloadduct (CA).

Flufuran S22

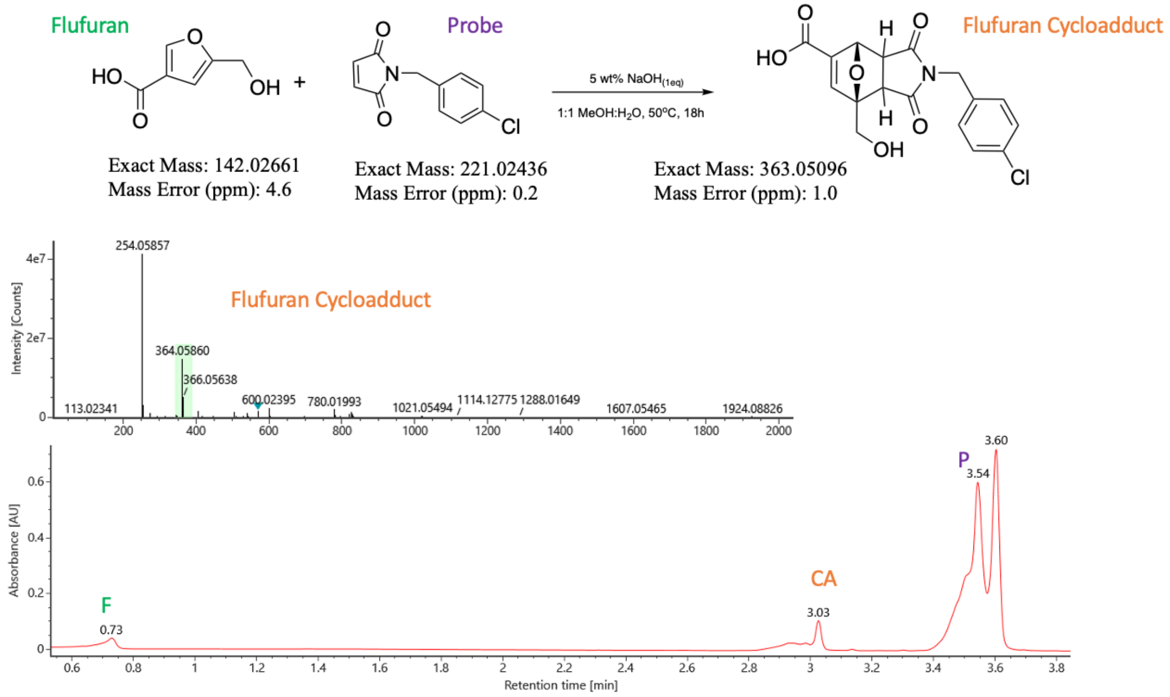

Figure S22. Flufuran. Representative mass spectra and UV traces for flufuran (F) reacted with the maleimide probe (P) to produce flufuran cycloadduct (CA).

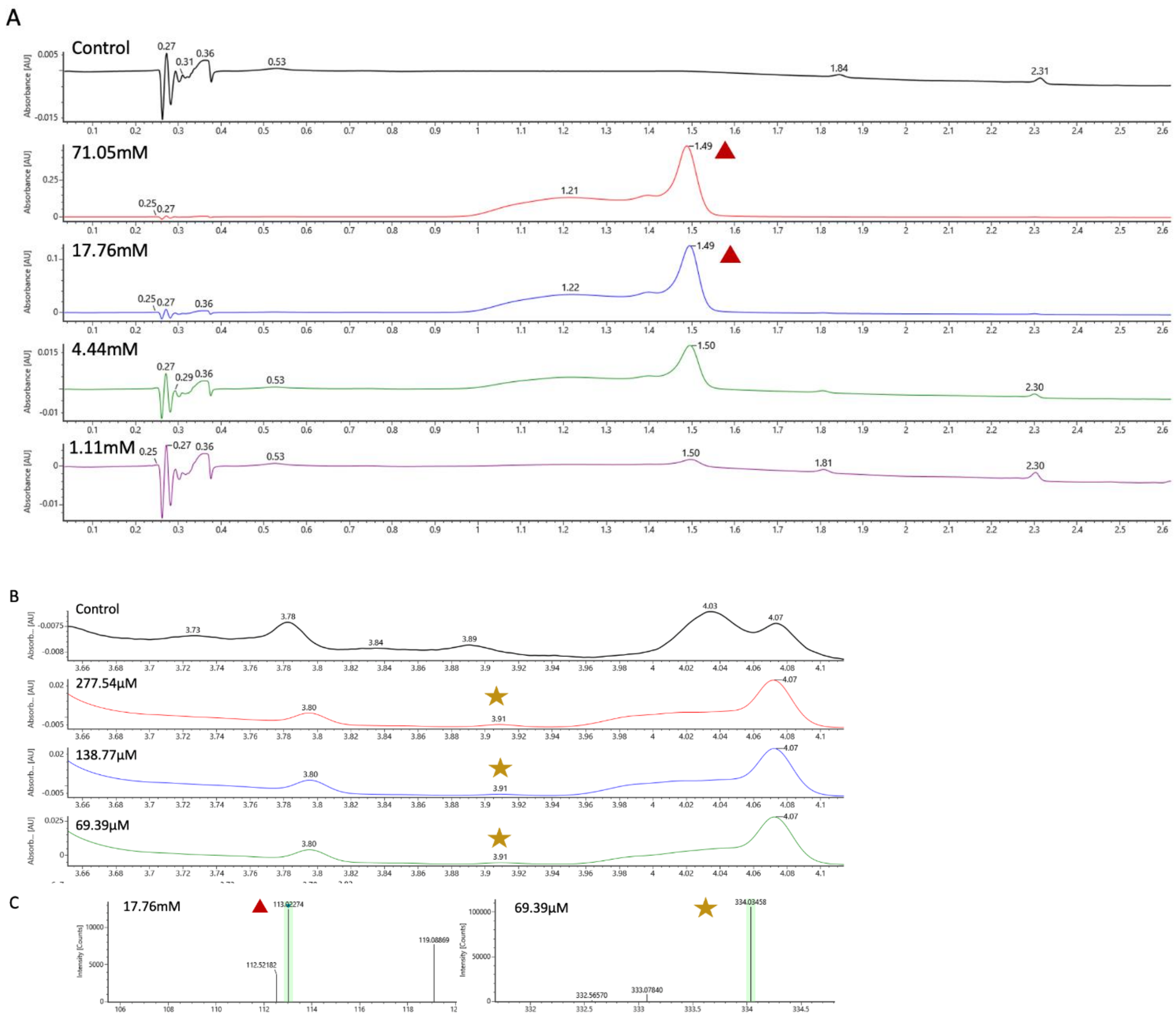

Figure S23. Limit of detection study.

A) UV traces at 254 nm of *Streptomyces coelicolor* M145 extract spiked with various concentrations of 3-Furoic Acid **(14)**, as well as a control that contained just the *Streptomyces coelicolor* extract. The lowest that the mass of **14** (red triangle) can be confidently observed is at 17.8 mM. B) UV traces at 254 nm of *Streptomyces coelicolor* M145 extract spiked with various concentrations of 14 and treated with the maleimide probe. The lowest that the mass of the cycloadduct (yellow star) can be confidently observed on the UPLC is at 69.4 µM. The control is only *Streptomyces coelicolor* extract. C.) Shows the mass of 3-furoic acid at 17.8 mM and the mass of 3-furoic acid cycloadduct at 69.4 µM.

References

1. Davis JB, Bailey JD, Sello JK (2009) Biomimetic Synthesis of a New Class of Bacterial Signaling Molecules. Org Lett 14:2984–2987

2. Corre C, Song L, O'Rourke S, et al (2008) 2-Alkyl-4-hydroxymethylfuran-3-carboxylic acids, antibiotic production inducers discovered by Streptomyces coelicolor genome mining. Proc Natl Acad Sci U S A 105:17510–17515. https://doi.org/https://doi.org/10.1073/pnas.080553010

3. Zhou S, Bhukya H, Malet N, et al (2021) Molecular basis for control of antibiotic production by a bacterial hormone. Nature 590:463–467. https://doi.org/10.1038/s41586-021-03195-x

4. Lin Z, Li J, Huang Q, et al (2015) Chiral Surfactant-Type Catalyst: Enantioselective Reduction of Long-Chain Aliphatic Ketoesters in Water. J Org Chem 80:4419–4429. https://doi.org/10.1021/acs.joc.5b00241

5. Mullah KB, Sutherland JK (1992) A simple synthesis of anthracyclinones from 2-acetylquinizarin. J Chem Soc Perkin 1 1237. https://doi.org/10.1039/p19920001237

6. Sagyam RR, Padi PR, Ghanta MR, Vurimidi H (2007) An efficient synthesis of highly substituted pyrrole and bis pyrrole derivatives. J Heterocycl Chem 44:923–926. https://doi.org/10.1002/jhet.5570440429

7. Schweizer E, Hoffmann-Röder A, Olsen JA, et al (2006) Multipolar interactions in the D pocket of thrombin: large differences between tricyclic imide and lactam inhibitors. Org Biomol Chem 4:2364–2375. https://doi.org/10.1039/B602585D

NMR Spectra

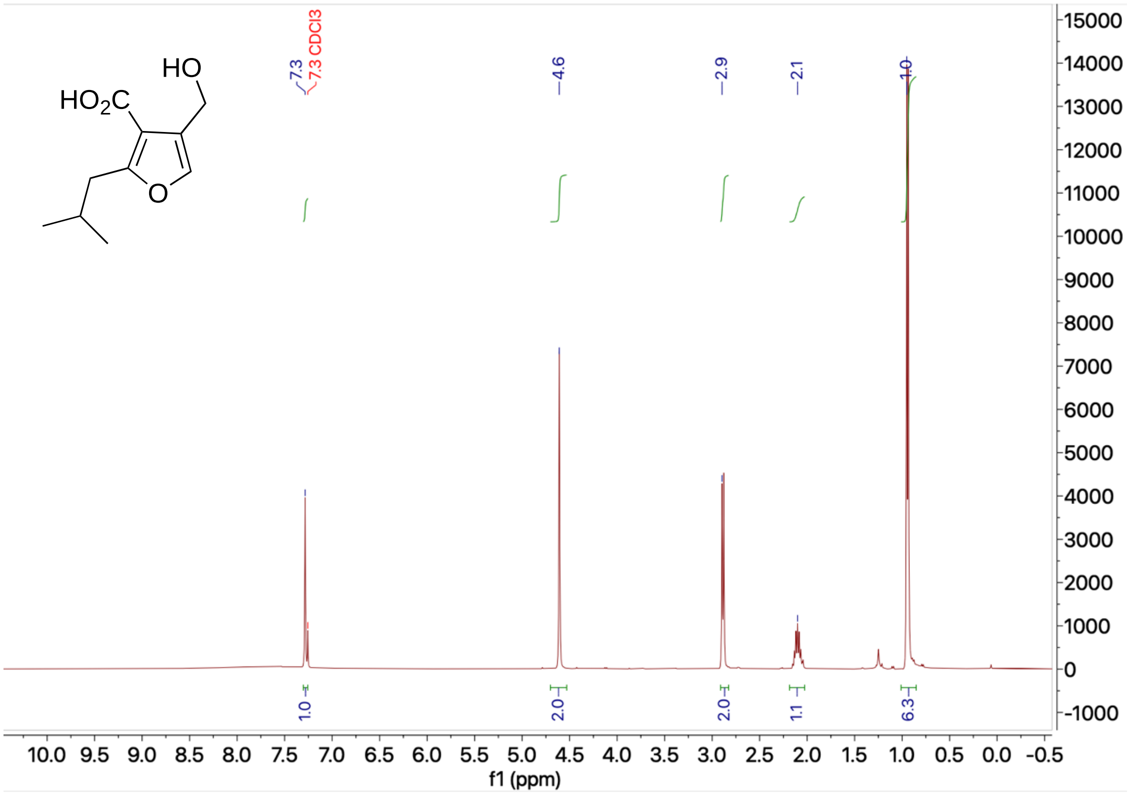

HO
HO_2C
O
7.3
7.3 CDCl3
4.6
2.9
2.1
1.0
1.0
2.0
2.0
1.1
6.3
15000
14000
13000
12000
11000
10000
9000
8000
7000
6000
5000
4000
3000
2000
1000
0
-1000
10.0 9.5 9.0 8.5 8.0 7.5 7.0 6.5 6.0 5.5 5.0 4.5 4.0 3.5 3.0 2.5 2.0 1.5 1.0 0.5 0.0 -0.5
f1 (ppm)

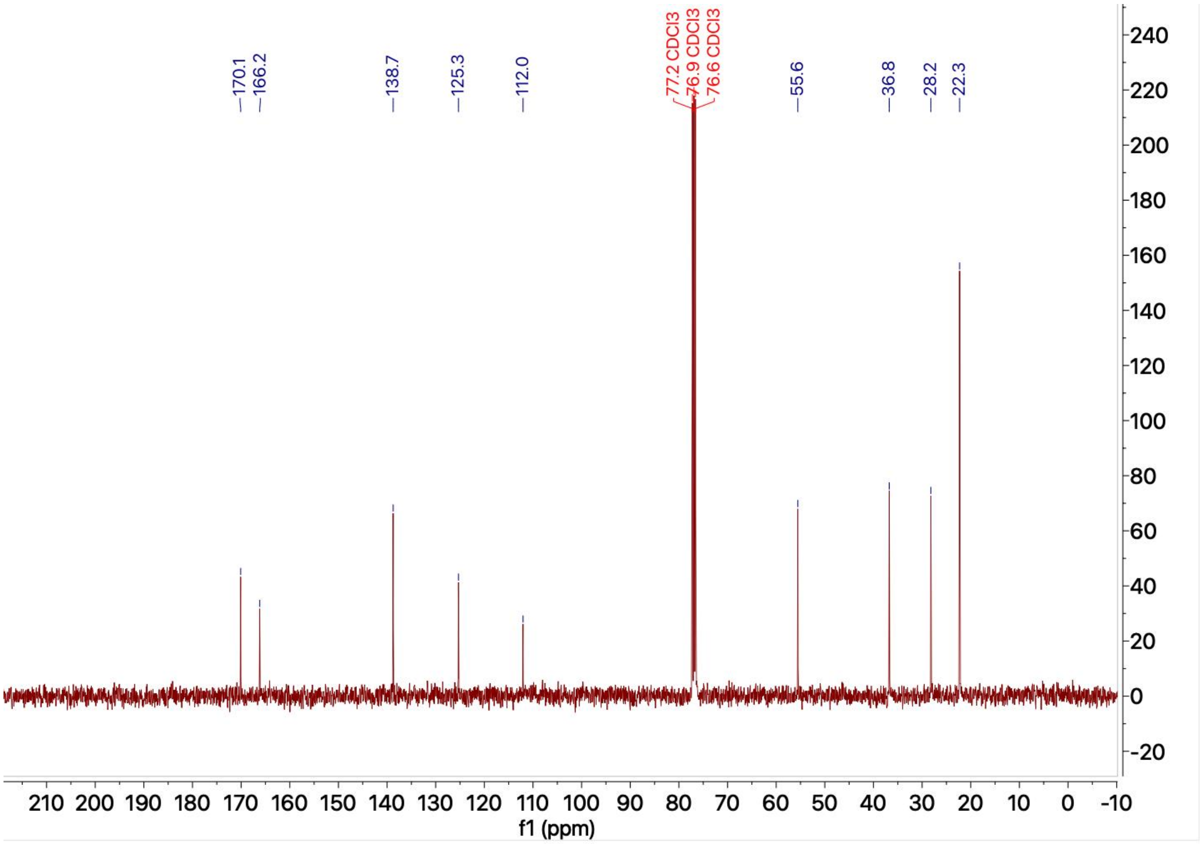

170.1
166.2
138.7
125.3
112.0
77.2 CDCl3
76.9 CDCl3
76.6 CDCl3
55.6
36.8
28.2
22.3
240
220
200
180
160
140
120
100
80
60
40
20
0
-20
210 200 190 180 170 160 150 140 130 120 110 100 90 80 70 60 50 40 30 20 10 0 -10
f1 (ppm)

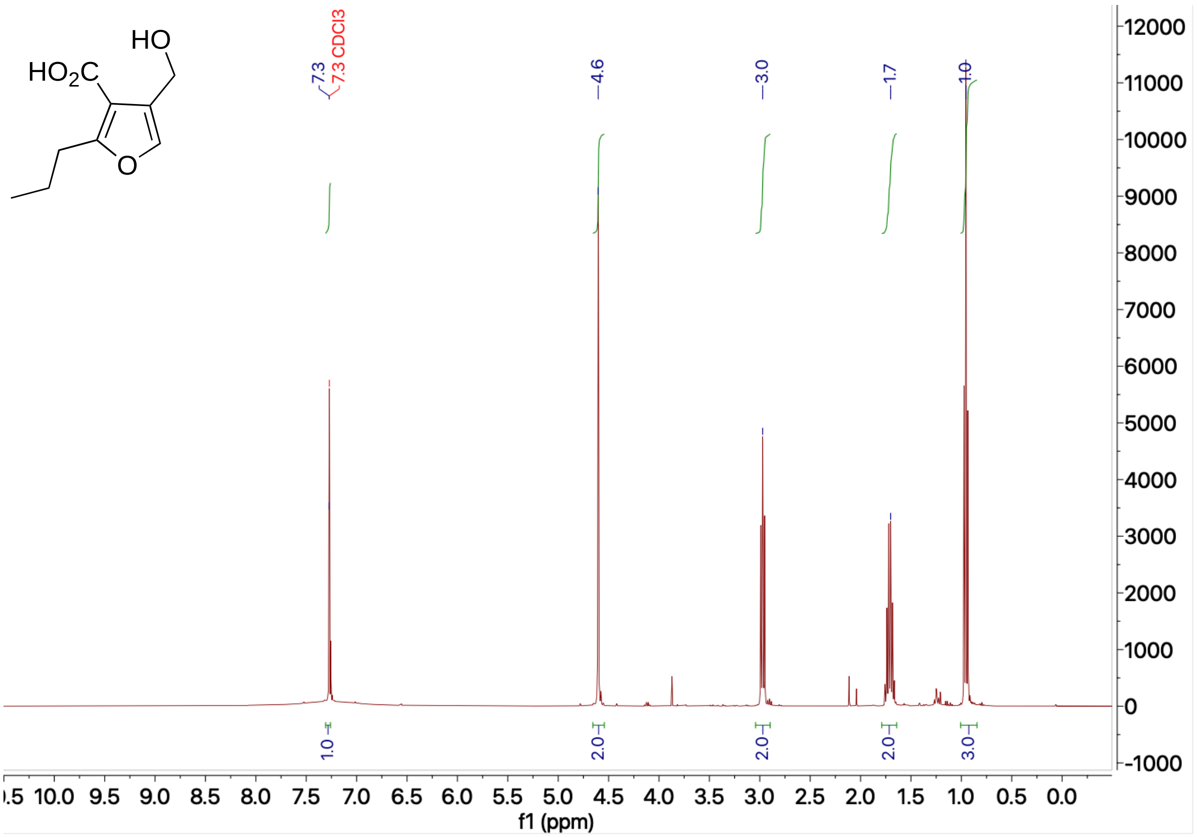
HO
HO2C
O
7.3
7.3 CDCl3
4.6
3.0
1.7
1.0
1.0
2.0
2.0
2.0
3.0
f1 (ppm)

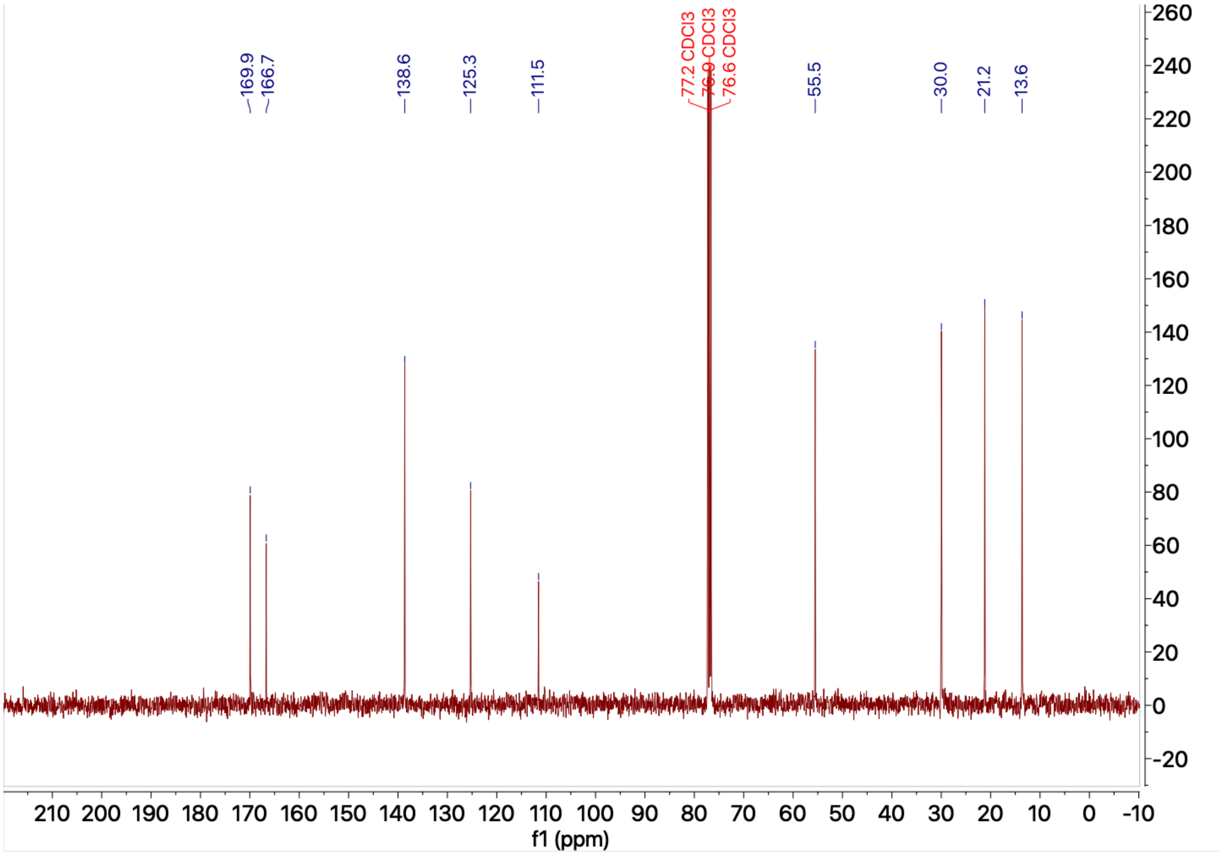
169.9
166.7
138.6
125.3
111.5
77.2 CDCl3
76.9 CDCl3
76.6 CDCl3
55.5
30.0
21.2
13.6
f1 (ppm)

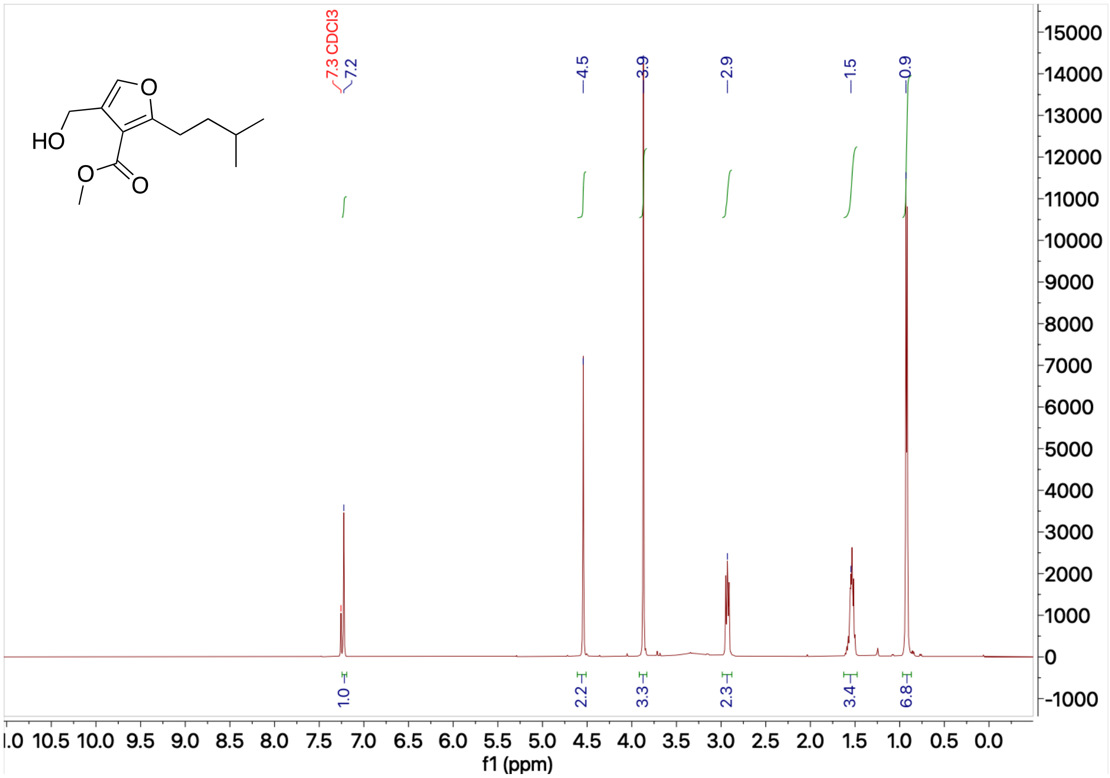
HO
O
O
O
7.3 CDCl3
7.2
4.5
3.9
2.9
1.5
0.9
1.0
2.2
3.3
2.3
3.4
6.8
f1 (ppm)

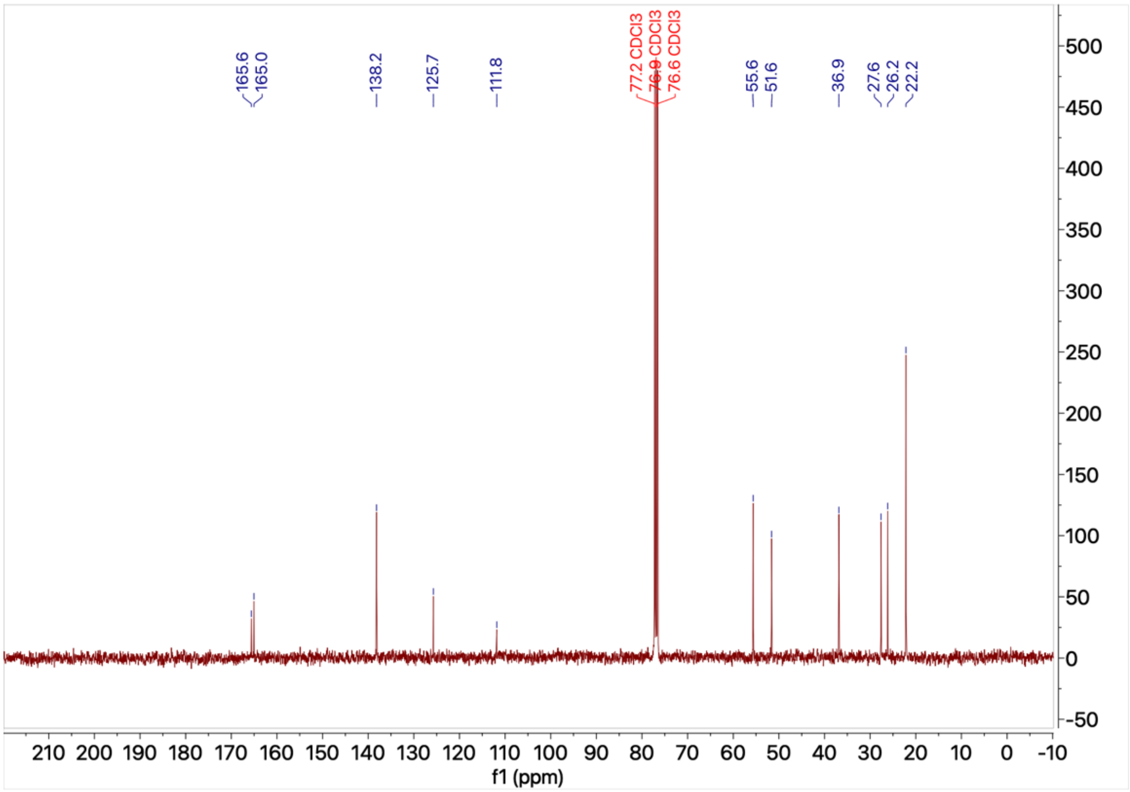
165.6
165.0
138.2
125.7
111.8
77.2 CDCl3
76.9 CDCl3
76.6 CDCl3
55.6
51.6
36.9
27.6
26.2
22.2
f1 (ppm)

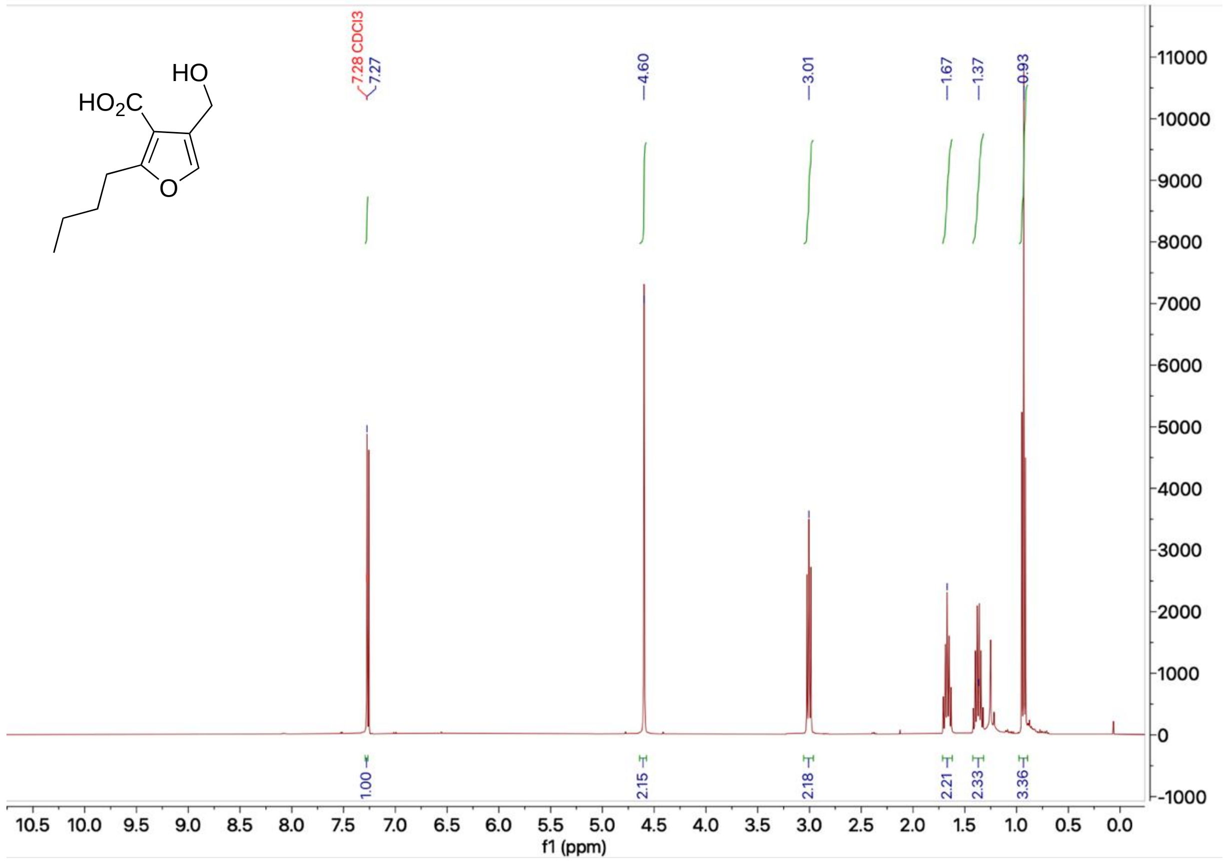
HO
HO_2C
O
7.28 CDCl3
7.27
4.60
3.01
1.67
1.37
0.93
1.00
2.15
2.18
2.21
2.33
3.36
f1 (ppm)

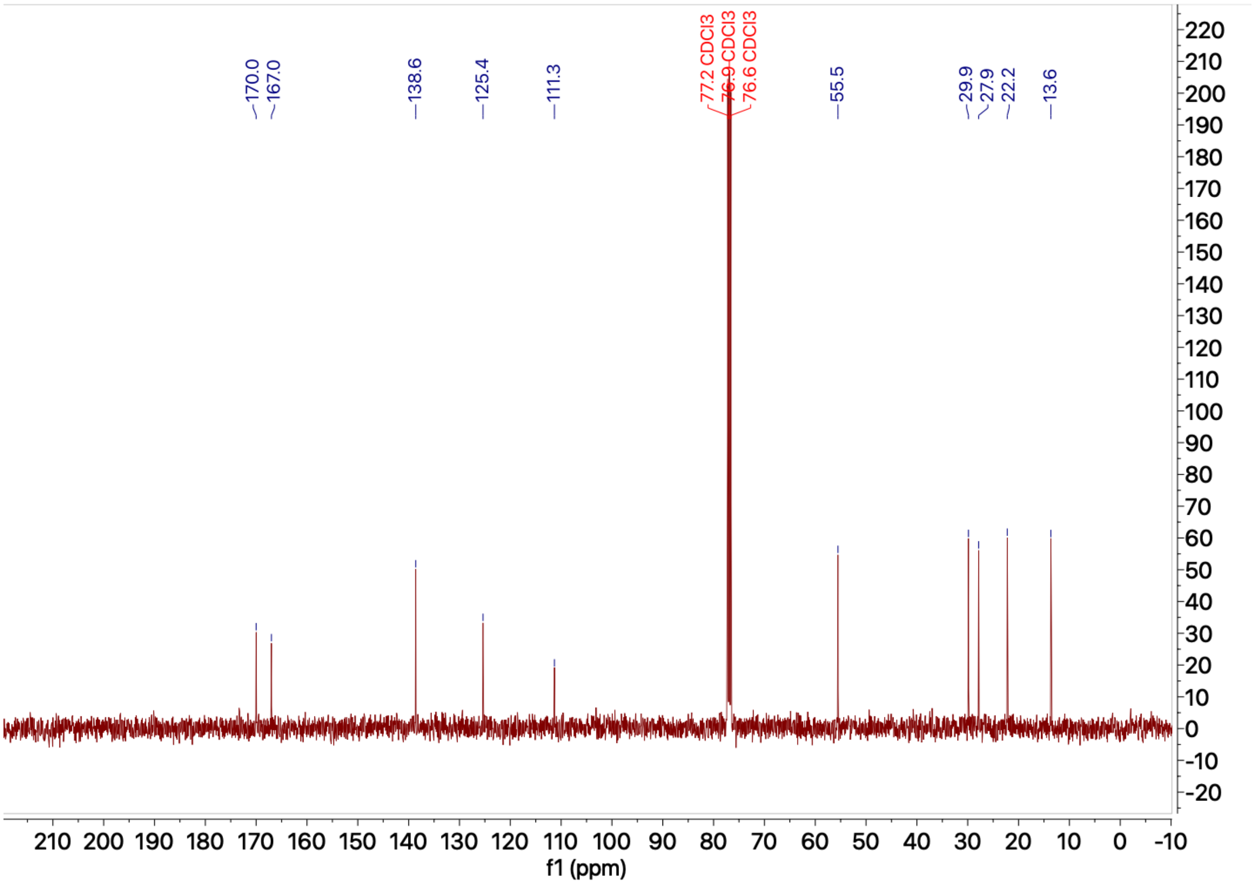
170.0
167.0
138.6
125.4
111.3
77.2 CDCl3
76.9 CDCl3
76.6 CDCl3
55.5
29.9
27.9
22.2
13.6
f1 (ppm)

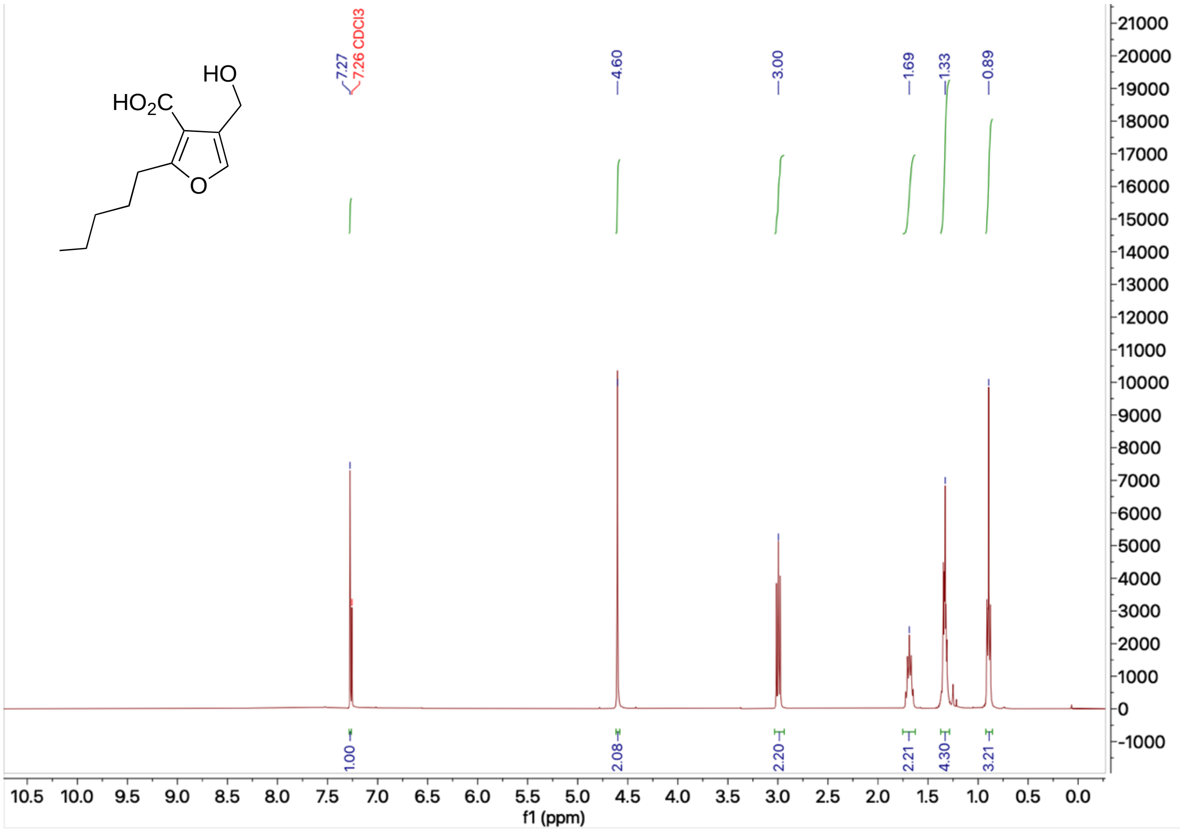
HO
HO_2C
O
7.27
7.26 CDCl3
4.60
3.00
1.69
1.33
0.89
1.00
2.08
2.20
2.21
4.30
3.21
f1 (ppm)

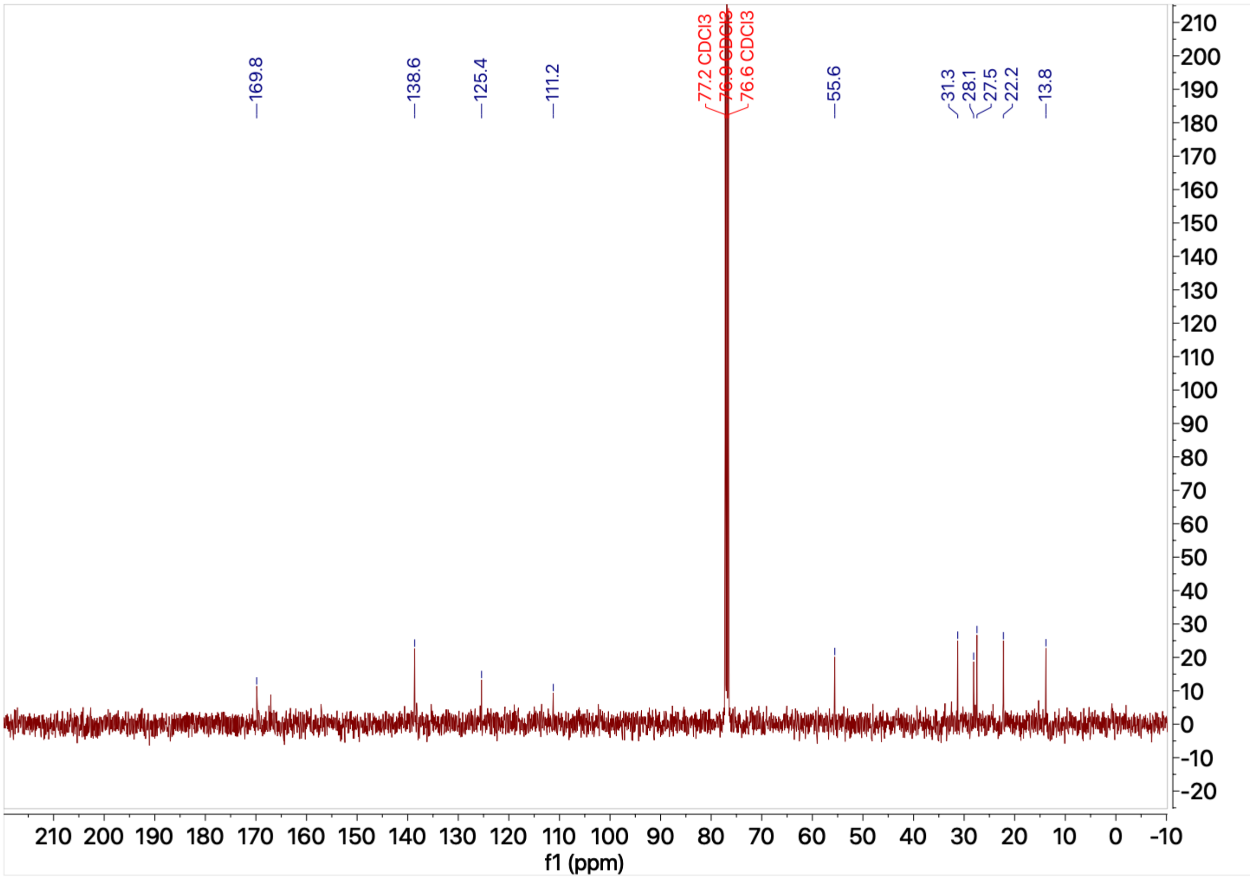
169.8
138.6
125.4
111.2
77.2 CDCl3
76.9 CDCl3
76.6 CDCl3
55.6
31.3
28.1
27.5
22.2
13.8
f1 (ppm)

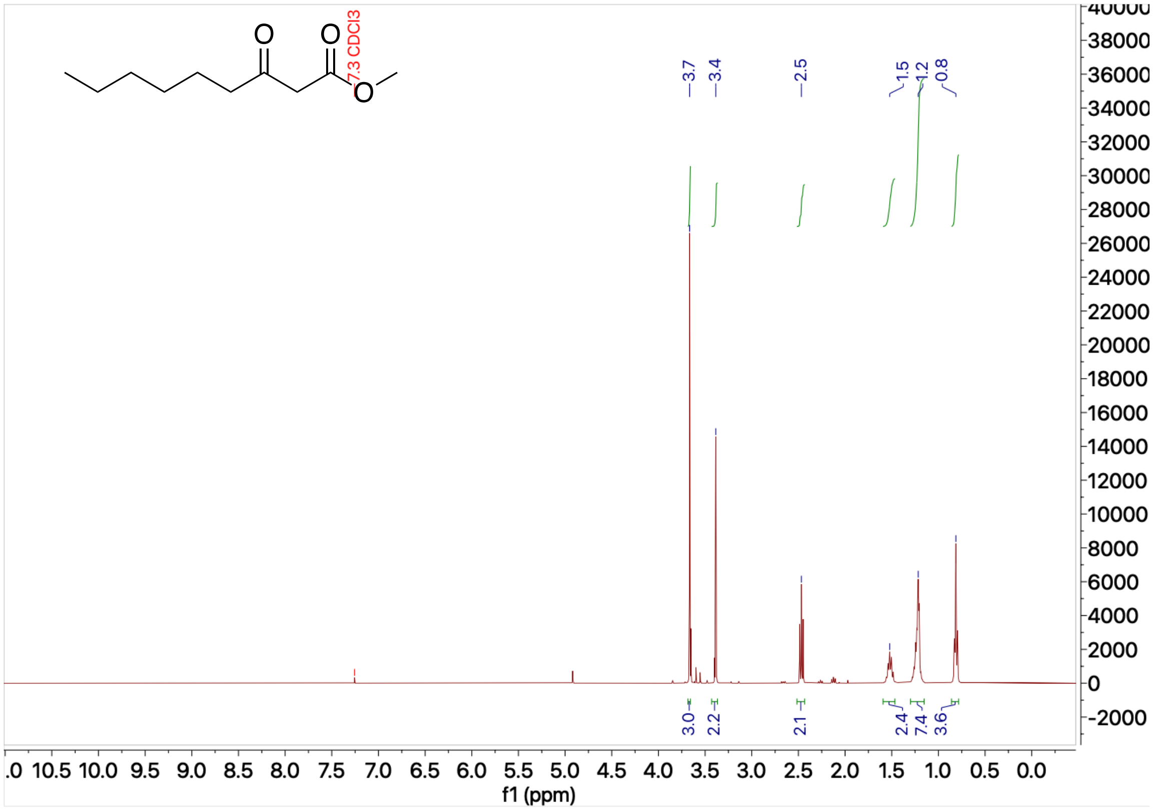

7.3 CDCl3
3.7
3.4
2.5
1.5
1.2
0.8
3.0
2.2
2.1
2.4
7.4
3.6
f1 (ppm)

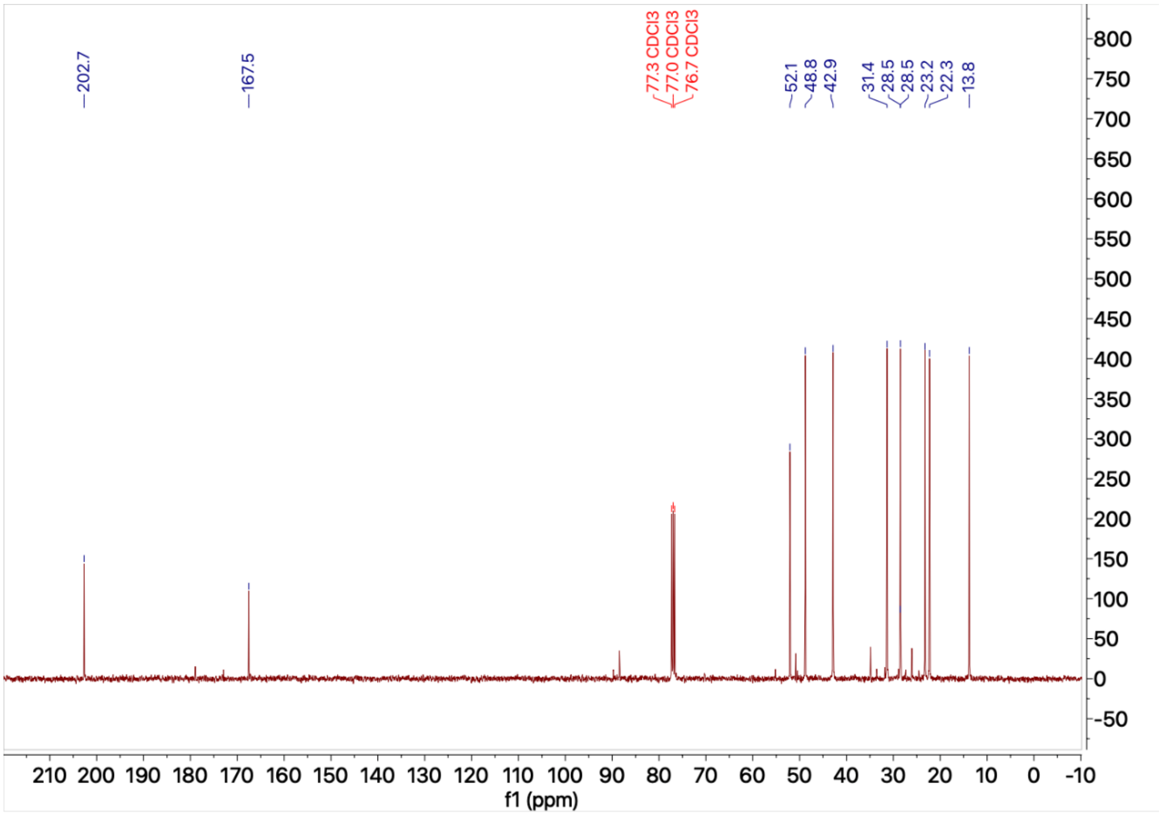

202.7
167.5
77.3 CDCl3
77.0 CDCl3
76.7 CDCl3
52.1
48.8
42.9
31.4
28.5
28.5
23.2
22.3
13.8
f1 (ppm)

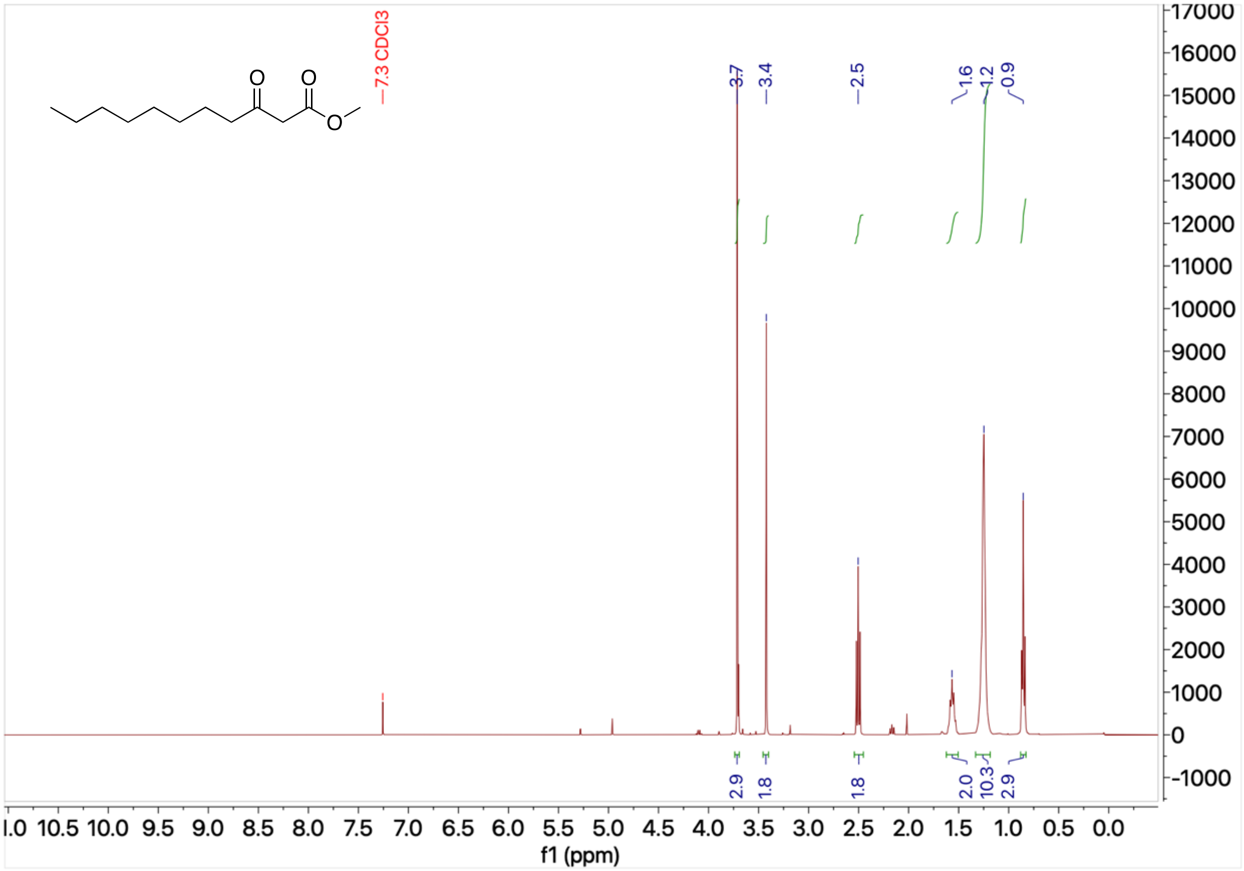

-7.3 CDCl3
3.7
3.4
2.5
1.6
1.2
0.9
2.9
1.8
1.8
2.0
10.3
2.9
f1 (ppm)

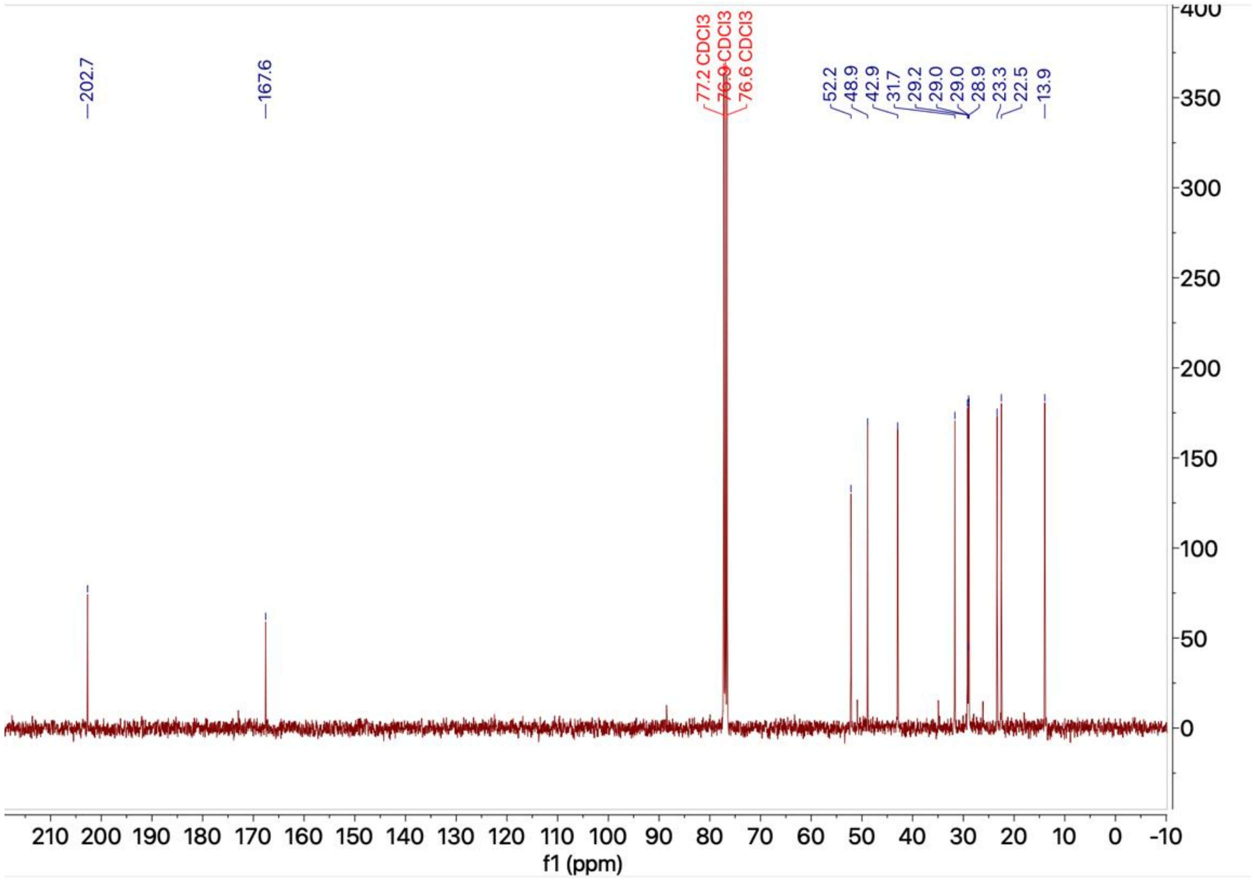

202.7
167.6
77.2 CDCl3
76.9 CDCl3
76.6 CDCl3
52.2
48.9
42.9
31.7
29.2
29.0
29.0
28.9
23.3
22.5
13.9
f1 (ppm)

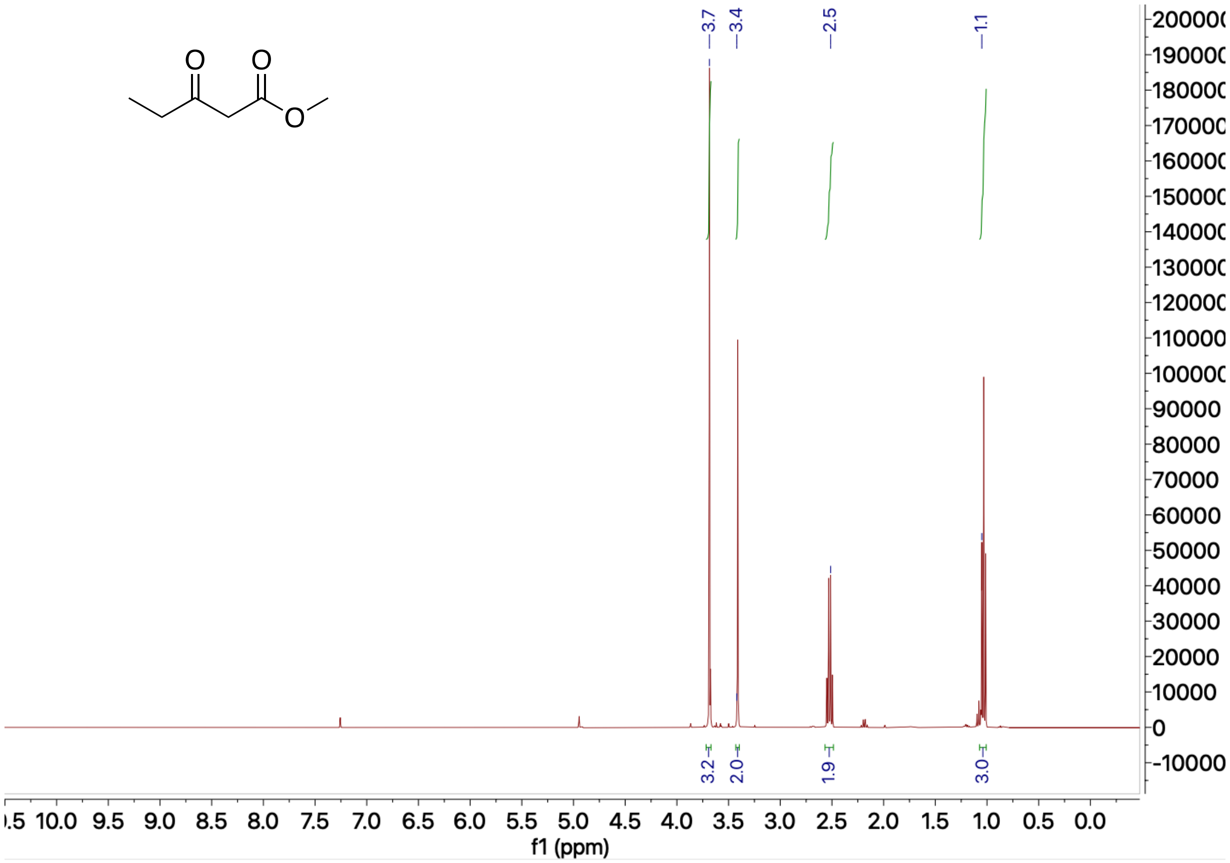
3.7
3.4
2.5
1.1
3.2
2.0
1.9
3.0
f1 (ppm)

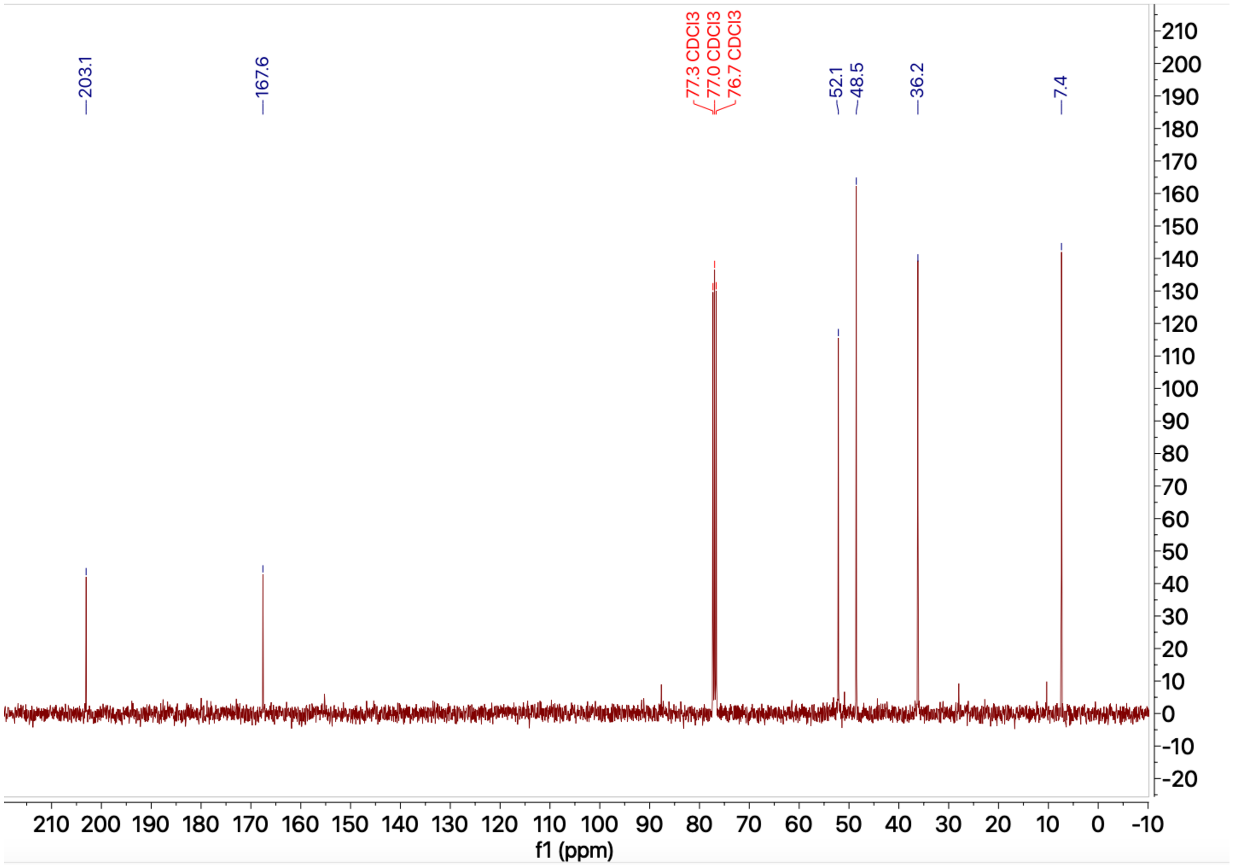
203.1
167.6
77.3 CDCl3
77.0 CDCl3
76.7 CDCl3
52.1
48.5
36.2
7.4
f1 (ppm)

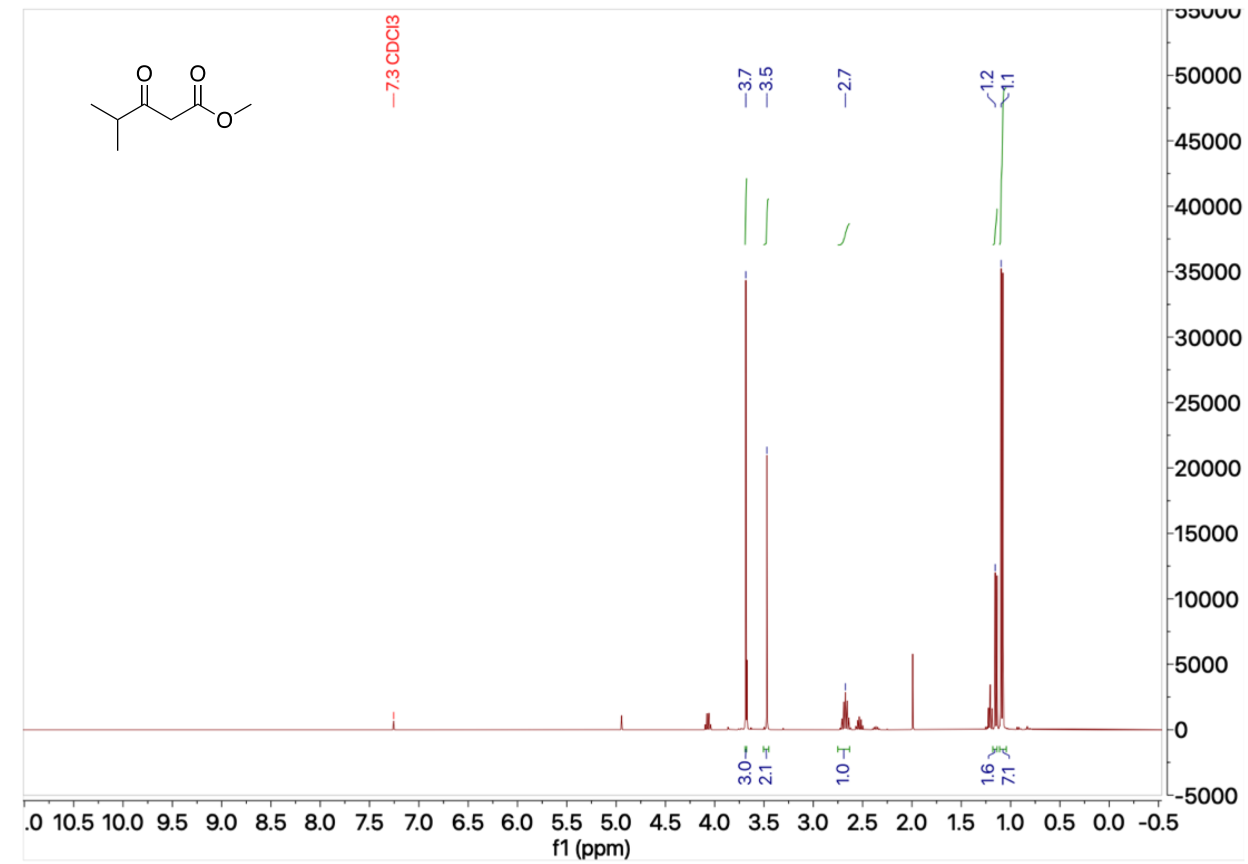

7.3 CDCl3
3.7
3.5
2.7
1.2
1.1
3.0
2.1
1.0
1.6
7.1
f1 (ppm)

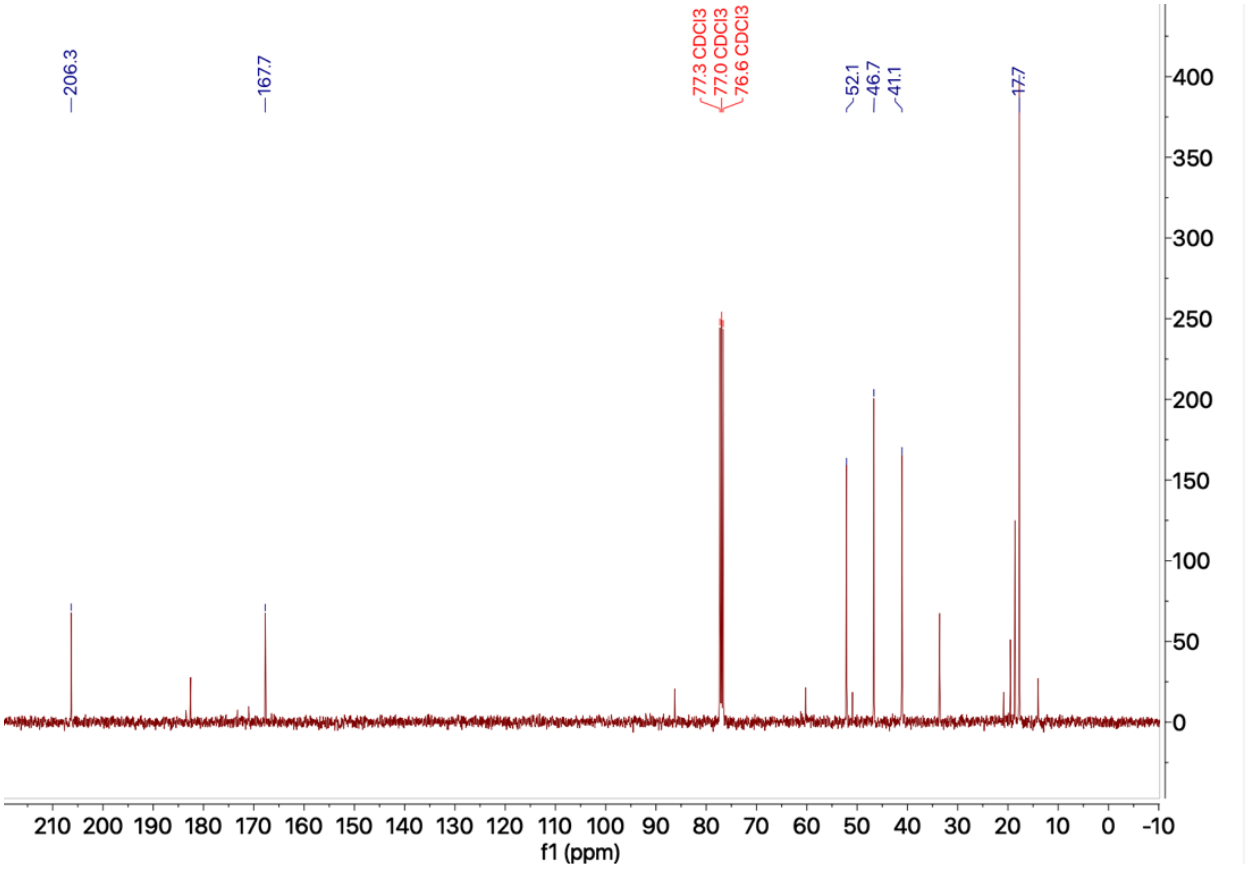

206.3
167.7
77.3 CDCl3
77.0 CDCl3
76.6 CDCl3
52.1
46.7
41.1
17.7
f1 (ppm)

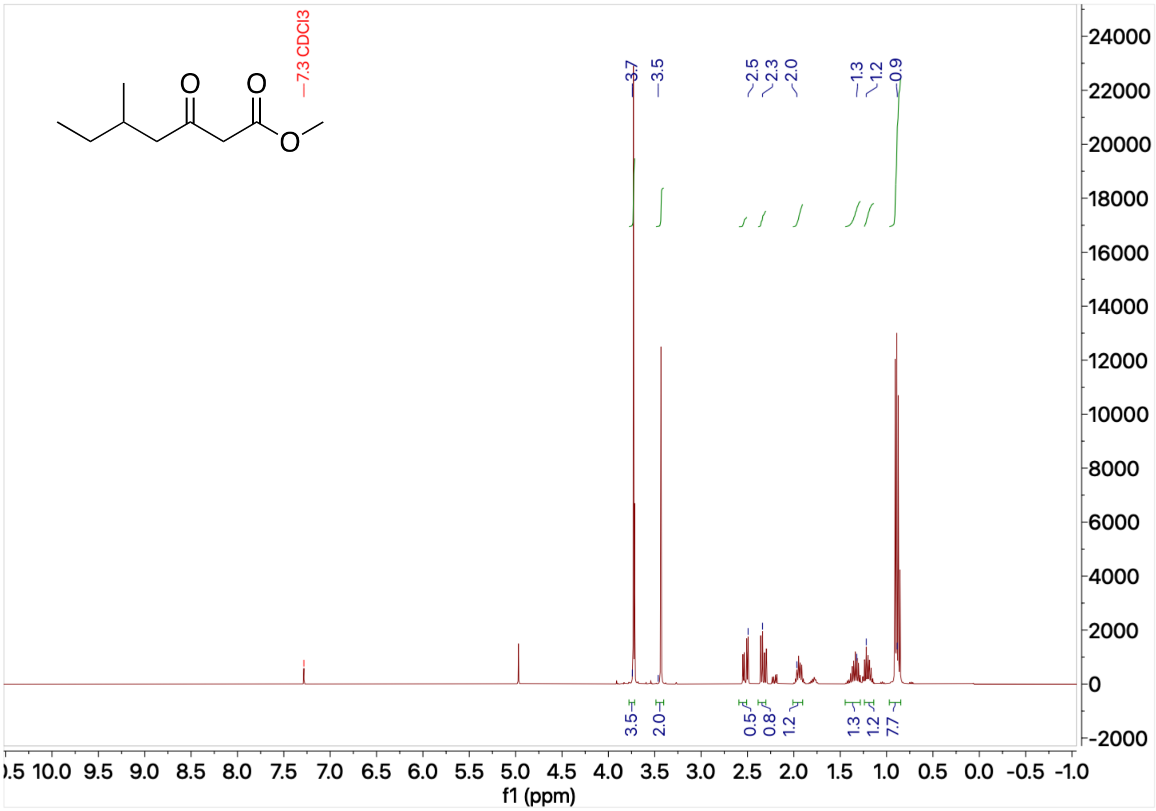
O
O
O
7.3 CDCl3
3.7
3.5
2.5
2.3
2.0
1.3
1.2
0.9
3.5
2.0
0.5
0.8
1.2
1.3
1.2
7.7
f1 (ppm)

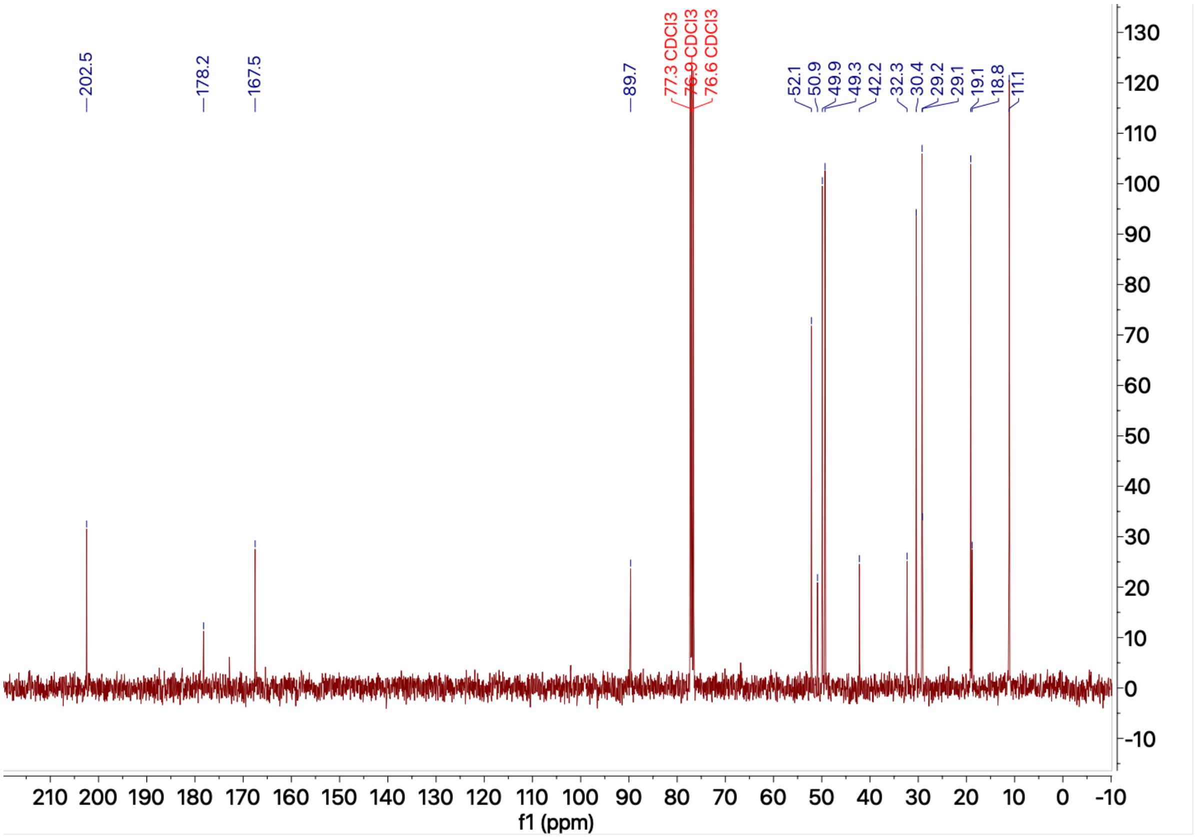
202.5
178.2
167.5
89.7
77.3 CDCl3
76.9 CDCl3
76.6 CDCl3
52.1
50.9
49.9
49.3
42.2
32.3
30.4
29.2
29.1
19.1
18.8
11.1
f1 (ppm)

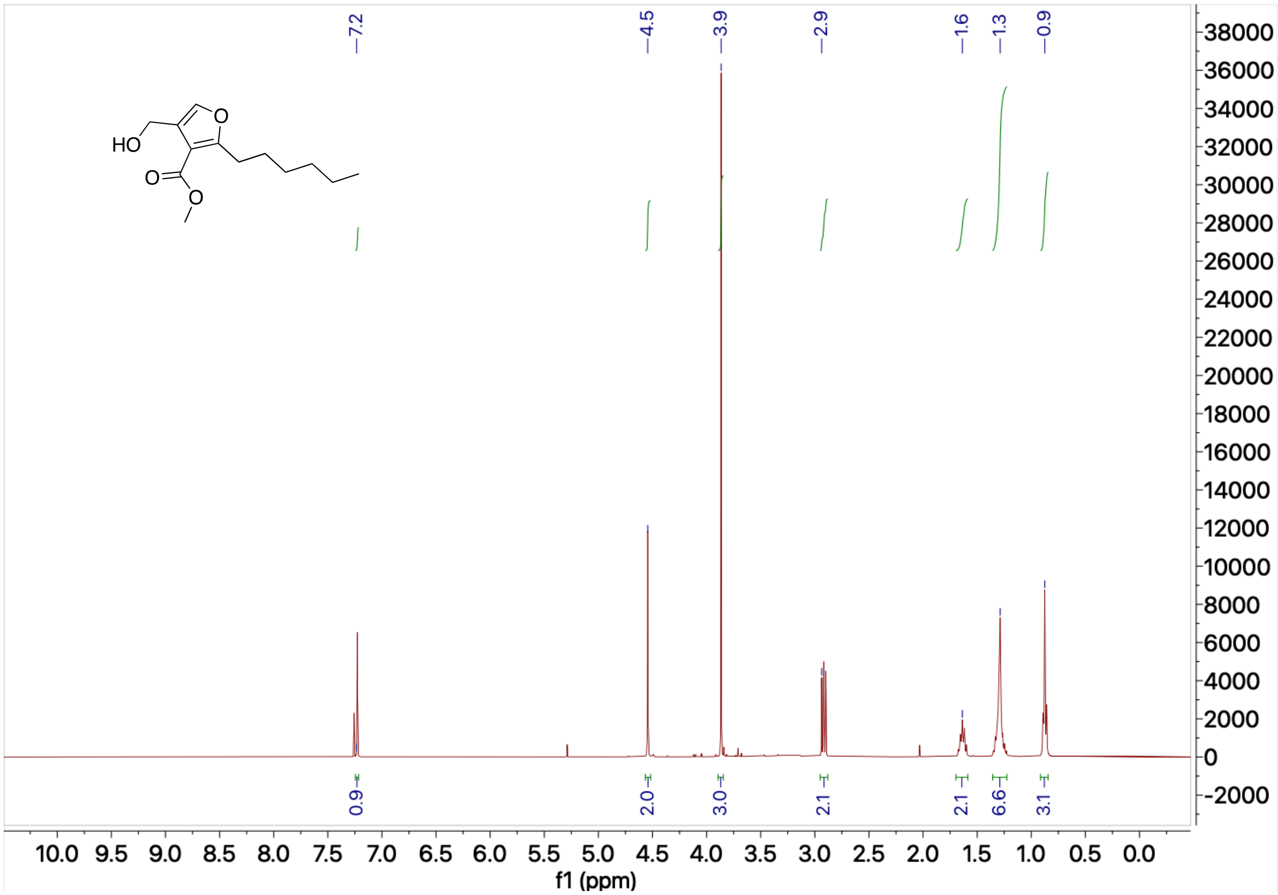
HO
O
O
O
7.2
4.5
3.9
2.9
1.6
1.3
0.9
0.9
2.0
3.0
2.1
2.1
6.6
3.1
f1 (ppm)

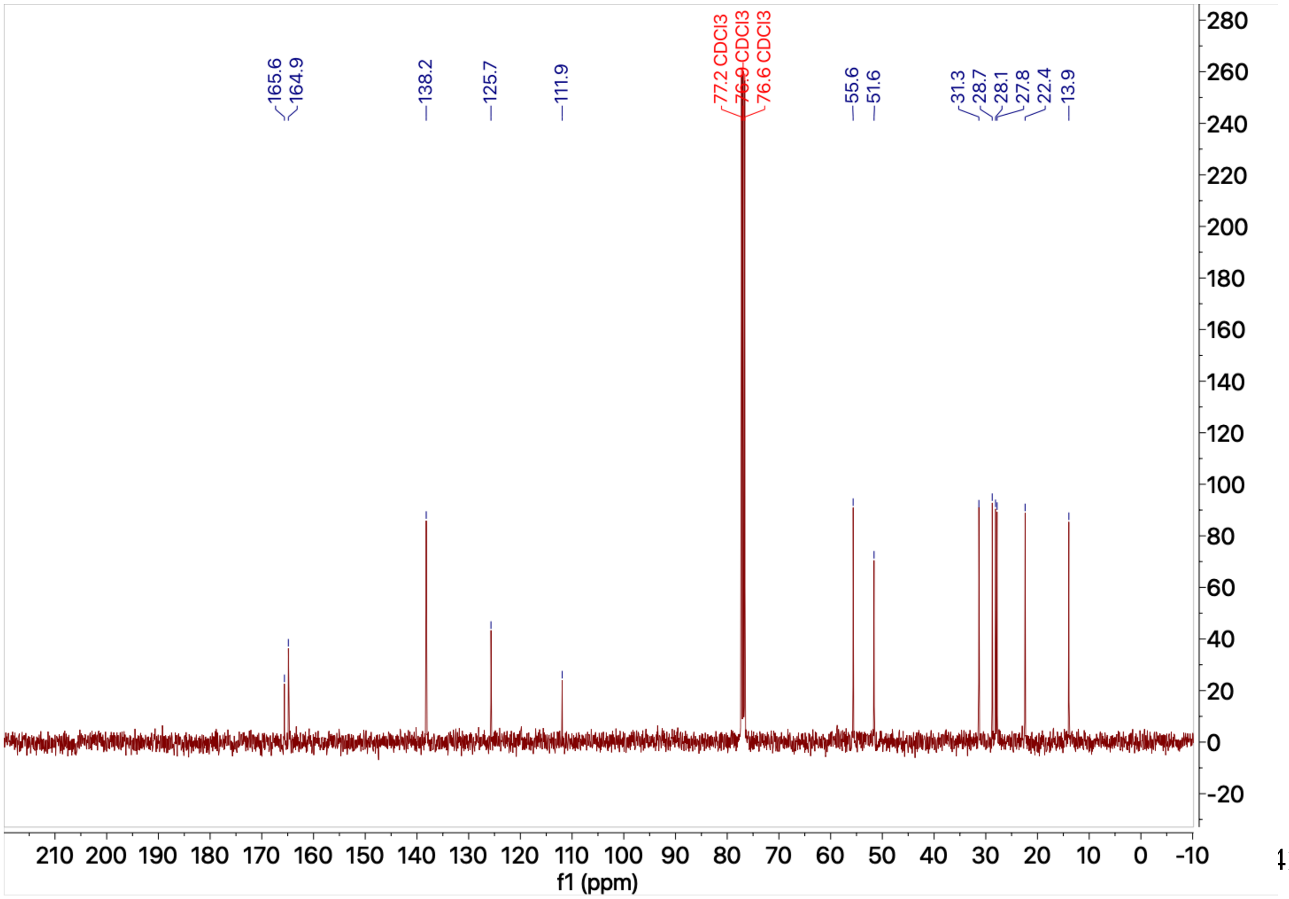
165.6
164.9
138.2
125.7
111.9
77.2 CDCl3
76.6 CDCl3
55.6
51.6
31.3
28.7
28.1
27.8
22.4
13.9
f1 (ppm)

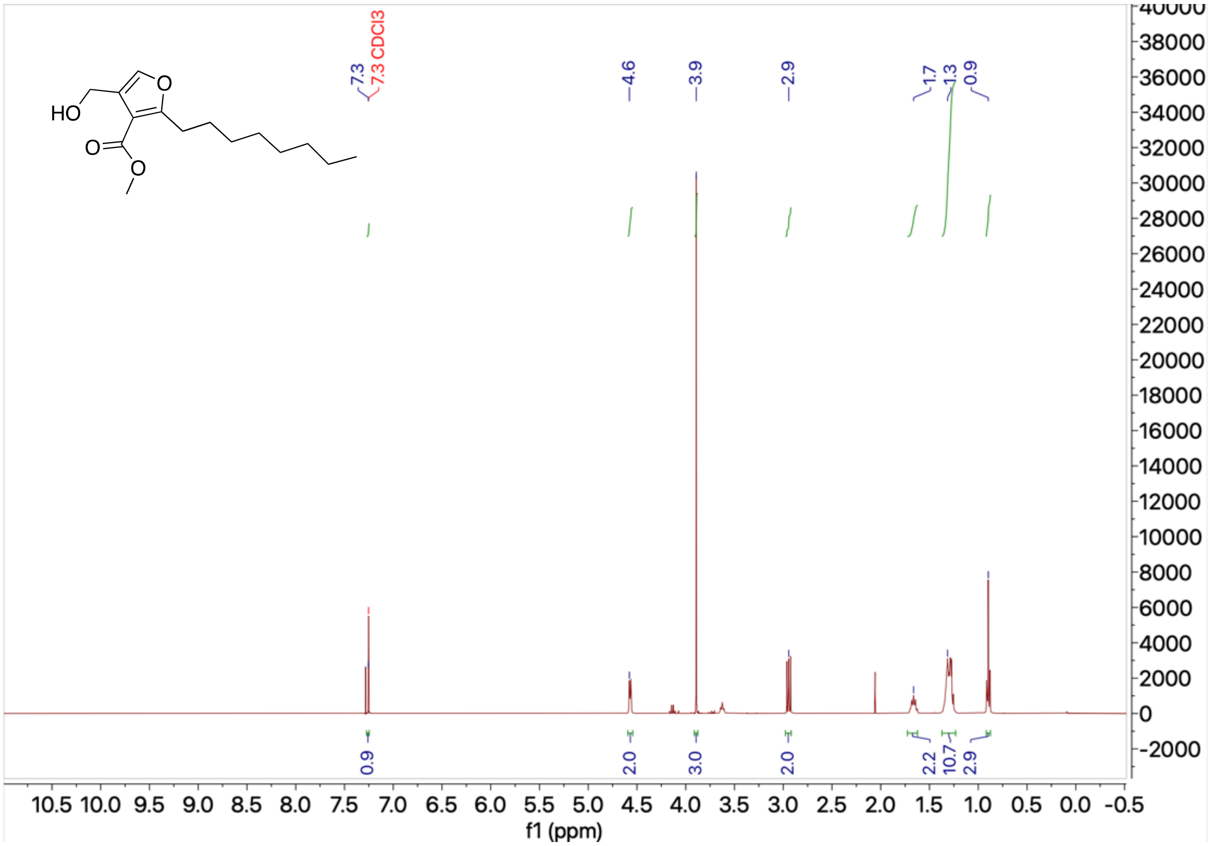
HO
O
O
O
7.3
7.3 CDCl3
4.6
3.9
2.9
1.7
1.3
0.9
0.9
2.0
3.0
2.0
2.2
10.7
2.9
f1 (ppm)

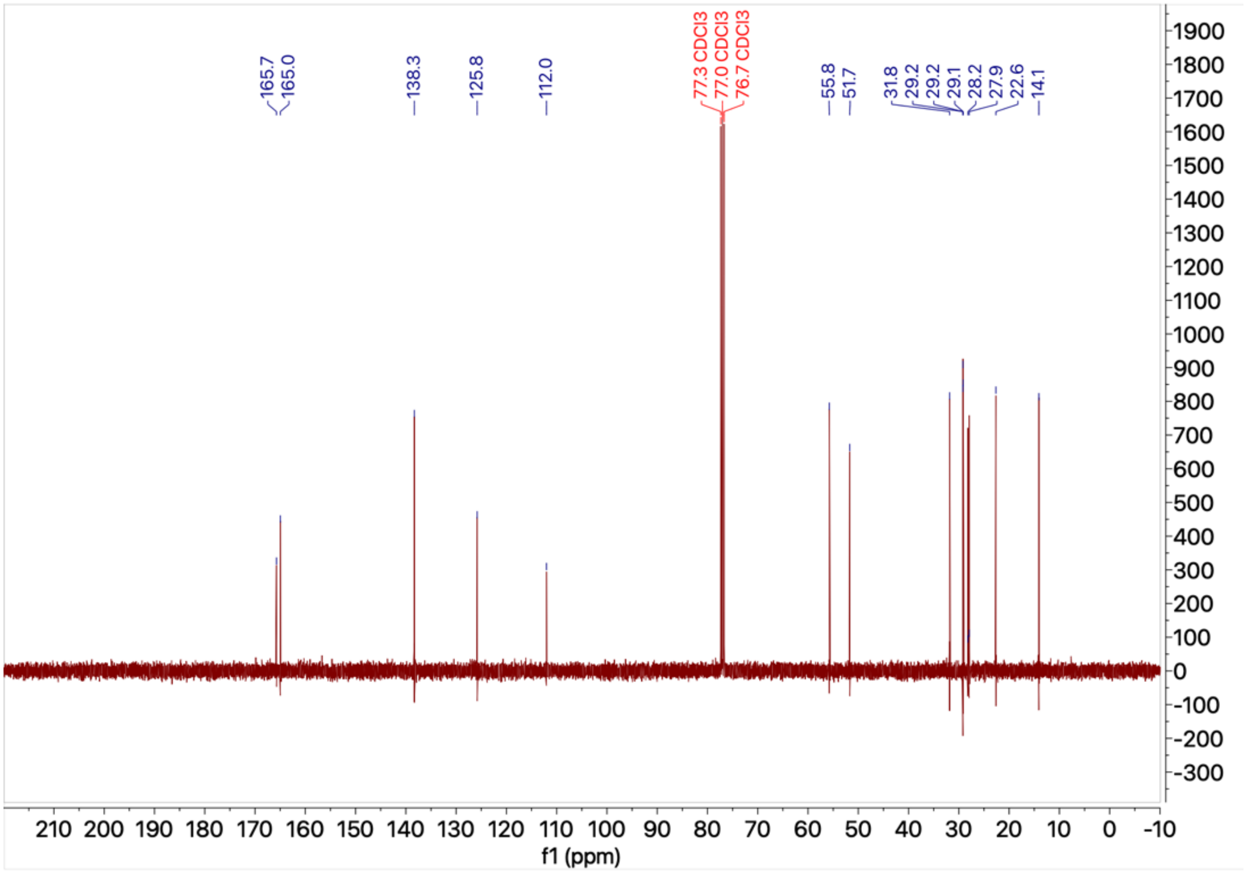
165.7
165.0
138.3
125.8
112.0
77.3 CDCl3
77.0 CDCl3
76.7 CDCl3
55.8
51.7
31.8
29.2
29.2
29.1
28.2
27.9
22.6
14.1
f1 (ppm)

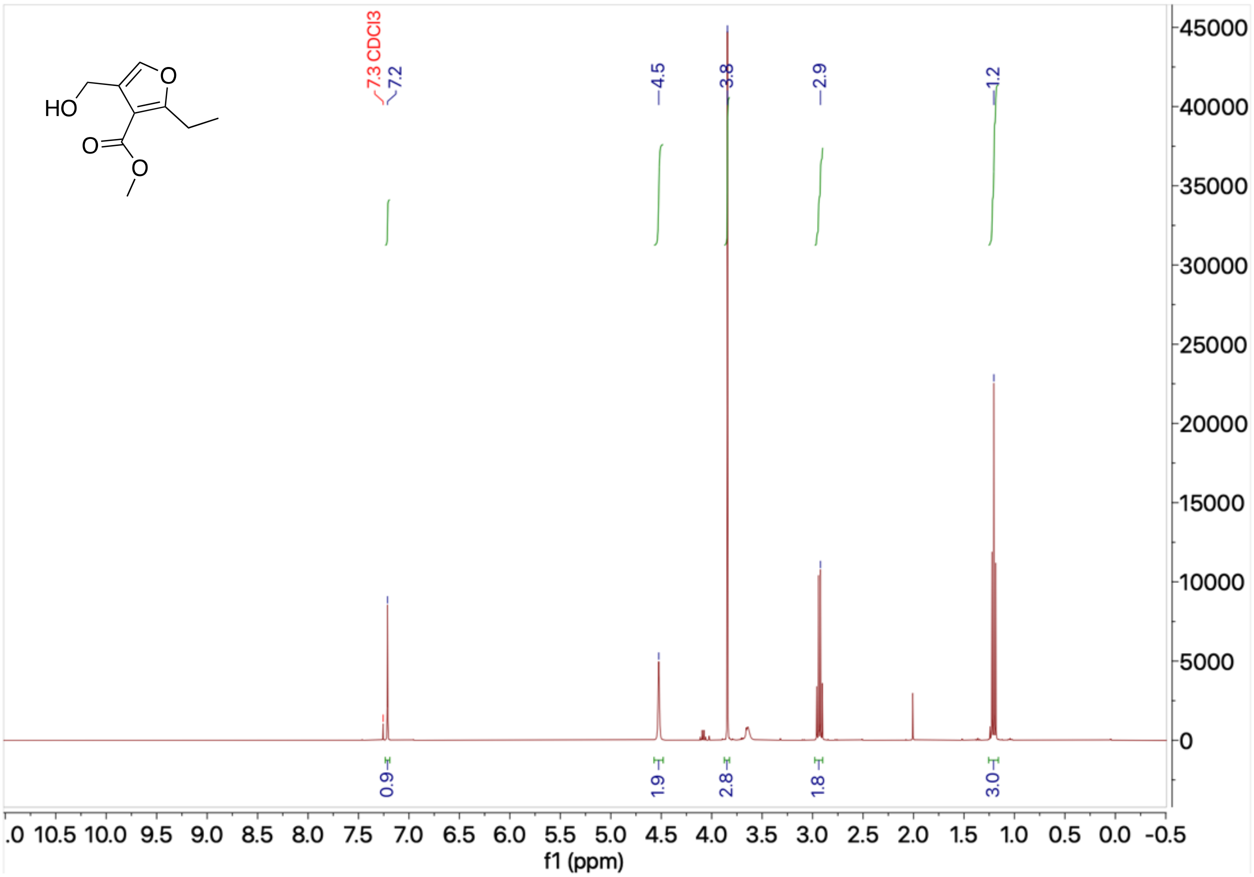
HO
O
O
O
7.3 CDCl3
7.2
4.5
3.8
2.9
1.2
0.9
1.9
2.8
1.8
3.0
f1 (ppm)

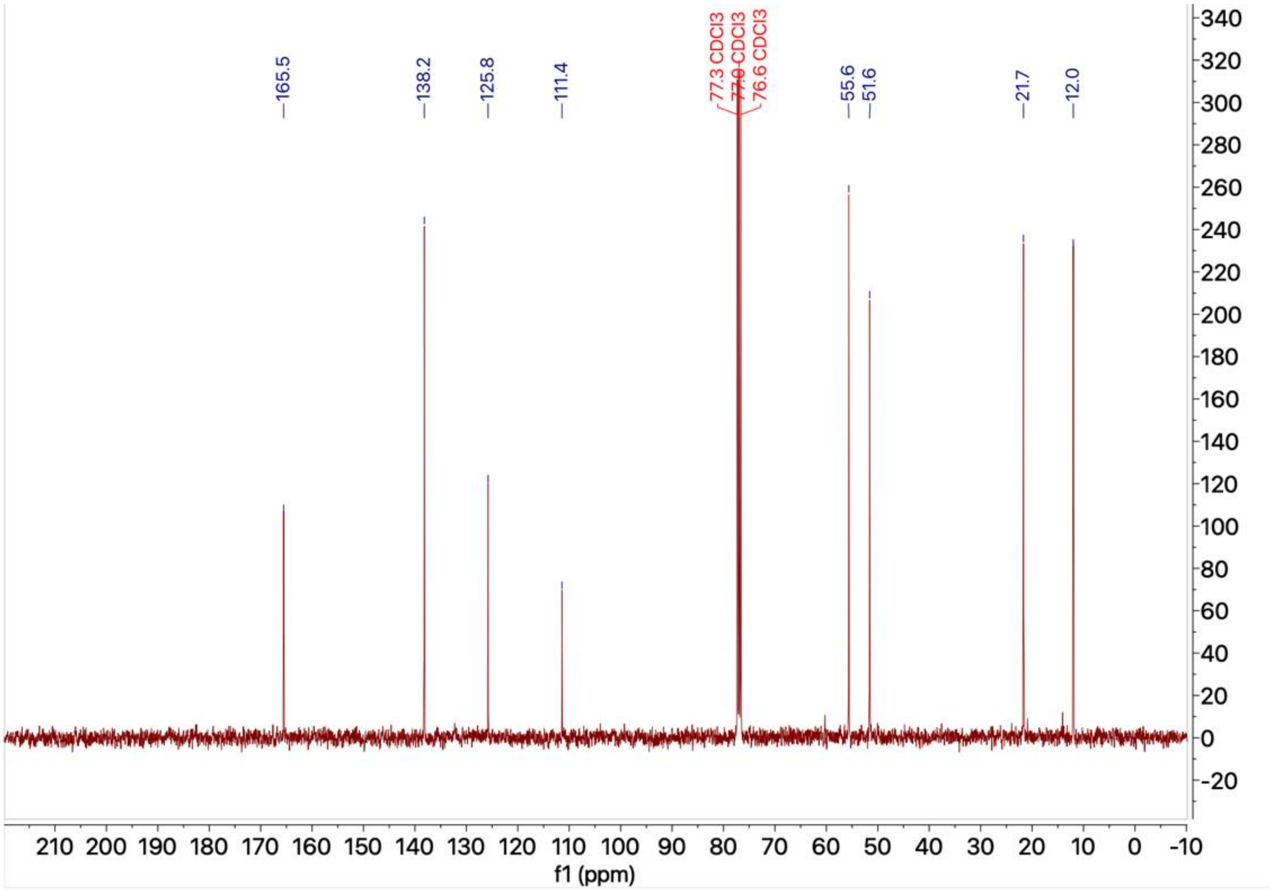
165.5
138.2
125.8
111.4
77.3 CDCl3
77.0 CDCl3
76.6 CDCl3
55.6
51.6
21.7
12.0
f1 (ppm)

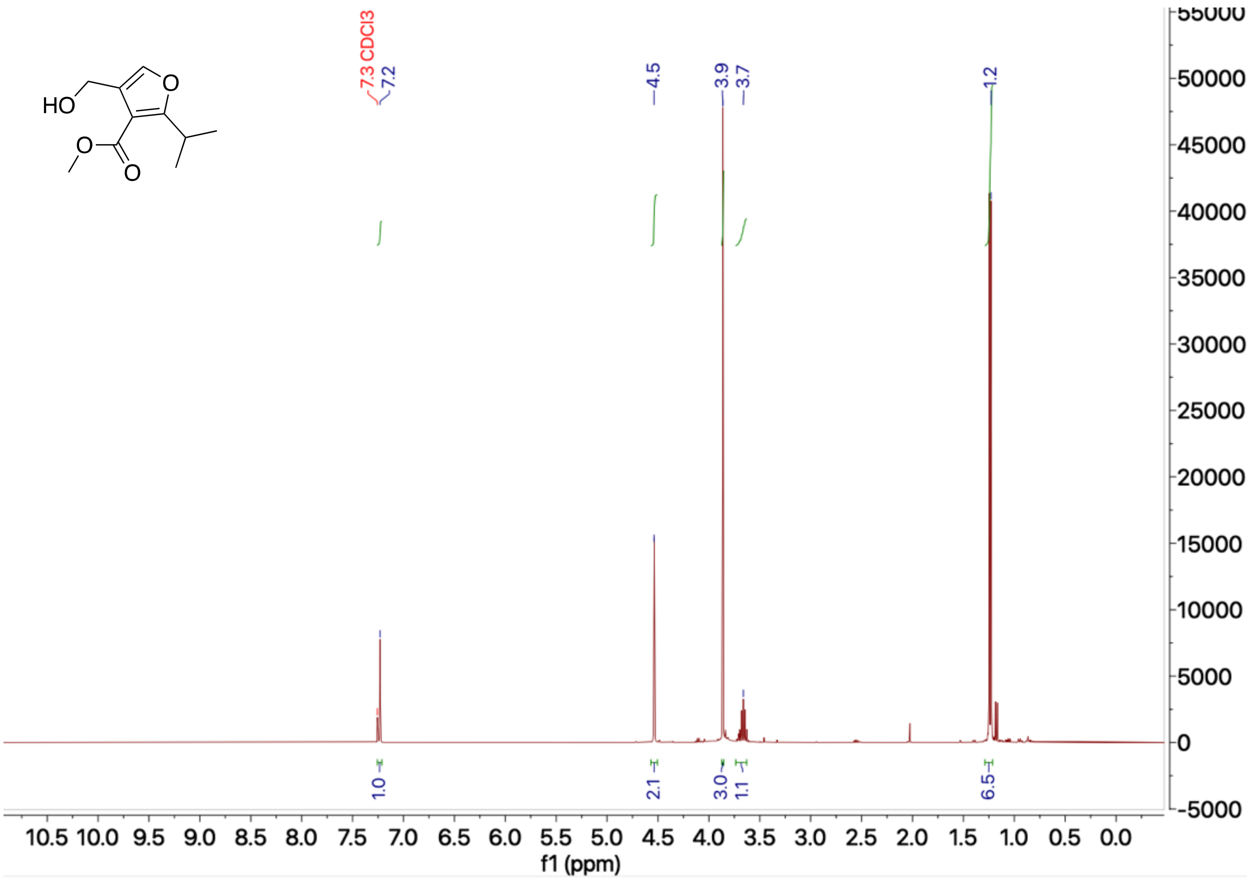

HO
O
O
O
7.3 CDCl3
7.2
4.5
3.9
3.7
1.2
1.0
2.1
3.0
1.1
6.5
f1 (ppm)

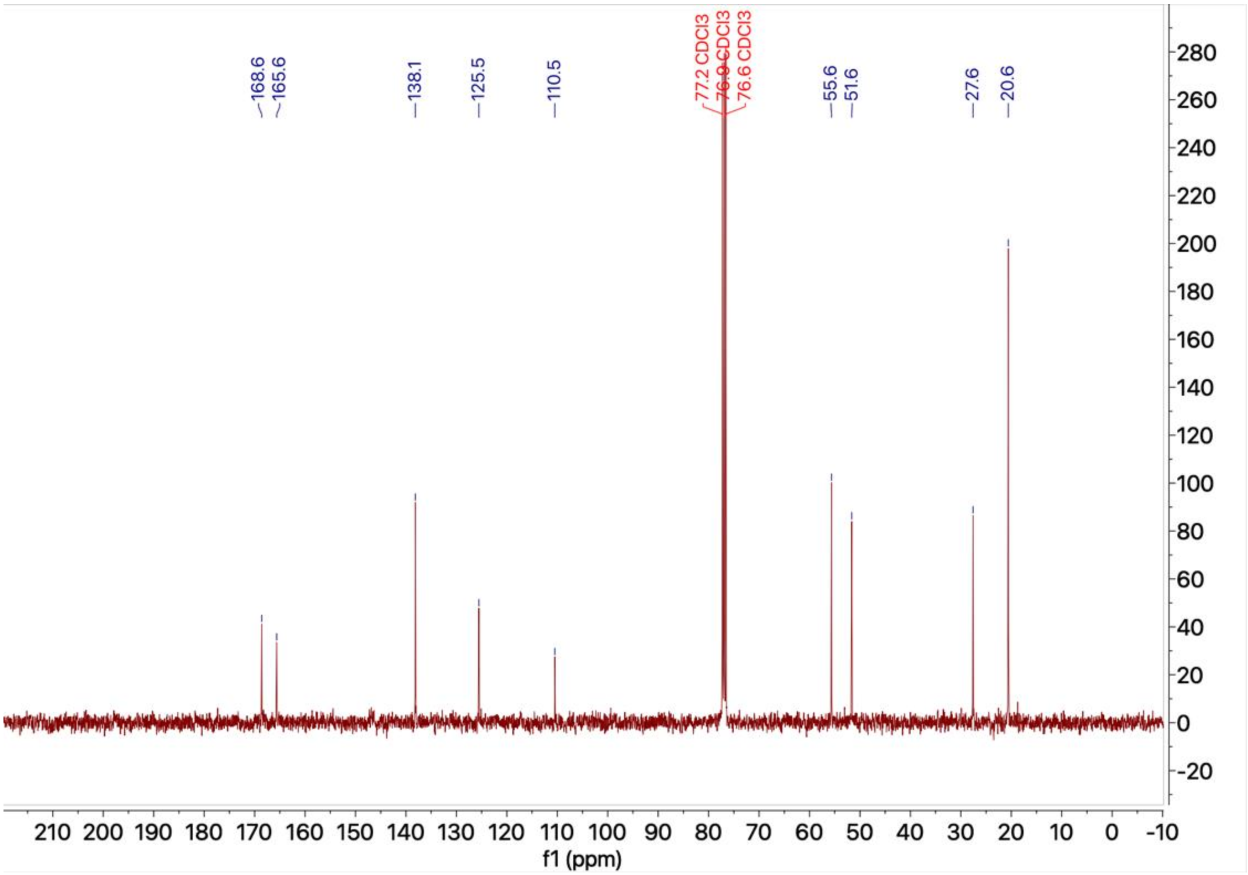

168.6
165.6
138.1
125.5
110.5
77.2 CDCl3
76.9 CDCl3
76.6 CDCl3
55.6
51.6
27.6
20.6
f1 (ppm)

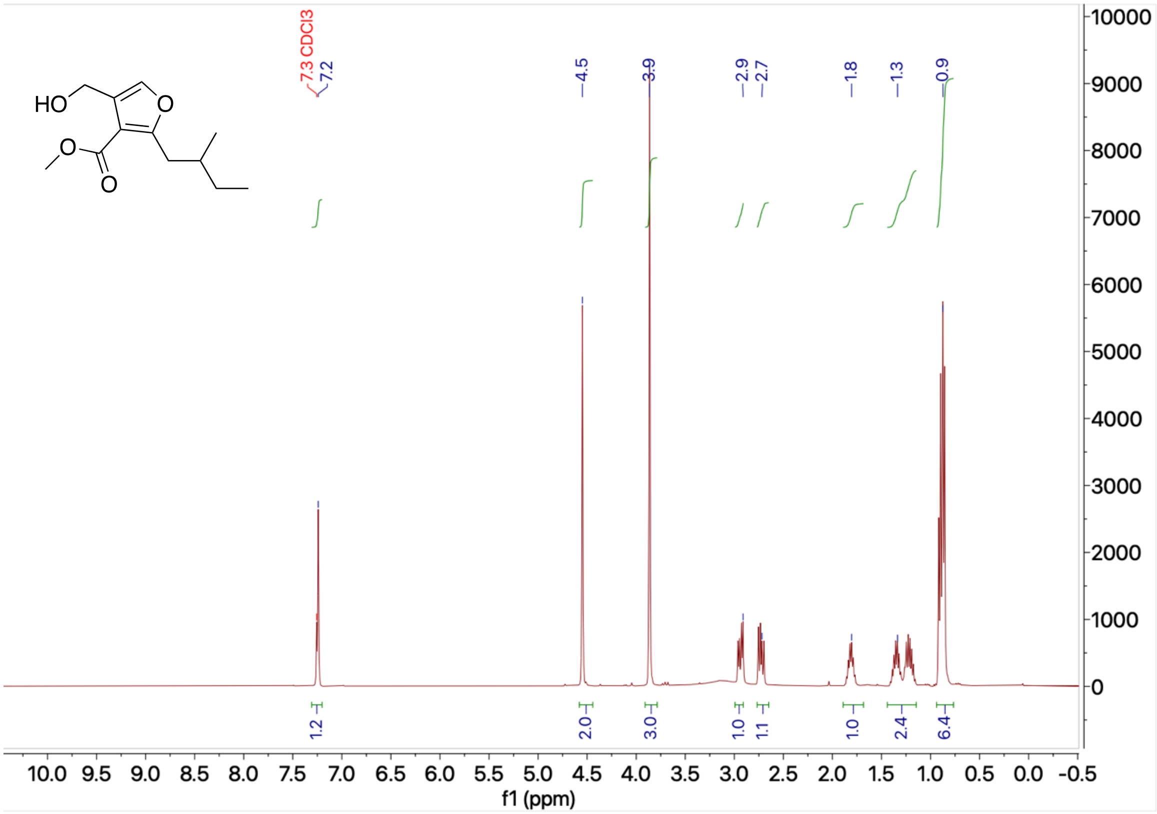
7.3 CDCl3
7.2
4.5
3.9
2.9
2.7
1.8
1.3
0.9
1.2
2.0
3.0
1.0
1.1
1.0
2.4
6.4
f1 (ppm)

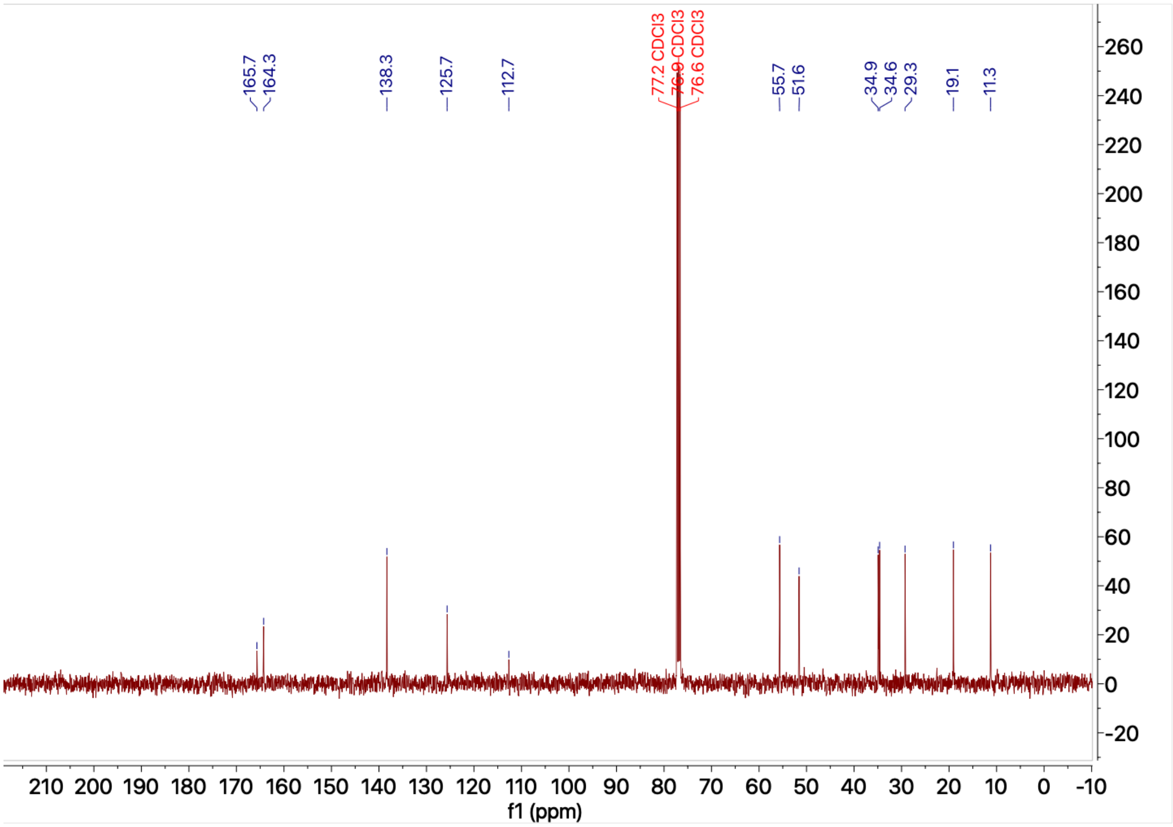
165.7
164.3
138.3
125.7
112.7
77.2 CDCl3
76.9 CDCl3
76.6 CDCl3
55.7
51.6
34.9
34.6
29.3
19.1
11.3
f1 (ppm)

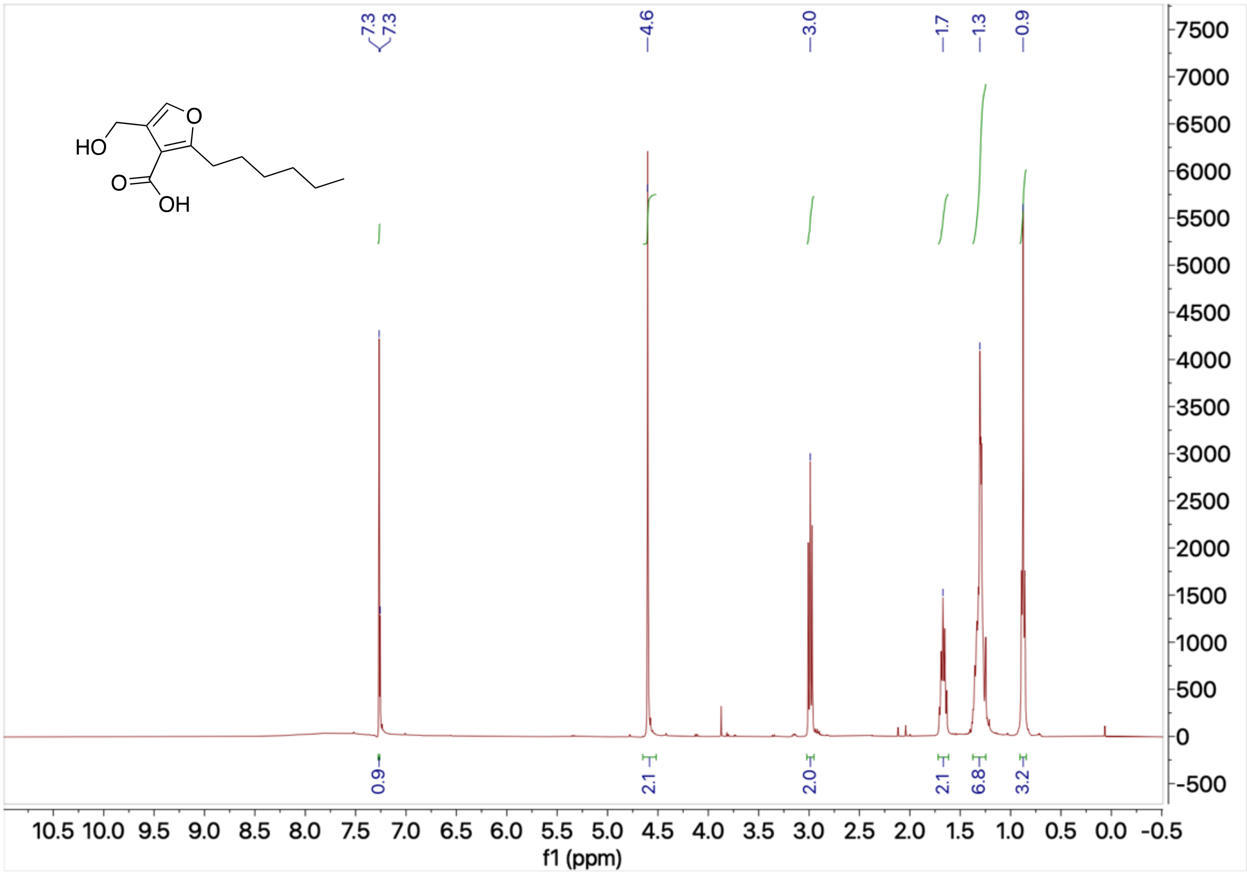

HO
O
O
OH
7.3
7.3
4.6
3.0
1.7
1.3
0.9
0.9
2.1
2.0
2.1
6.8
3.2
f1 (ppm)

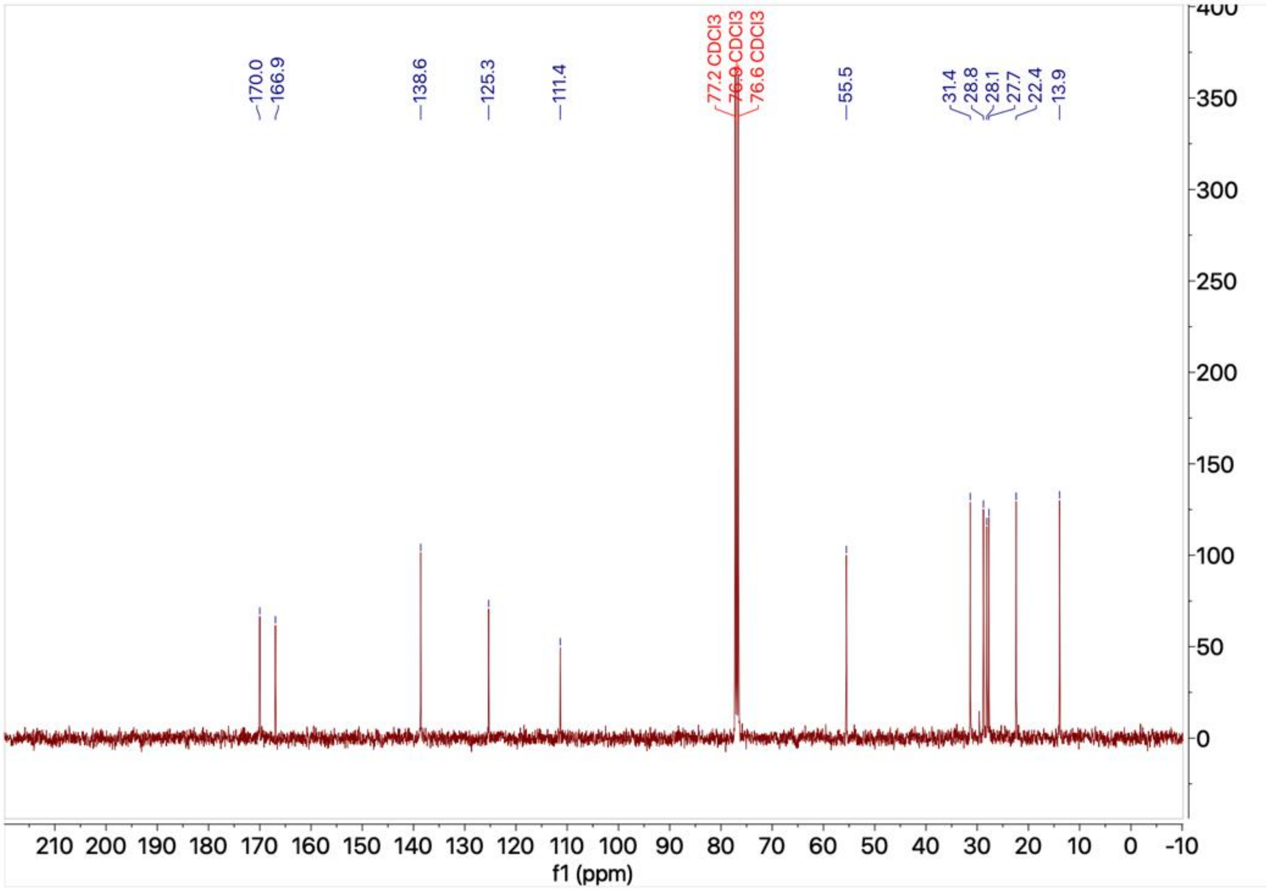

170.0
166.9
138.6
125.3
111.4
77.2 CDCl3
76.9 CDCl3
76.6 CDCl3
55.5
31.4
28.8
28.1
27.7
22.4
13.9
f1 (ppm)

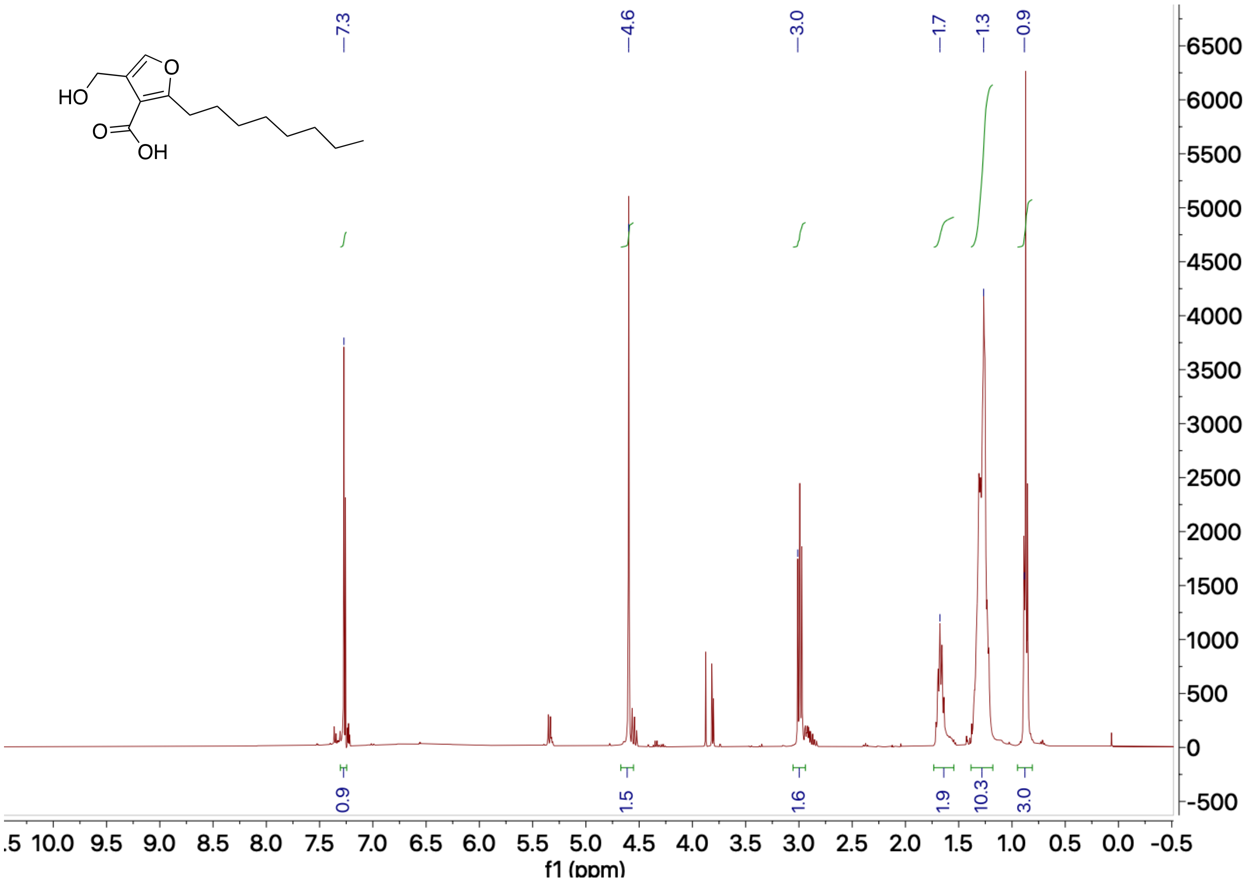
HO
O
OH
7.3
4.6
3.0
1.7
1.3
0.9
0.9
1.5
1.6
1.9
10.3
3.0
f1 (ppm)

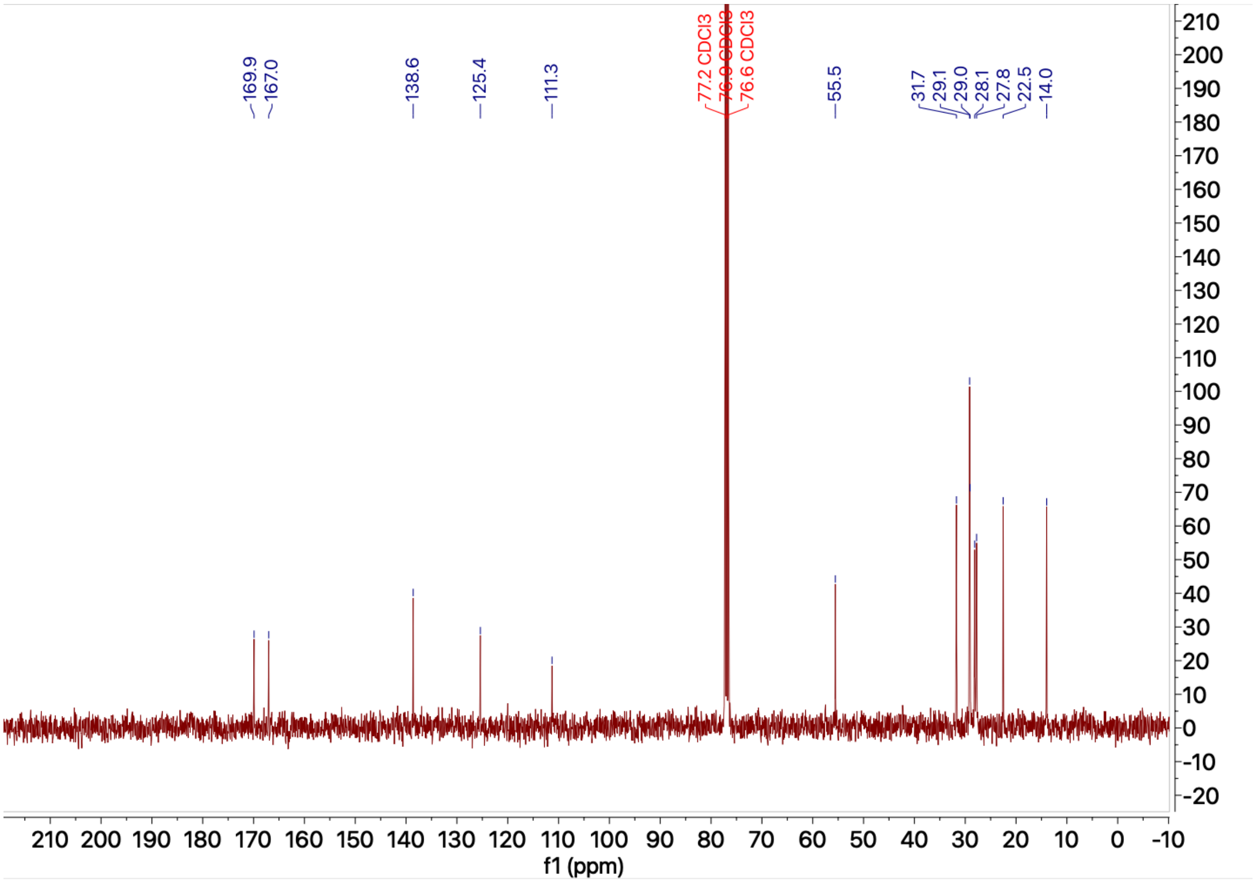
169.9
167.0
138.6
125.4
111.3
77.2 CDCl3
76.6 CDCl3
55.5
31.7
29.1
29.0
28.1
27.8
22.5
14.0
f1 (ppm)

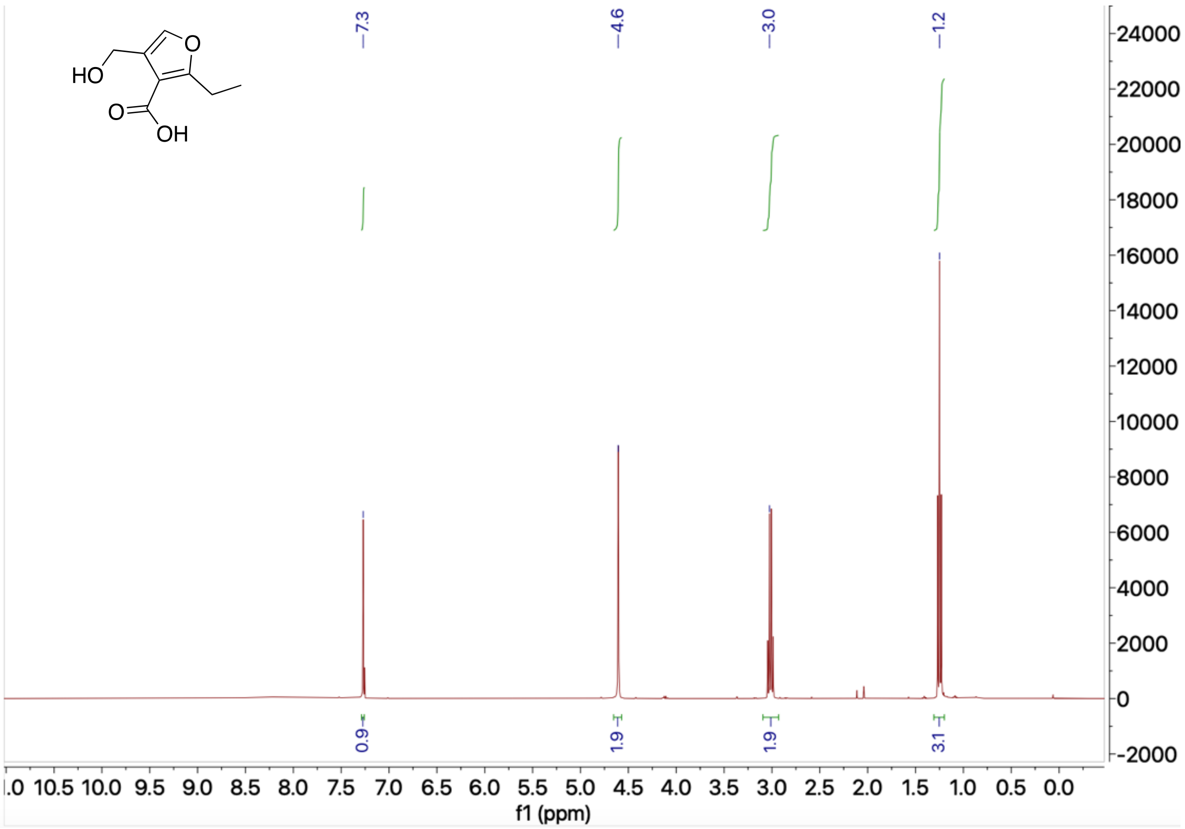
HO
O
O
OH
7.3
4.6
3.0
1.2
0.9
1.9
1.9
3.1
f1 (ppm)

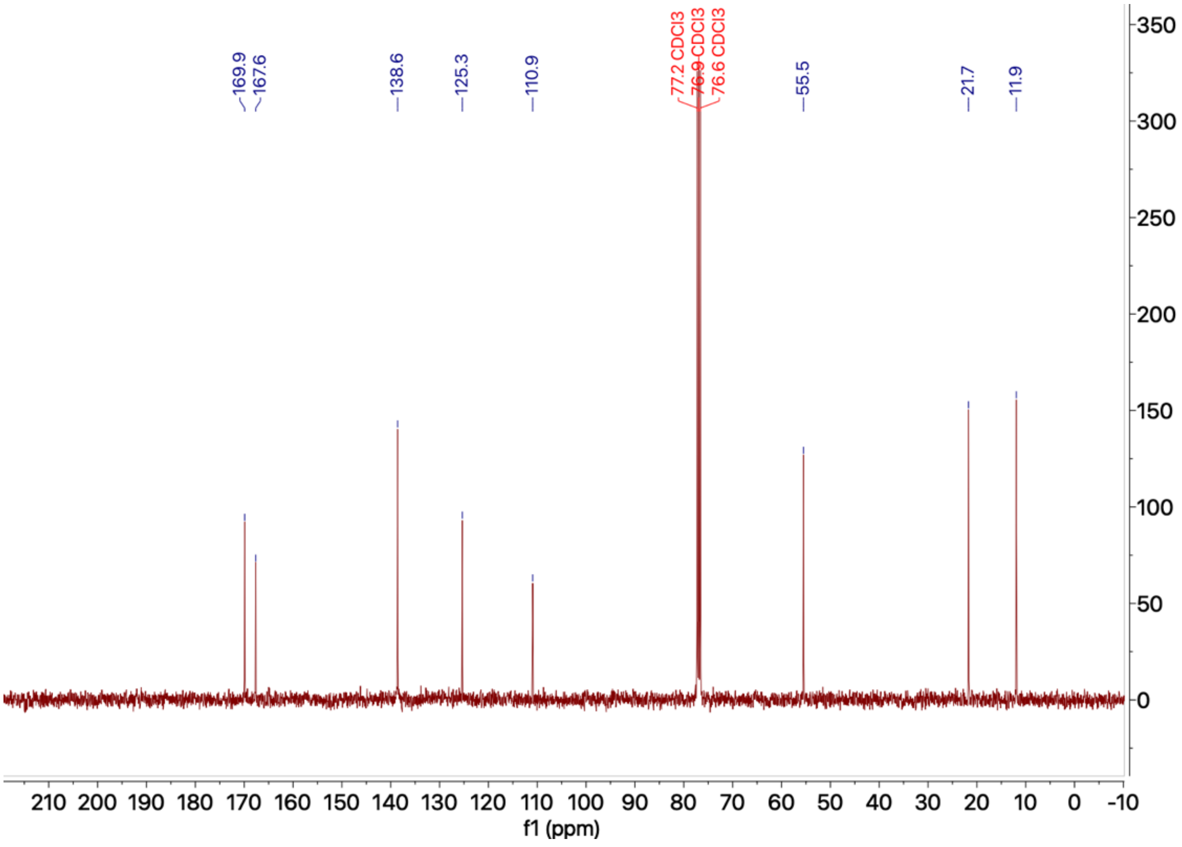
169.9
167.6
138.6
125.3
110.9
77.2 CDCl3
76.9 CDCl3
76.6 CDCl3
55.5
21.7
11.9
f1 (ppm)

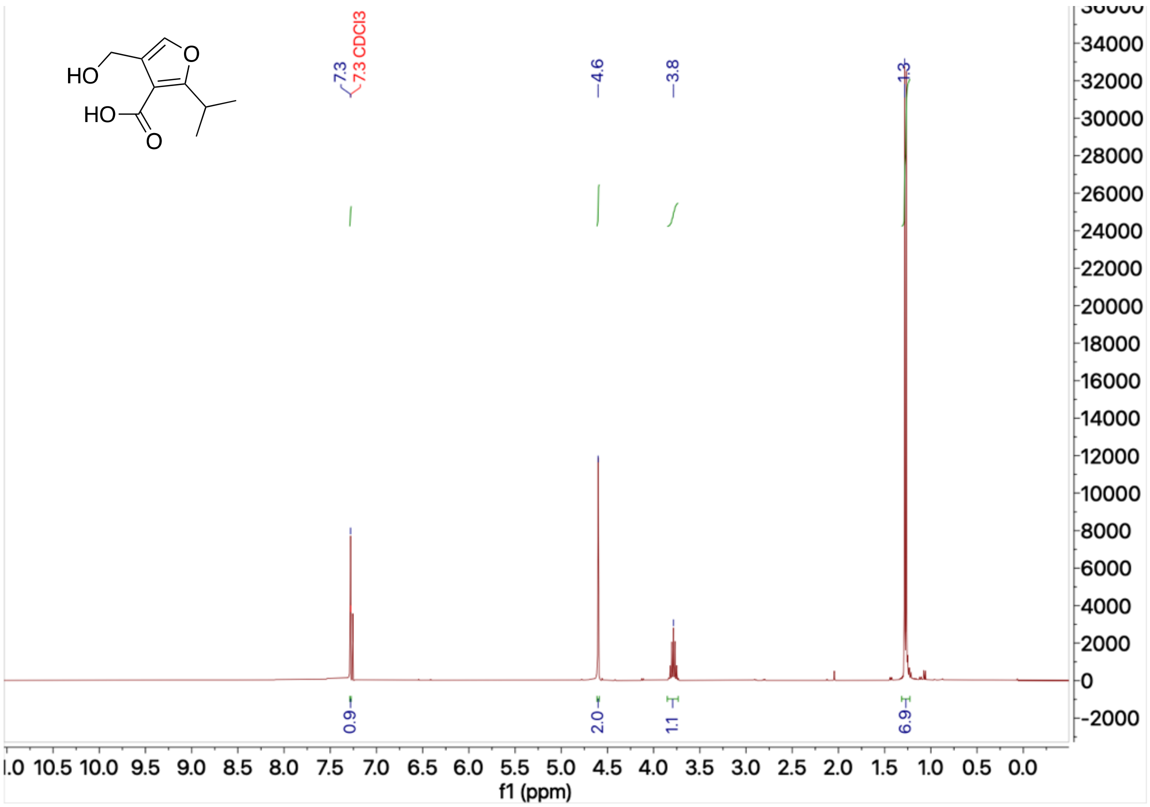
HO
HO
O
O
7.3
7.3 CDCl3
4.6
3.8
1.3
0.9
2.0
1.1
6.9
f1 (ppm)

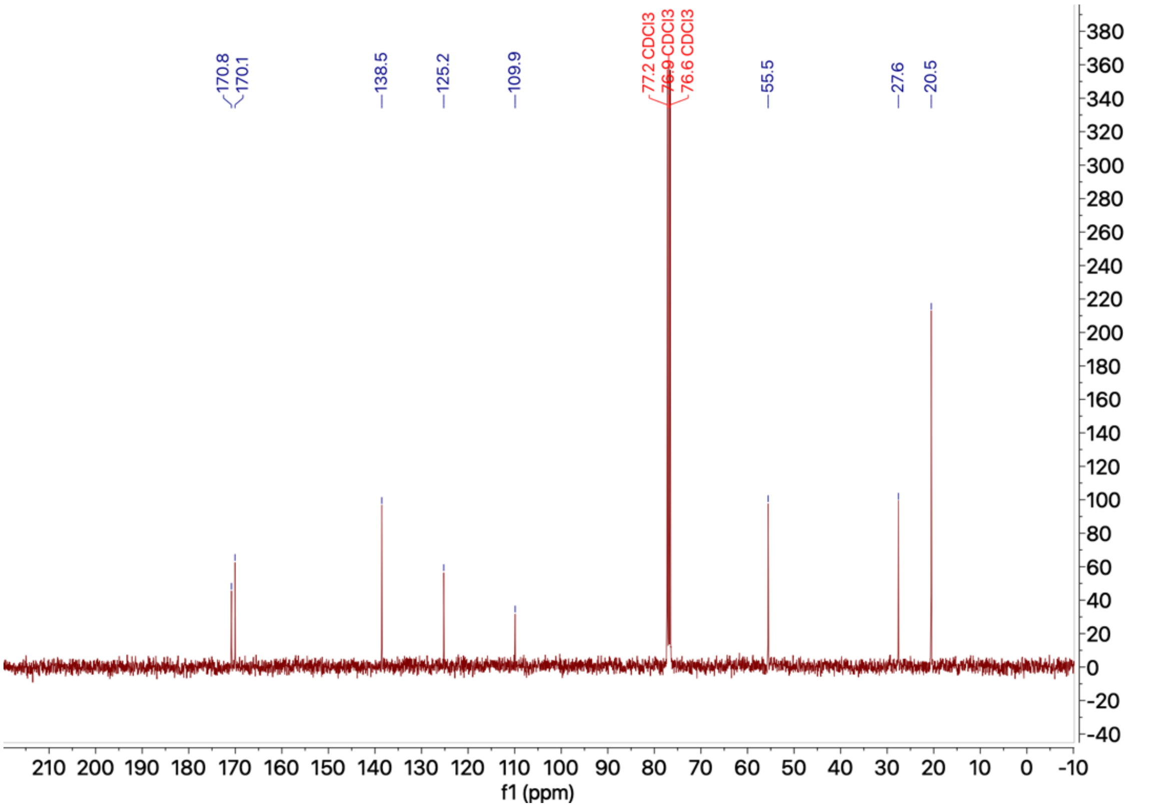
170.8
170.1
138.5
125.2
109.9
77.2 CDCl3
76.9 CDCl3
76.6 CDCl3
55.5
27.6
20.5
f1 (ppm)

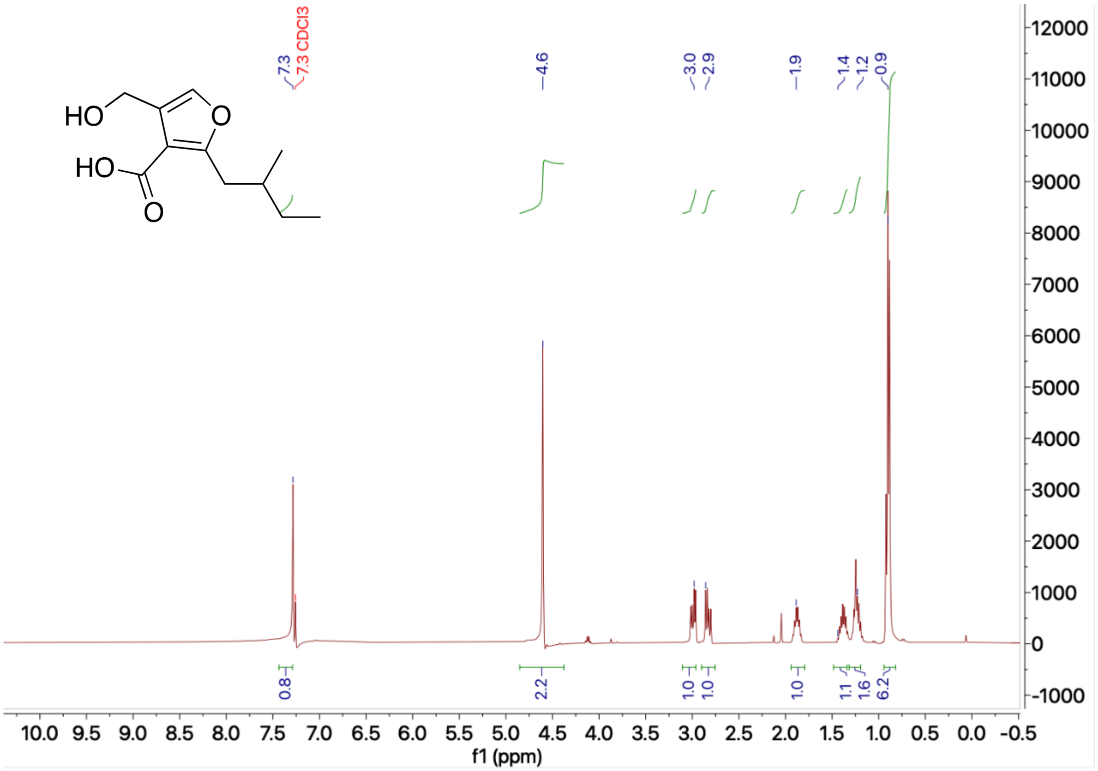
HO
O
HO
O
7.3
7.3 CDCl3
4.6
3.0
2.9
1.9
1.4
1.2
0.9
0.8
2.2
1.0
1.0
1.0
1.1
1.6
6.2
f1 (ppm)

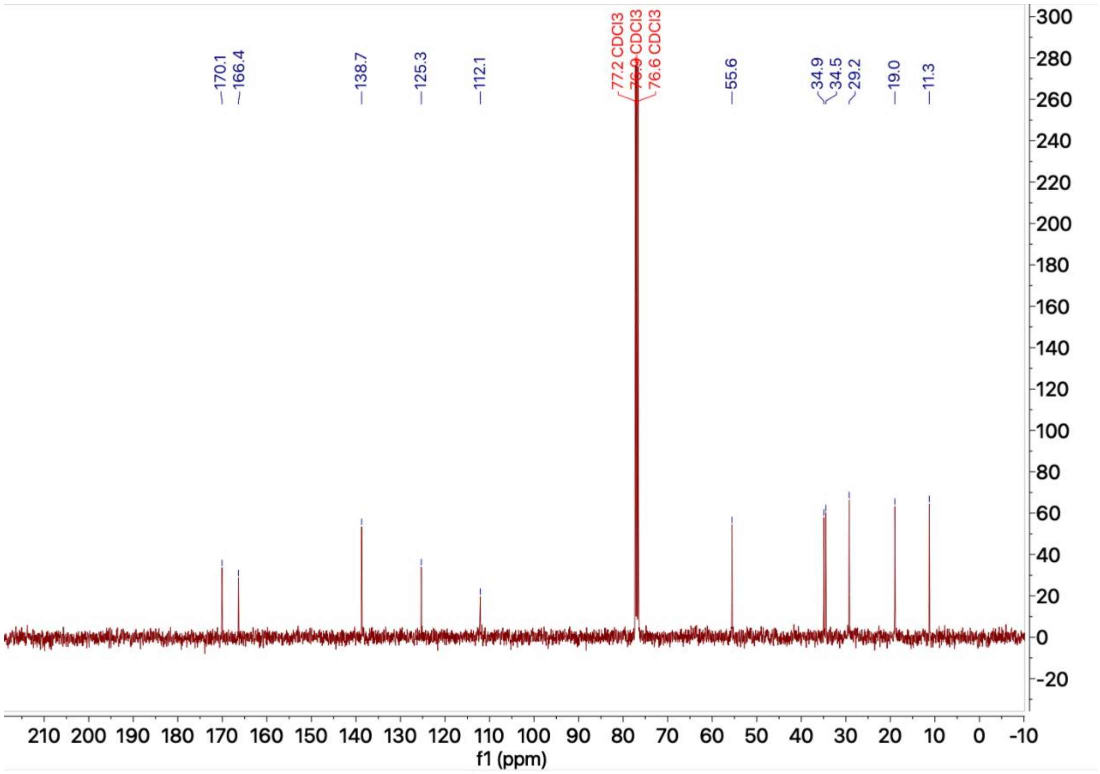
170.1
166.4
138.7
125.3
112.1
77.2 CDCl3
76.9 CDCl3
76.6 CDCl3
55.6
34.9
34.5
29.2
19.0
11.3
f1 (ppm)

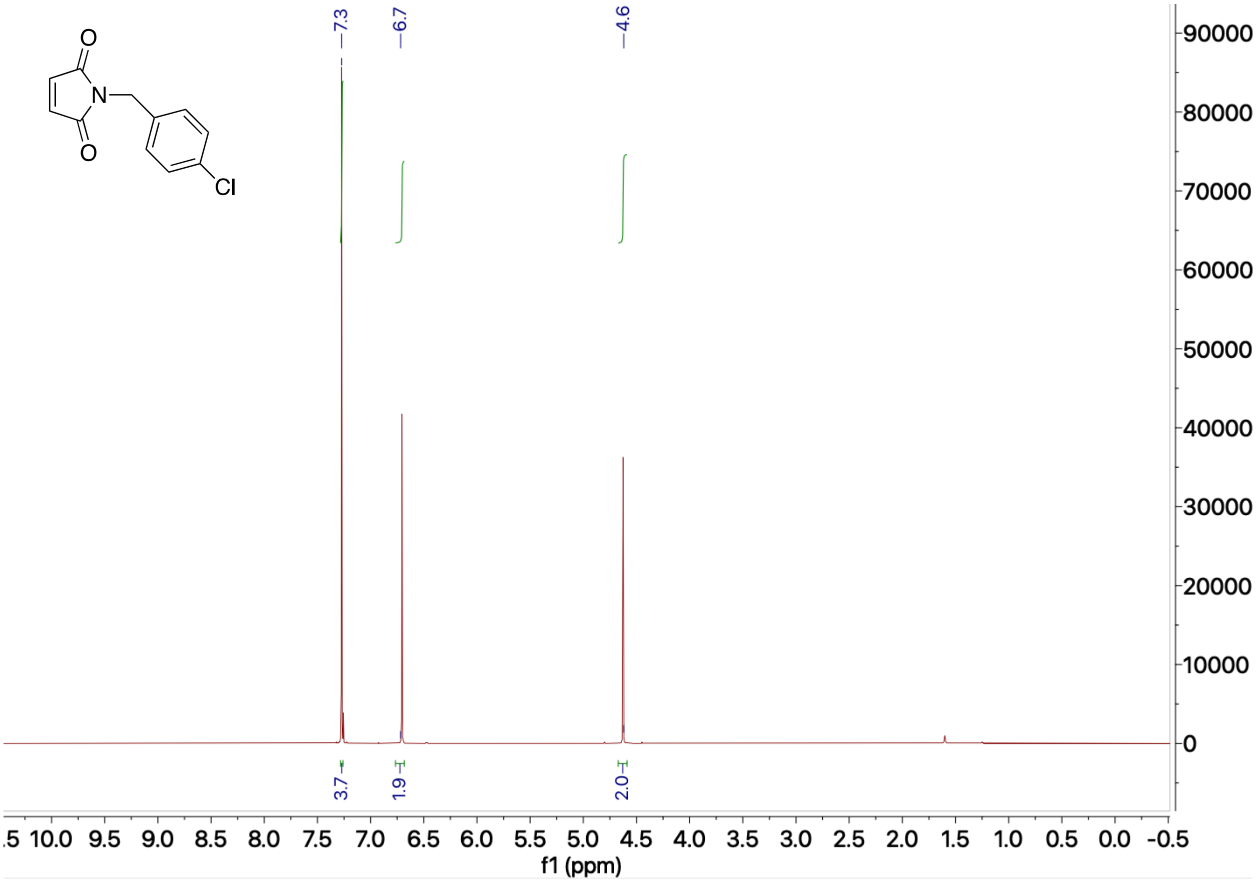
O
N
O
Cl
7.3
6.7
4.6
3.7
1.9
2.0
90000
80000
70000
60000
50000
40000
30000
20000
10000
0
.5 10.0 9.5 9.0 8.5 8.0 7.5 7.0 6.5 6.0 5.5 5.0 4.5 4.0 3.5 3.0 2.5 2.0 1.5 1.0 0.5 0.0 -0.5
f1 (ppm)

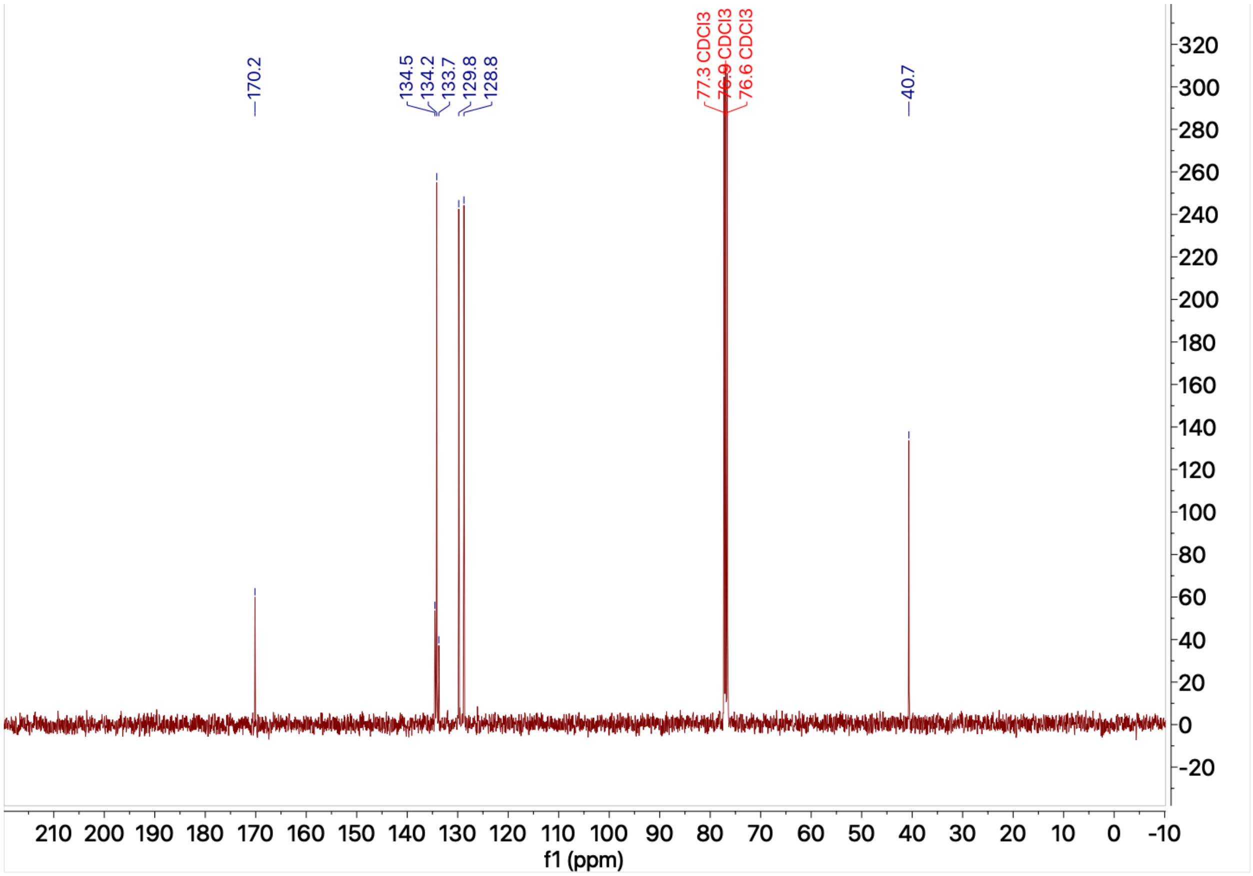
170.2
134.5
134.2
133.7
129.8
128.8
77.3 CDCl3
76.9 CDCl3
76.6 CDCl3
40.7
320
300
280
260
240
220
200
180
160
140
120
100
80
60
40
20
0
-20
210 200 190 180 170 160 150 140 130 120 110 100 90 80 70 60 50 40 30 20 10 0 -10
f1 (ppm)

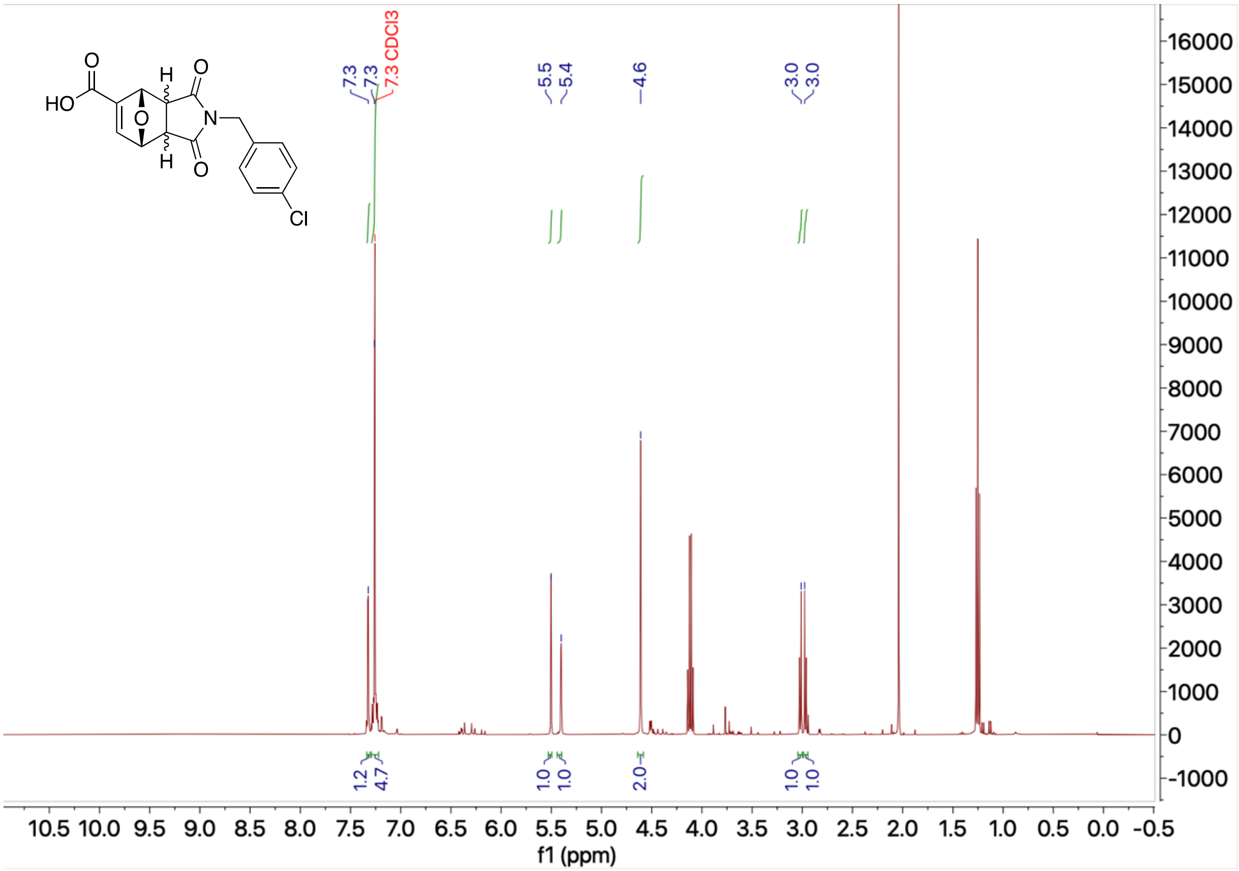

HO
O
H
H
O
O
N
O
Cl
7.3
7.3
7.3 CDCl3
5.5
5.4
4.6
3.0
3.0
1.2
4.7
1.0
1.0
2.0
1.0
1.0
f1 (ppm)

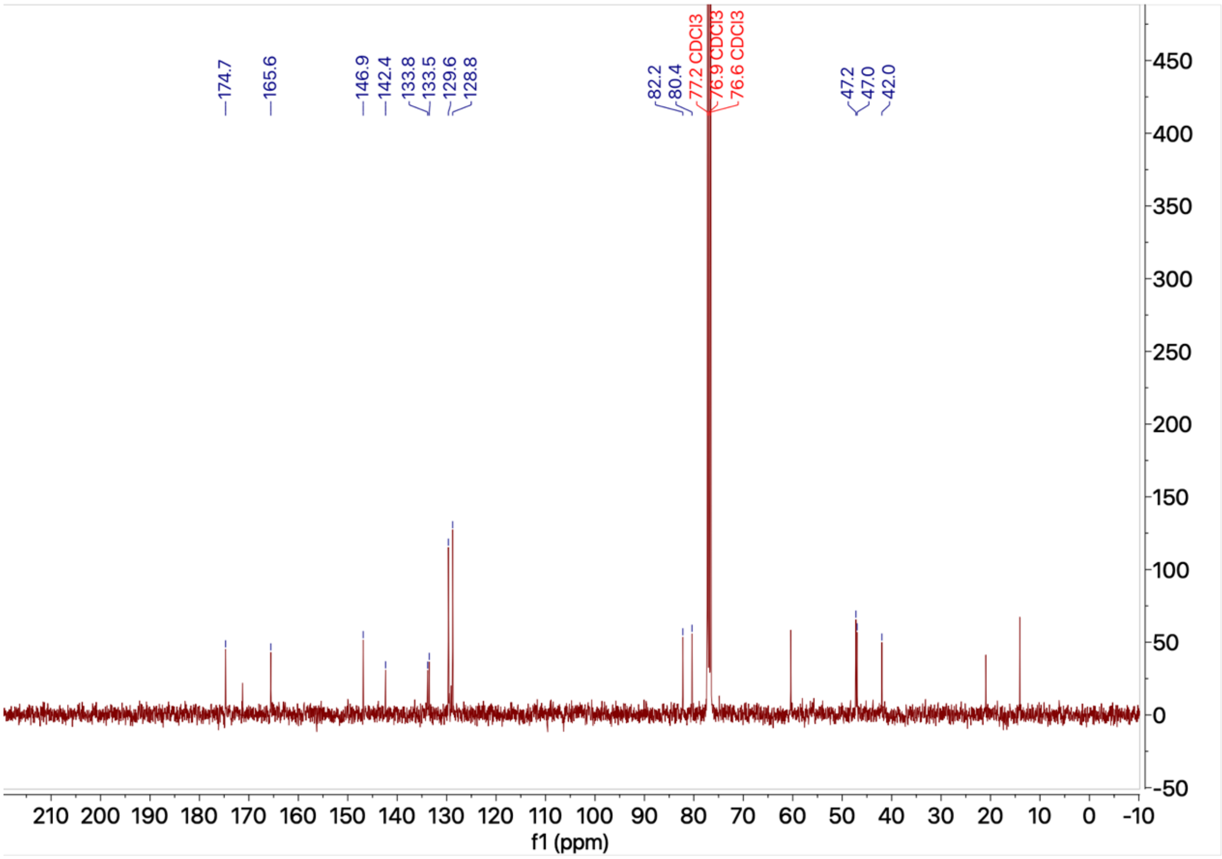

174.7
165.6
146.9
142.4
133.8
133.5
129.6
128.8
82.2
80.4
77.2 CDCl3
76.9 CDCl3
76.6 CDCl3
47.2
47.0
42.0
f1 (ppm)
